# A first-in-class selective inhibitor of EGFR and PI3K offers a single-molecule approach to targeting adaptive resistance

**DOI:** 10.1038/s43018-024-00781-6

**Published:** 2024-07-11

**Authors:** Christopher E. Whitehead, Elizabeth K. Ziemke, Christy L. Frankowski-McGregor, Rachel A. Mumby, June Chung, Jinju Li, Nathaniel Osher, Oluwadara Coker, Veerabhadran Baladandayuthapani, Scott Kopetz, Judith S. Sebolt-Leopold

**Affiliations:** 1 Department of Radiology, University of Michigan, Ann Arbor, MI USA; 2 MEKanistic Therapeutics, Inc., Ann Arbor, MI USA; 3 Department of Biostatistics, The University of Michigan School of Public Health, Ann Arbor, MI USA; 4 The University of Texas MD Anderson Cancer Center, Houston, TX USA; 5 University of Michigan Rogel Cancer Center, Ann Arbor, MI USA; 6 Department of Pharmacology, University of Michigan, Ann Arbor, MI USA

**Keywords:** Cancer, Drug discovery

## Abstract

Despite tremendous progress in precision oncology, adaptive resistance mechanisms limit the long-term effectiveness of molecularly targeted agents. Here we evaluated the pharmacological profile of MTX-531 that was computationally designed to selectively target two key resistance drivers, epidermal growth factor receptor and phosphatidylinositol 3-OH kinase (PI3K). MTX-531 exhibits low-nanomolar potency against both targets with a high degree of specificity predicted by cocrystal structural analyses. MTX-531 monotherapy uniformly resulted in tumor regressions of squamous head and neck patient-derived xenograft (PDX) models. The combination of MTX-531 with mitogen-activated protein kinase kinase or KRAS-G12C inhibitors led to durable regressions of *BRAF*-mutant or *KRAS*-mutant colorectal cancer PDX models, resulting in striking increases in median survival. MTX-531 is exceptionally well tolerated in mice and uniquely does not lead to the hyperglycemia commonly seen with PI3K inhibitors. Here, we show that MTX-531 acts as a weak agonist of peroxisome proliferator-activated receptor-γ, an attribute that likely mitigates hyperglycemia induced by PI3K inhibition. This unique feature of MTX-531 confers a favorable therapeutic index not typically seen with PI3K inhibitors.

## Abstract (web summary)

Sebolt-Leopold and colleagues design and develop a small-molecule inhibitor that can target both epidermal growth factor receptor and phosphatidylinositol 3-OH kinase, which can be leveraged to overcome resistance to targeted therapies in vivo.

## Main

Revolutionary advances in precision oncology have been enabled by genomic sequencing of individual cancers and approval of a multitude of small-molecule kinase inhibitors. However, adaptive resistance mechanisms compromise the long-term effectiveness of kinase-targeted therapies, dictating a need for combination strategies^1–3^. The traditional combination approach of administering multiple kinase inhibitors incurs added risks for off-target toxicities and imposes difficulties in achieving balanced and complete inhibition against the intended targets. An alternative approach is to rationally design small molecules inhibiting multiple resistance drivers that are sufficiently selective to avoid unacceptable toxicities.

Epidermal growth factor receptor (EGFR) and phosphatidylinositol 3-OH kinase (PI3K) emerge as potential cotargeting candidates to test this concept because these oncogenic kinases drive adaptive resistance across a broad spectrum of human cancers. In head and neck squamous cell carcinomas (HNSCCs), EGFR and PI3K are each known to mediate resistance to inhibition of the other^4,5^. Clinical activity of cetuximab, the only approved kinase-targeted therapy for this disease, is modest^6^. Molecular aberrations leading to a dysregulation of PI3K–mTOR (mammalian target of rapamycin) pathway signaling are found in up to 80% of HNSCCs and confer increased resistance to EGFR inhibition^7,8^. Treatment failures in the PI3K field have been attributed to unacceptable toxicities, in part driven by the need for high exposures to elicit monotherapy activity^9^. The PI3Kα inhibitor alpelisib (Piqray) is the only approved clinical agent in this target class based on its activity against *PIK3CA-*mutant advanced breast cancer. Both alpelisib and the pan-PI3K inhibitor copanlisib restore sensitivity to cetuximab in preclinical models of HNSCC^4,10^. However, benefits did not outweigh risks when these PI3K inhibitors were combined with cetuximab in the clinic^11,12^. Design of an efficacious PI3K inhibitor with an improved therapeutic index that additionally is not prone to EGFR-mediated adaptive resistance presents an area of high unmet medical need.

EGFR and PI3K pathway signaling has also been implicated in the adaptive resistance of *KRAS*-mutant and *BRAF*-mutant colorectal cancer (CRC) to RAS–MAPK (mitogen-activated protein kinase) pathway intervention. Whereas EGFR inhibitors alone are not indicated for the treatment of *KRAS*-mutant disease, preclinical findings support their use in combination with PI3K–mTOR or MEK (MAPK kinase) inhibitors to treat *KRAS*-mutant CRC^13,14^. In the case of *BRAF*-mutant CRC, a negative feedback activation loop activates EGFR in response to inhibition of BRAF, leading to reactivation of MAPK and PI3K pathway signaling^15–17^. A combination of encorafenib and cetuximab targeting BRAF and EGFR, respectively, has become the standard of care for treatment of *BRAF*^V600^ metastatic CRC^18^. Further addition of the PI3K inhibitor alpelisib to this regimen provides added efficacy but comes at the cost of increased toxicity^19,20^.

The central role of PI3K in oncogenic signaling and the toxicity challenges associated with its therapeutic targeting are reminiscent of those faced with RAS. For decades, RAS was considered undruggable until the breakthrough discovery of Shokat and colleagues reporting the design of covalent small-molecule inhibitors of KRAS-G12C (ref. ^21^). The present study was undertaken to design a PI3K inhibitor that would be better tolerated than previously reported molecules in this target class and also be highly selective for both PI3K and EGFR. The combinatorial potential of such an agent for the treatment of *KRAS*-mutant cancers is noteworthy.

Here, we provide evidence for the feasibility of computationally designing a dual inhibitor of EGFR and PI3K that is highly selective for its intended targets. Preclinical proof-of-concept studies carried out with MTX-531 ((*R*)-*N*-(2-chloro-5-(4-((1-phenylethyl)amino)quinazolin-6-yl)pyridin-3-yl)methane-sulfonamide) showed it to be highly efficacious as a single agent to treat HNSCC. Furthermore, we show that MTX-531 improves therapeutic outcome in combination with RAS pathway intervention in *BRAF*-mutant and *KRAS*-mutant CRC and pancreatic cancer. MTX-531 regimens were well tolerated and efficacious over a wide range of doses in preclinical models. In addition, MTX-531 regimens did not produce a hyperglycemic response as commonly seen with PI3K inhibitors. Collectively, preclinical data suggest that MTX-531 may be better tolerated in the clinic compared to previous PI3K inhibitors.

## Results

### MTX-531 is a potent and selective inhibitor of EGFR and PI3K

**Fig. 1:**
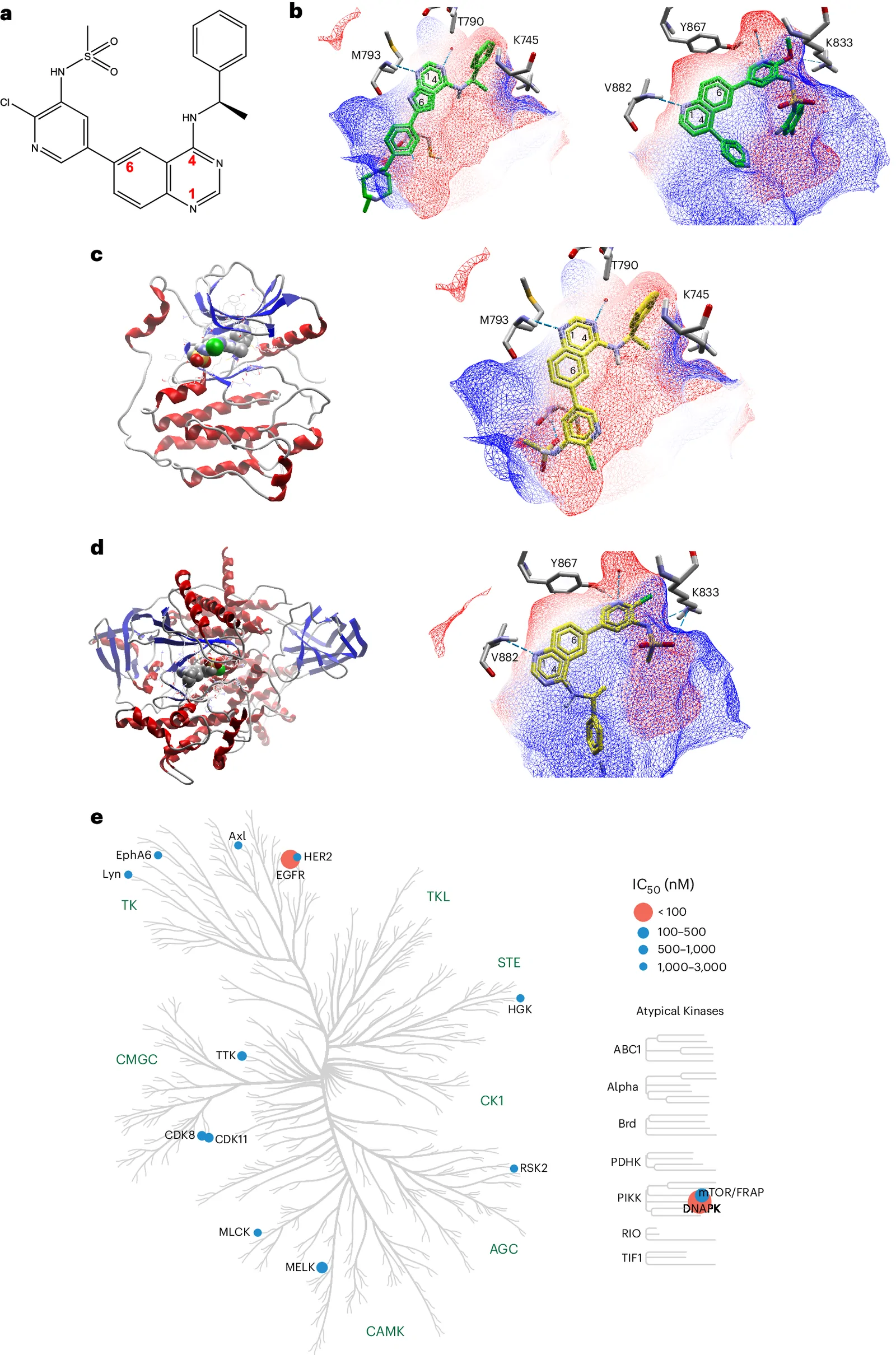
MTX-531 is a potent and selective inhibitor of EGFR and PI3K in vitro. **a**, Chemical structure of MTX-531. **b**, Structural features of NVP-AEE788 bound to EGFR (left) and omipalisib bound to PI3Kγ (right). The computational design of MTX-531 was based on an analysis of X-ray crystal structures of these known kinase inhibitors bound to their respective targets. **c**, Crystal structure of EGFR cocomplexed with MTX-531 (PDB 8SC7) solved at 2.0 Å. Left, the secondary structure; right, a view of MTX-531 bound to EGFR from the ATP-binding site. Graphics were generated by Molegro Virtual Docker 5.5. **d**, Crystal structure of PI3Kγ cocomplexed with MTX-531 (PDB 8SC8) solved at 2.7 Å. Left, the secondary structure; right, a view of MTX-531 bound to PI3Kγ from the ATP-binding site. Graphics were generated by Molegro Virtual Docker 5.5. **e**, Kinase selectivity of MTX-531. Selectivity was determined against a panel of 482 protein and lipid kinases by carrying out single-point testing of MTX-531 at a final concentration of 10 µM in duplicate assays. Kinases inhibited by >80% at 10 µM were retested in dose–response assays at concentrations tested in duplicate. Depicted here are kinases inhibited by 50% at concentrations ≤ 3 µM. This illustration was reproduced courtesy of Cell Signaling Technology (www.cellsignal.com)^26^. Source data

A structure-based drug design strategy was implemented to identify MTX-531 (Fig. 1a). Small molecules were rationally designed to inhibit both EGFR and PI3K in a selective and concurrent manner based on an analysis of known structural features of NVP-AEE788 (ref. ^22^) and omipalisib^23^ bound to EGFR and PI3Kγ, respectively (Fig. 1b). Whereas the 6-position of the fused ring of NVP-AEE788 points out toward the solvent, this position in omipalisib extends toward the back of the adenosine triphosphate (ATP) pocket of PI3Kγ and the specificity pocket. We leveraged this flipped binding mode of the quinazoline core between EGFR and PI3Kγ to computationally design potent and selective dual inhibitors of both enzyme families. Pivotal structure–activity relationship findings leading to MTX-531 are outlined in Supplementary Tables 1 and 2. X-ray crystal structures of EGFR and PI3Kγ cocomplexed with MTX-531 confirmed the postulated reversed binding mode of MTX-531 to each target. The 1-position of the quinazoline ring in MTX-531 forms a key hydrogen bond with the backbone amide of M793 of EGFR (Fig. 1c). The substitution at the 4-position fits into a hydrophobic pocket formed by the aliphatic side chains of K745 and T790, whereas the hydrophilic group at the 6-position faces outward to a solvent-accessible area of EGFR. The 1-position of MTX-531 forms a hydrogen bond with the backbone amide of V882 of PI3Kγ (Fig. 1d). Unlike the interactions seen with EGFR, groups at the 6-position of MTX-531 bind within a hydrophilic region of PI3K created by hydrophilic hydroxyl and amine groups of Y867 and K833, respectively. All studies described here were carried out with the (*R*)-isomer, which is ~100-fold more potent against EGFR and ~10-fold less potent against PI3K than the (*S*)-isomer. Collectively, the observed binding mode is consistent with these isomeric potency differences. The methyl substituent of MTX-531 in the (*S*)-isomer is oriented toward a sterically restricted area of EGFR, thus hindering binding.

**Extended Data Fig. 1:**
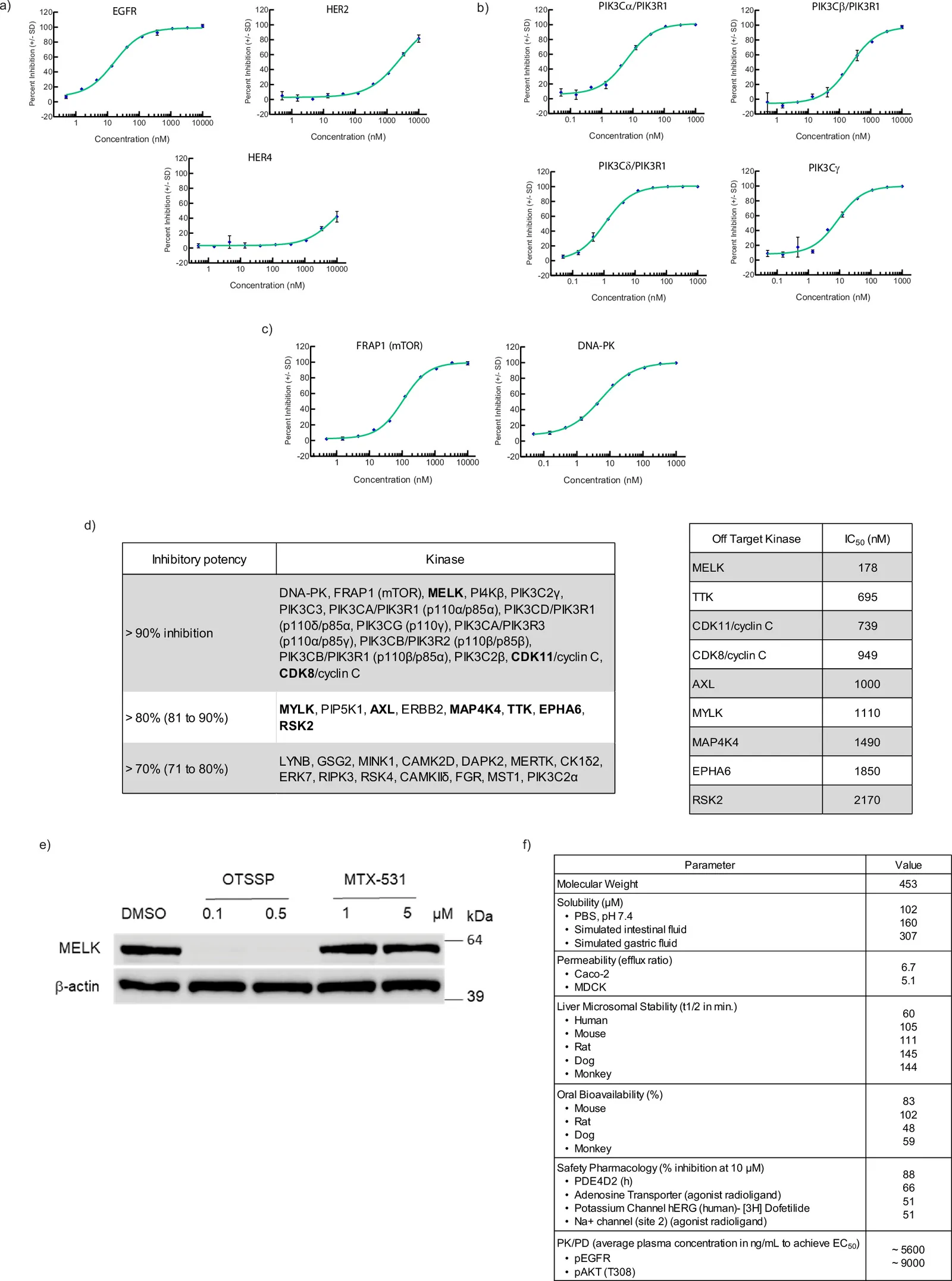
MTX-531 potently and selectively inhibits EGFR and PI3K family members. The biochemical potency of MTX-531 was evaluated against **a**, purified HER and **b**, purified PI3K family members. IC_50_ values were determined at a concentration of ATP corresponding to the K_m_ apparent for that kinase. Data are presented as mean ± SD and are representative of two individual experiments at varying concentrations of MTX-531 in duplicate. **c**, Inhibitory activity of MTX-531 against FRAP (mTOR) and DNA-PK was determined as described above. Data are presented as mean ± SD and are representative of two individual experiments carried out over a range of MTX-531 concentrations tested in duplicate. **d**, Broad kinome screening of MTX-531: the inhibitory potency of MTX-531 was determined against a panel of 482 protein and lipid kinases by carrying out single point testing at a final concentration of 10 µM MTX-531 in duplicate assays. The nine off-target kinases inhibited by >80% at 10 µM shown in bold font (upper panel) were re-tested in dose response assays at concentrations tested in duplicate to determine IC_50_ values (bottom panel). **e**, Immunoblot analysis of MELK expression in lysates from CAL-33 cells treated for 24 hours with the indicated concentrations of MTX-531 or the MELK inhibitor, OTSSP167. Data are representative of two individual experiments. **f**, Summary of drug properties of MTX-531. Source data

MTX-531 exhibits low-nanomolar potency against purified EGFR and PI3Kα with half-maximal inhibitory concentration (IC_50_) values of 15 nM and 6.4 nM, respectively (Table 1 and Extended Data Fig. 1a,b). The comparator molecules omipalisib and NVP-AEE788 are ~30-fold to 50-fold more potent than MTX-531 against PI3K and EGFR, respectively. Clinical development of both molecules was terminated, likely influenced by their toxicity profiles^24,25^. The strong potency of MTX-531 against multiple PI3K family members and mTOR reveals a biochemical profile distinctly different from clinically approved alpelisib (Piqray) and copanlisib (Aliqopa) (Table 1 and Extended Data Fig. 1b,c). Copanlisib and MTX-531 share a pan-PI3K inhibitory profile, whereas alpelisib is 1–3 logs more potent against PI3Kα compared to other PI3K isoforms. MTX-531 is only ~15-fold less potent against mTOR compared to PI3Kα, a feature distinguishing it from both alpelisib and copanlisib. Subsequent evaluation of kinome selectivity against a broad panel of >400 protein and lipid kinases^26^ revealed that MTX-531 is exquisitely selective for HER (human EGFR) and PI3K family members (Fig. 1e and Supplementary Table 3). At 10 µM, only nine protein kinases outside of the HER family were inhibited by >80%. Titration of MTX-531 against these nontargeted kinases revealed insignificant inhibition (IC_50_ ≥ 0.5 μM) against all but maternal embryonic leucine zipper kinase (MELK; IC_50_ 178 nM) (Extended Data Fig. 1d). Off-target inhibition of MELK is believed to be inconsequential because MTX-531 had no cellular effect on MELK at concentrations as high as 5 µM (Extended Data Fig. 1e). Screening against a large panel (*n* = 86) of largely nonkinase targets involved in clinical adverse drug reactions revealed only four proteins binding to MTX-531 with an IC_50_ < 10 μM (Extended Data Fig. 1f and Supplementary Table 4).

**Table 1:** Biochemical potency of MTX-531 and comparator PI3K and EGFR inhibitors against purified HER and PI3K family members

	MTX-531	Alpelisib	Copanlisib	Omipalisib	NVP-AEE788	Erlotinib
**HER family**	IC_50_ (nM)					
EGFR	14.7	>10,000	>10,000	>10,000	0.31	0.48
HER2	2,500	>10,000	>10,000	>10,000	26.1	192
HER4	>10,000	>10,000	>10,000	>10,000	221	159
**PI3K family**	IC_50_ (nM)					
PI3Kα	6.4	2.2	0.17	0.23	>10,000	>10,000
PI3Kβ	233	2,010	7.5	1.7	>10,000	>10,000
PI3Kγ	8.3	49.8	0.99	0.62	>10,000	>10,000
PI3Kδ	1.1	39.7	0.16	0.23	>10,000	>10,000
mTOR	105	2,940	18.8	2.4	>10,000	>10,000
DNA-PK	5.4	6,610	17.0	0.15	>10,000	>10,000

DNA-PK, DNA-dependent protein kinase.

**Fig. 2:**
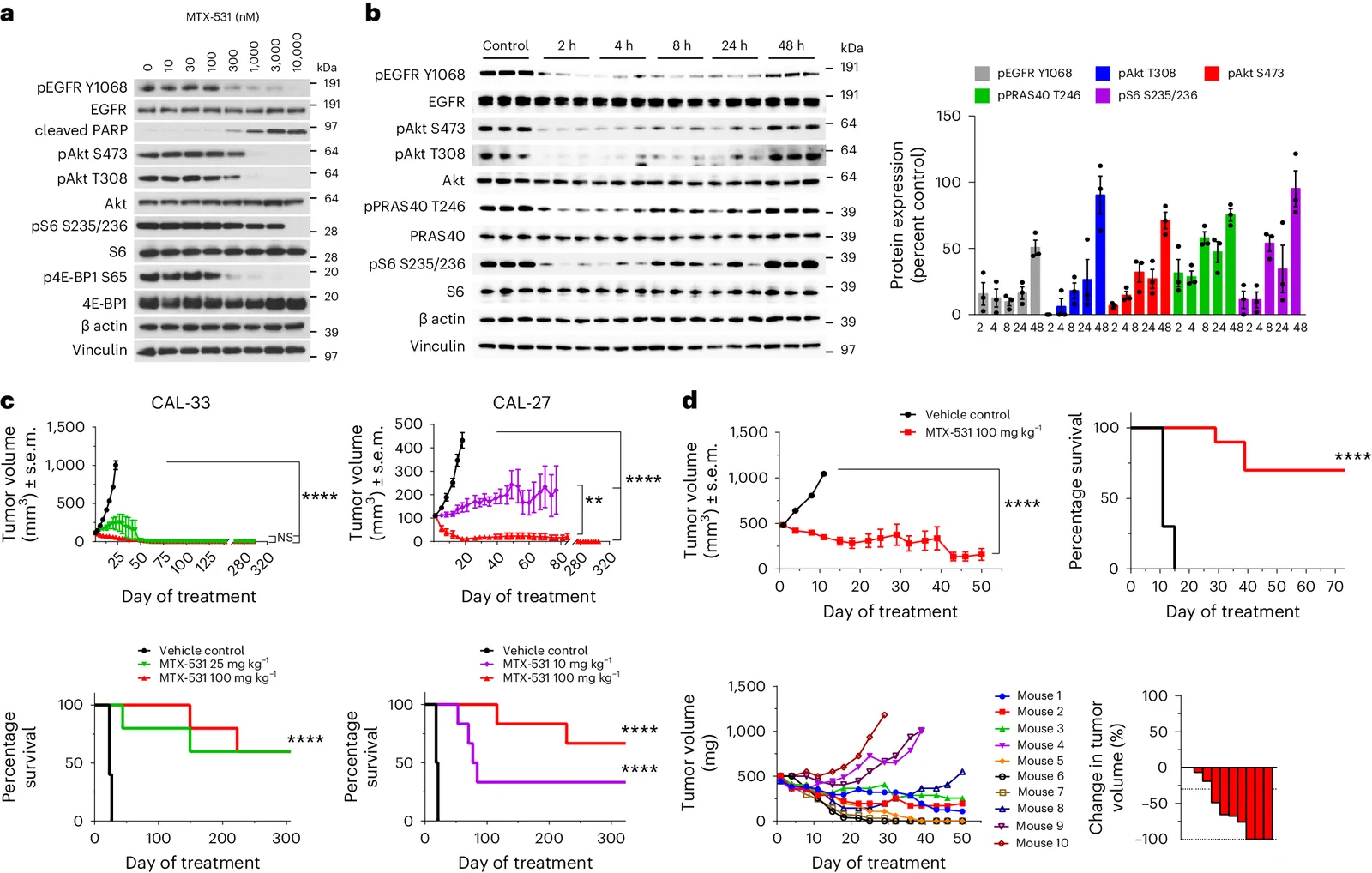
MTX-531 cotargets cellular EGFR and PI3K signaling in HNSCC CAL-33 and CAL-27 models. **a**, Immunoblot analyses of EGFR and PI3K–mTOR pathway expression in CAL-33 cells treated for 2 h with MTX-531 over a broad dose range. The images are representative of three repetitions of the experiment. **b**, Pharmacodynamic activity of MTX-531 in CAL-33 tumor-bearing mice treated with a single oral dose of 100 mg kg^−1^. Immunoblot analyses of tumors isolated at the indicated time points (*n* = 3 tumors per group) (left) were quantified by densitometry analyses of phosphorylated kinase expression (right). Data are representative of two individual experiments and are presented as the mean ± s.e.m. **c**, Antitumor efficacy of MTX-531 against CAL-33 (left; *n* = 5 mice per group) or CAL-27 (right; *n* = 6 mice per group) xenografts. MTX-531 was dosed daily by oral gavage at the indicated dosage for 134 days (CAL-33) or 145 days (CAL-27) except for the 100 mg kg^−1^ arm of the CAL-33 study where dosing stopped after 37 days to monitor the durability of complete responders. Top, data are shown as the mean tumor volume ± s.e.m. An unpaired two-sided *t*-test was carried out to determine statistical significance. Bottom, the effects of treatment on survival were quantitated by euthanizing individual mice when tumor burden reached an equivalent size (1,000 mm^3^ for CAL-33 and 500 mm^3^ for CAL-27). A one-way analysis of variance (ANOVA) comparison among all groups was carried out to determine statistical significance. **d**, Antitumor efficacy of MTX-531 against advanced-stage CAL-33 xenografts. Daily treatment with MTX-531 (100 mg kg^−1^ per os (PO)) was initiated when mean tumor volumes reached ~500 mm^3^ (*n* = 10 mice per group). Top left, data are shown as the mean tumor volume ± s.e.m. Top right, the effects of treatment on survival were quantified by euthanizing individual mice when the tumor burden reached 1,000 mm^3^. Statistical differences in survival between the vehicle-treated and the MTX-531-treated group were determined using the log-rank (Mantel–Cox) test. The best individual response of MTX-531-treated mice (bottom left) is depicted in a waterfall plot (bottom right). **P < 0.01 and ****P ≤ 0.0001. Source data

**Extended Data Fig. 2:**
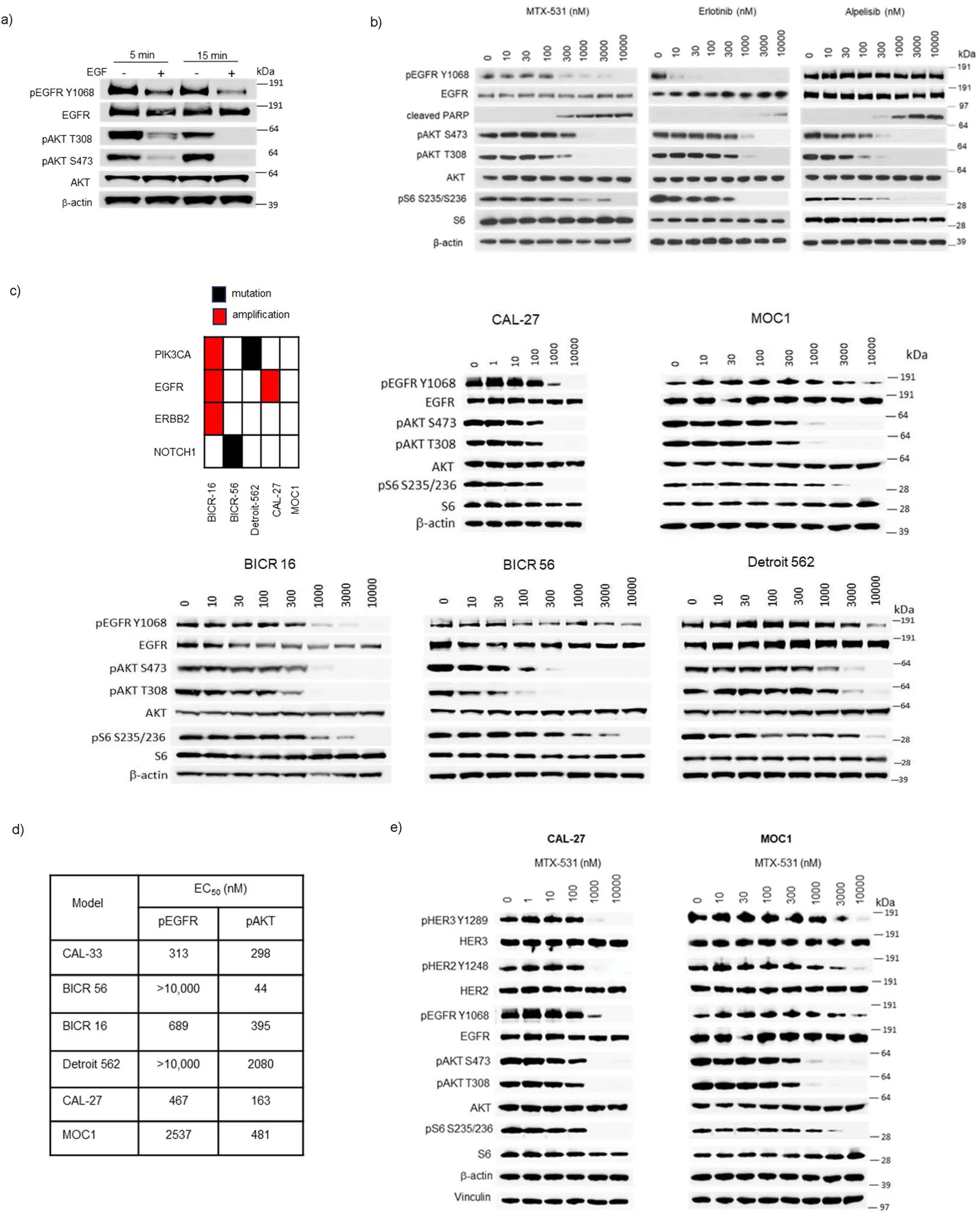
MTX-531 co-targets cellular EGFR and PI3K signaling in HNSCC models. **a**, Time-dependent inhibition of EGFR and PI3K in CAL-33 cells treated with 1 µM MTX-531. Data are representative of two individual experiments **b**, Comparative cellular potency of MTX-531, erlotinib and alpelisib in CAL-33 cells treated for 2 hours over a range of doses. Data are representative of two individual experiments **c**, Cellular activity of MTX-531 across a broad panel of HNSCC cell lines. EGFR and PI3K/mTOR pathway inhibition was evaluated by immunoblotting analyses in cells treated for 2 hours with varying concentrations of MTX-531. Data are representative of two individual experiments **d**, Comparative cellular potency of MTX-531 against cellular EGFR and PI3K signaling in a panel of HNSCC cell lines. Densitometry analysis was performed from immunoblotting carried out in panel 2c to determine EC_50_ values. Data are representative of two individual experiments **e**, Effect of MTX-531 on activated HER3 and EGFR expression in PI3K wild type cells. Immunoblotting analysis was carried out in CAL-27 and MOC1 cells treated for two hours with MTX-531. Data are representative of two individual experiments. Source data

Cellular evidence for MTX-531’s ability to cotarget EGFR and PI3K was generated in the CAL-33 HNSCC model known to possess a *PIK3CA* mutation (H1047R). After a 2-h treatment, MTX-531 demonstrated concentration-dependent inhibition of EGFR, PI3K and mTOR as measured by lower levels of phosphorylated (p)EGFR, protein kinase B (Akt) and eukaryotic translation initiation factor 4E-binding protein 1 (4E-BP1) (Fig. 2a). Balanced cellular inhibition of these targets is reflected in comparable IC_50_ values (~300 nM), demonstrating that MTX-531 is equipotent at inhibiting both EGFR and PI3K–mTOR signaling. Furthermore, significant induction of apoptosis as measured by expression of cleaved poly(ADP-ribose) polymerase (PARP) was observed when concentrations of MTX-531 approached 1 µM. A time-dependent increase in pathway inhibition was shown with shorter incubation periods (Extended Data Fig. 2a). MTX-531 at 1 µM resulted in maximal inhibition of pAkt_T308_ expression (97% inhibition) in CAL-33 cells within 15 min of addition of drug. Inhibition of pEGFR expression after 15 min was significant (65% reduction) but did not reach the degree of inhibition (90%) seen at 2 h in Fig. 2a. Single-target inhibition of EGFR or PI3K was evaluated in CAL-33 cells treated with erlotinib or alpelisib (Extended Data Fig. 2b). Consistent with its strong degree of activity in purified enzyme studies (Table 1), erlotinib was highly potent against pEGFR expression (half-maximal effective concentration (EC_50_) < 10 nM) requiring ~2-log higher concentrations to exert downstream effects on PI3K signaling. Induction of apoptosis seen in response to MTX-531 and alpelisib was not seen with erlotinib, consistent with the rapid onset of apoptosis known to accompany PI3K inhibition^27^. The cellular effects of MTX-531 on EGFR and PI3K signaling were further studied in a genomically diverse panel of HNSCC cell lines (Extended Data Fig. 2c). Equipotent inhibition of both pathways by MTX-531 was seen in models aberrantly expressing both targets, including BICR 16 cells that are devoid of a mutation or amplification of *PI3KCA* (Extended Data Fig. 2d). This finding likely relates to their expression of mutant Notch 1, which has been reported to confer vulnerability to PI3K–mTOR inhibition in HNSCC^28^.

Potent inhibition of PI3K signaling in the syngeneic mouse oral carcinoma 1 (MOC1) model provides cellular evidence that MTX-531 is as potent against wild-type PI3K as the mutant enzyme. Gutkind and colleagues have provided compelling support for the role of HER3 phosphorylation in driving PI3K–mTOR pathway signaling in wild-type *PIK3CA* HNSCC^29^. We evaluated MOC1 and CAL-27 cells, which are wild-type for PIK3CA, for the effects of MTX-531 on pHER3 expression (Extended Data Fig. 2e). pHER3 levels in the CAL-27 model, which shows amplification of EGFR, were significantly reduced in response to treatment with MTX-531, as were levels of pEGFR and pHER2. The effects of MTX-531 on the expression of activated HER family members were comparatively diminished in MOC1 cells, which have no known aberration in EGFR.

### MTX-531 monotherapy leads to regression of HNSCC xenografts

**Fig. 3:**
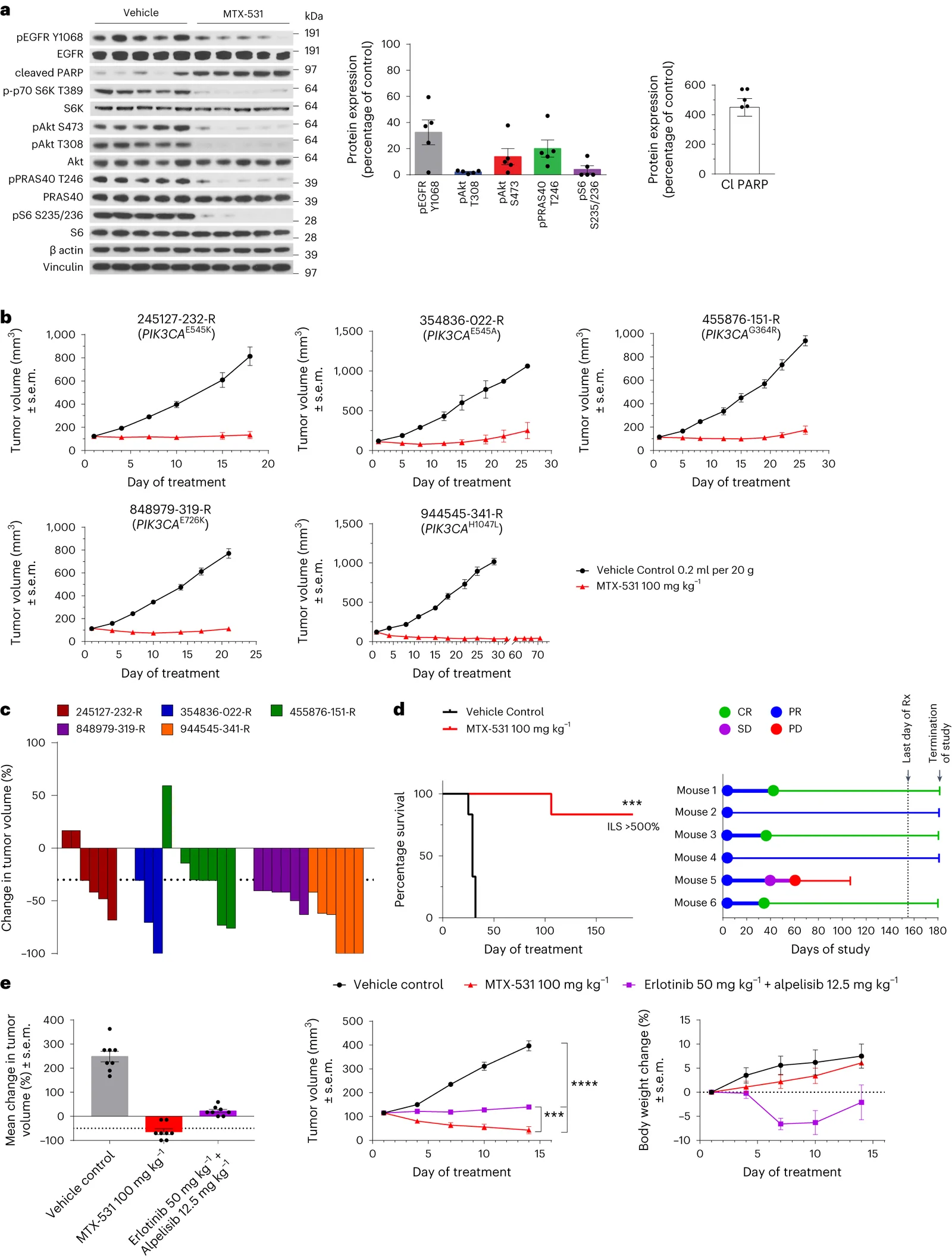
MTX-531 inhibits EGFR and PI3K–mTOR signaling and tumor growth in HNSCC PDX models. **a**, Pharmacodynamic modulation of EGFR and PI3K–mTOR pathway expression in NCI 848979-319-R xenografts 2 h after a single oral dose of 100 mg kg^−1^ MTX-531 (*n* = 5 mice per group) followed by immunoblot analysis and quantification by densitometry (right). Data are presented as the mean values ± s.e.m. and are representative of two individual experiments. **b**, Tumor growth inhibition after oral daily administration of 100 mg kg^−1^ MTX-531 to mice implanted with *PIK3CA*-mutant HNSCC PDX models NCI 245127-232-R (*n* = 6 mice per group), NCI 354836-022-R (*n* = 5 mice per group), NCI 455876-151-R (*n* = 8 mice per group), NCI 848979-319-R (*n* = 8 mice per group) and NCI 944545-341-R (*n* = 6 mice per group). Data are shown as the mean tumor volume ± s.e.m. Statistical differences in tumor growth rates for MTX-531 versus vehicle treatment were determined using a linear mixed model (Extended Data Fig. 4c). **c**, Waterfall plot of the best individual response of mice treated with MTX-531 in **b**. The percentage increase in tumor burden observed in the vehicle-treated mice ranged between 773% and 912% across models. **d**, Left, extension in survival of NCI 944545-341-R tumor-bearing mice (*n* = 6 mice per group). Mice were treated with MTX-531 (100 mg kg^−1^ PO) daily for 155 days or until tumor burden reached 1,000 mm^3^. Statistical differences in survival between the two arms was determined using the log-rank (Mantel–Cox) test. Right, plot depicting the duration of treatment required to elicit objective responses in individual mice. **e**, Antitumor efficacy of MTX-531 versus combination of erlotinib and alpelisib against NCI 944545-341-R xenografts. Mice (*n* = 8 mice per group) were treated daily for 14 days at the indicated doses. Data are shown as the mean change in tumor volume ± s.e.m. (left) and the mean change in tumor volume over time (middle). Statistical significance was determined by a one-way ANOVA comparison among all groups. Right, the effects of treatment on body weight change ± s.e.m. *P ≤ 0.05, **P ≤ 0.01, ***P ≤ 0.001 and ****P ≤ 0.0001. Source data

**Extended Data Fig. 3:**
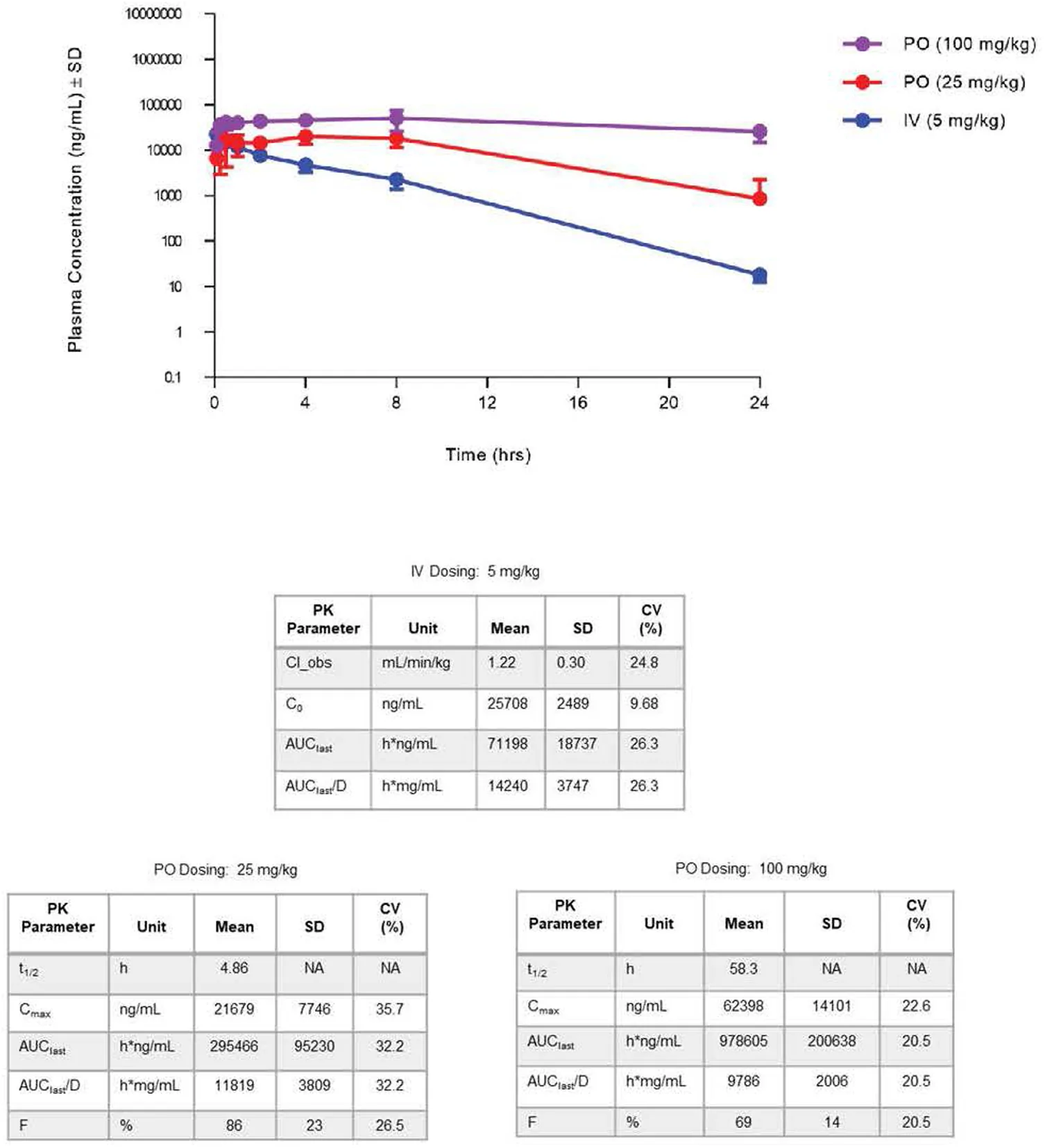
Pharmacokinetic profiling of MTX-531. Pharmacokinetic analysis of MTX-531 in mice. Mice received a single treatment of MTX-531 at 5 mg/kg intravenously (IV) or 25 and 100 mg/kg by oral gavage (PO), n = 3 mice/treatment arm/time point. Blood was collected at the indicated timepoints and the plasma concentration of MTX-531 was determined using liquid chromatography-mass spectrometry. Data are presented as mean ± SD and are representative of three individual experiments. Source data

**Extended Data Fig. 4:**
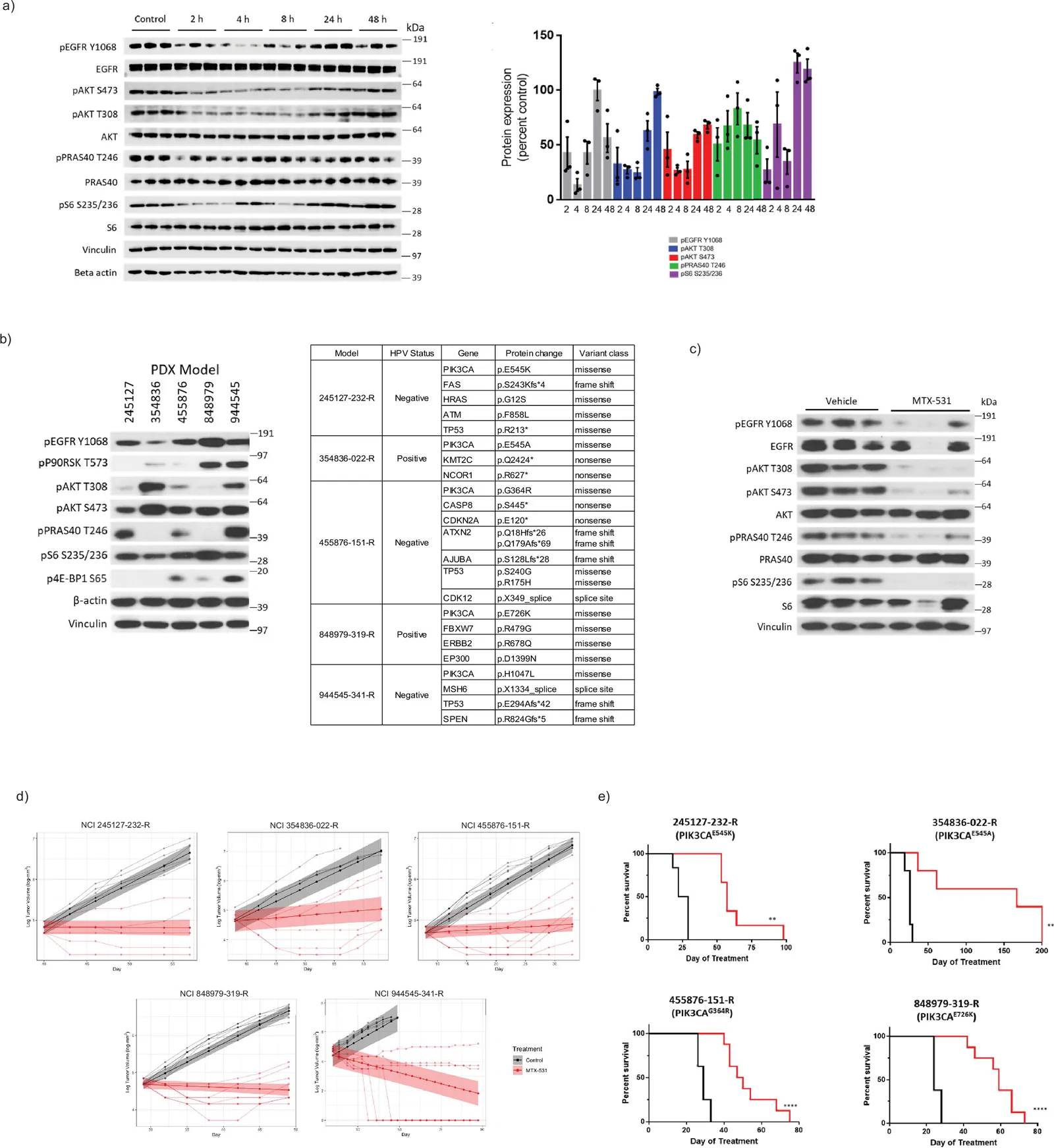
Pharmacodynamic and efficacy evaluation of MTX-531 in preclinical HNSCC models. **a**, Immunoblotting analysis of subcutaneous CAL-33 tumors excised from mice (n = 3 tumors/group) treated with a single oral dose of MTX-531 at 25 mg/kg. Tumors were harvested at the indicated time point (left panel). Quantification of phosphorylated kinase expression of the individual bands was carried out by densitometry analysis as shown in the panel on the right. Data are presented as mean values ± SEM and are representative of two independent experiments. **b**, Baseline expression analysis of EGFR and PI3K/mTOR signaling in five HNSCC PDX models selected for efficacy evaluation of MTX-531. Equivalent amounts of protein from each model were added to each lane for comparative analysis. HPV status and genomic alterations of these models, obtained from the NCI PDMR database, are shown on the right. Representative data are shown from three independent experiments. **c**, Pharmacodynamic modulation of EGFR and PI3K/mTOR pathway expression in subcutaneous NCI 944545-341-R patient-derived xenografts. Mice were treated with MTX-531 (100 mg/kg PO) and tumors were excised at 2 hours after the fifth dose (n = 3 tumors/group). Representative data are shown from two independent experiments. **d**, Statistical evaluation of single agent activity of MTX-531 as determined by a Linear Mixed Model (LMM). Statistical significance of differences in HNSCC PDX growth in response to treatment with MTX-531 was determined for the efficacy studies summarized in Fig. 3. Spaghetti plots are shown for each model. The expected log mass trajectories for vehicle versus MTX-531 treatment are depicted in the shaded areas, which denote the 95% point-wise confidence intervals from the LMM fitting. **e**, Impact of MTX-531 on survival of HNSCC PDX-bearing mice. Kaplan-Meier survival plots are shown for the panel of PIK3CA mutant HNSCC PDX models evaluated in this study. Mice were dosed daily with MTX-531 (100 mg/kg PO) until individual tumors reached 1000 mm^3^ (n = 5-8 mice/group). Survival curves were compared using the Log-rank (Mantel-Cox) test (** = p-value ≤ 0.01, **** = p-value ≤ 0.0001). Source data

MTX-531 exhibits a favorable drug-like profile, supporting its advanced preclinical development, and exhibits high (~80%) oral bioavailability in mice, rendering it conducive to oral dosing studies (Extended Data Fig. 3). An early single-agent in vivo evaluation of MTX-531 focused on a determination of its therapeutic activity against high-passage HNSCC xenografts because both EGFR and PI3K have prominent roles in the progression of this disease. EGFR is overexpressed in roughly 90% of HNSCCs, while widespread activation of PI3K–mTOR signaling also occurs in >80% of cases^7,30–32^. Pharmacodynamic evaluation of CAL-33 tumors excised after a single oral dose of MTX-531 showed time-dependent suppression of EGFR and PI3K–mTOR pathway signaling (Fig. 2b). A >50% reduction in levels of pEGFR and pAkt was maintained for 24 h in mice dosed at 100 mg kg^−1^. Control levels of pAkt_T308_ and pS6 returned by 48 h, while pEGFR expression remained significantly suppressed. A reduced dose of 25 mg kg^−1^ also resulted in strong pharmacodynamic activity with >50% inhibition after a single dose but was not sustainable for 24 h (Extended Data Fig. 4a). Consistent with its pharmacodynamic activity, MTX-531 was highly efficacious against CAL-33 and CAL-27 xenografts, resulting in 33–100% incidence of complete responses across a broad dose range (Fig. 2c). Dosing occurred daily until individual tumors reached the same size to facilitate comparative survival analysis (500 and 1,000 mm^3^ for CAL-27 and CAL-33, respectively). Studies were terminated >300 days after tumor implantation at which time the median increase in survival exceeded 1,000% with >50% of the mice remaining tumor free in all groups dosed at 25 mg kg^−1^ or greater. MTX-531 was also efficacious against CAL-33 tumors reaching an advanced stage (~500 mm^3^) before initiation of treatment (Fig. 2d). Daily treatment for 6 weeks led to >355% improvement in median survival, with objective responses seen in seven of ten mice, including three complete regressions. MTX-531 was further evaluated against a heterogeneous panel of *PIK3CA*-mutant HNSCC PDX models obtained from the National Cancer Institute (NCI) Patient-Derived Models Repository (PDMR). Baseline pathway expression analysis showed consistently high levels of pEGFR in 80% of the models and highly variable expression of kinases in the PI3K–mTOR pathway (Extended Data Fig. 4b). Representative pharmacodynamic data generated for these models showed that MTX-531 completely blocked activation of Akt and S6 within 2 h of dosing (Fig. 3a and Extended Data Fig. 4c). MTX-531 was highly efficacious against the PDX panel, resulting in regressions in every model evaluated (Fig. 3b,c and Extended Data Fig. 4d). The *HPV*^−^ model 944545-341-R was an exceptional responder, as reflected by a 50% complete response rate and a >500% increase in median survival (Fig. 3d). Regressions of 944545-341-R tumors were durable as three of six mice remained tumor free when the study was terminated after 172 days of dosing. The collective data across all five PDX models showed a mean overall response rate of 66% and an increase in median survival ranging from 62% to 542% (Extended Data Fig. 4e).

**Extended Data Fig. 5:**
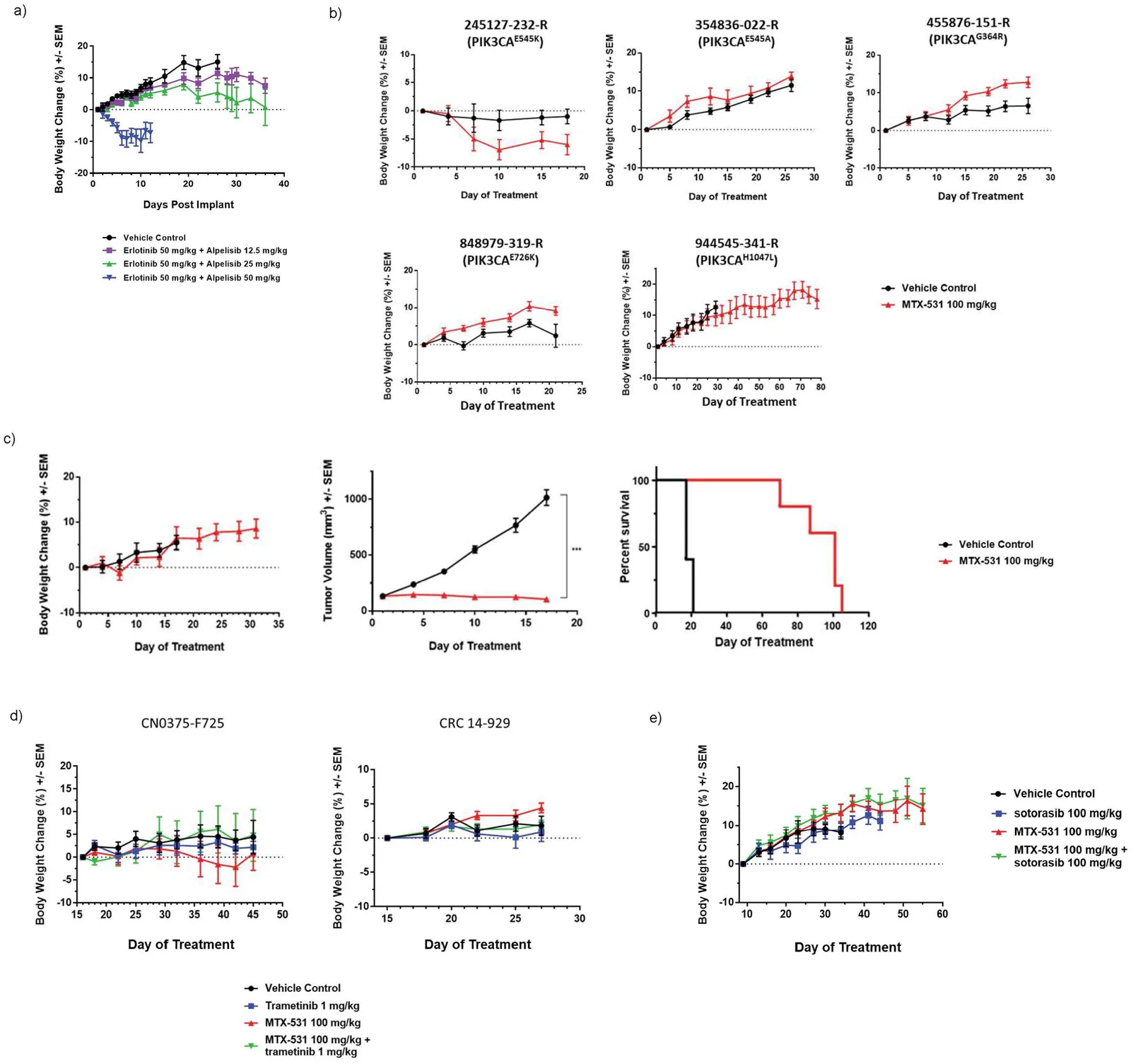
Body weight changes in mice treated with MTX-531 alone or in combination. **a**, Selection of a combination dosage of erlotinib and alpelisib. Mice were treated with the combination of erlotinib and alpelisib daily via oral gavage for 5 weeks (n = 8 mice/group). The daily dose of erlotinib was held constant at 50 mg/kg and the dose of alpelisib was varied (12.5, 25 and 50 mg/kg). Body weight loss of ≥ 20% and/or ≥ 10% treatment-related deaths in a group are considered unacceptable toxicity parameters. Three deaths occurred in each of the two highest dose combination groups. **b**, Body weight of subcutaneous HNSCC PDX-bearing mice in response to daily oral treatment with MTX-531 at 100 mg/kg. Body weight was monitored throughout the efficacy study shown in Fig. 3b. **c**, Response of PIK3CA mutant HNSCC NCI 848979-319-R PDX xenografts to daily oral dosing of 150 mg/kg MTX-531. Data are presented as mean values ± SEM. Dosing occurred until individual tumors reached 1000 mm^3^ to facilitate comparative survival analysis (n = 5 mice/group). A comparison of survival curves was performed using the Log-rank (Mantel-Cox) test (** = p-value ≤ 0.01, *** = p-value ≤ 0.001). **d**, Body weight of mice bearing CRC PDX models CN0375-F725 (5 mice/group) and UM-CRC 14-929 (5 mice/group) in response to daily treatment with MTX-531 (100 mg/kg PO) in combination with trametinib (1 mg/kg PO). Data are presented as mean values ± SEM. **e**, Body weight of mice bearing CRC B8324 patient-derived xenografts (5 mice/group) in response to daily oral treatment with MTX-531 (100 mg/kg) in combination with sotorasib (100 mg/kg). Data are presented as mean values ± SEM. For all panels, body weights were recorded 2-3 times per week. Source data

A head-to-head evaluation of MTX-531 versus a combination of erlotinib and alpelisib was carried out in the 944545-341-R PDX model (Fig. 3e). MTX-531 monotherapy resulted in a mean tumor regression of 70%, whereas no regressions were observed in the erlotinib–alpelisib combination arm. Higher doses of alpelisib were not tolerated in the combination regimen (Extended Data Fig. 5a). MTX-531, administered daily at 100 mg kg^−1^, was well tolerated throughout all HNSCC PDX studies. Mice continued to gain weight for the duration of dosing, exceeding 10 weeks in one study (Extended Data Fig. 5b). MTX-531 was also tolerated at 150 mg kg^−1^, leading to a further boost in efficacy against 848979-319-R xenografts and a threefold improvement in median survival compared to mice treated at 100 mg kg^−1^ (Extended Data Fig. 5c).

### MTX-531 potentiates MEK inhibition in CRC

**Fig. 4:**
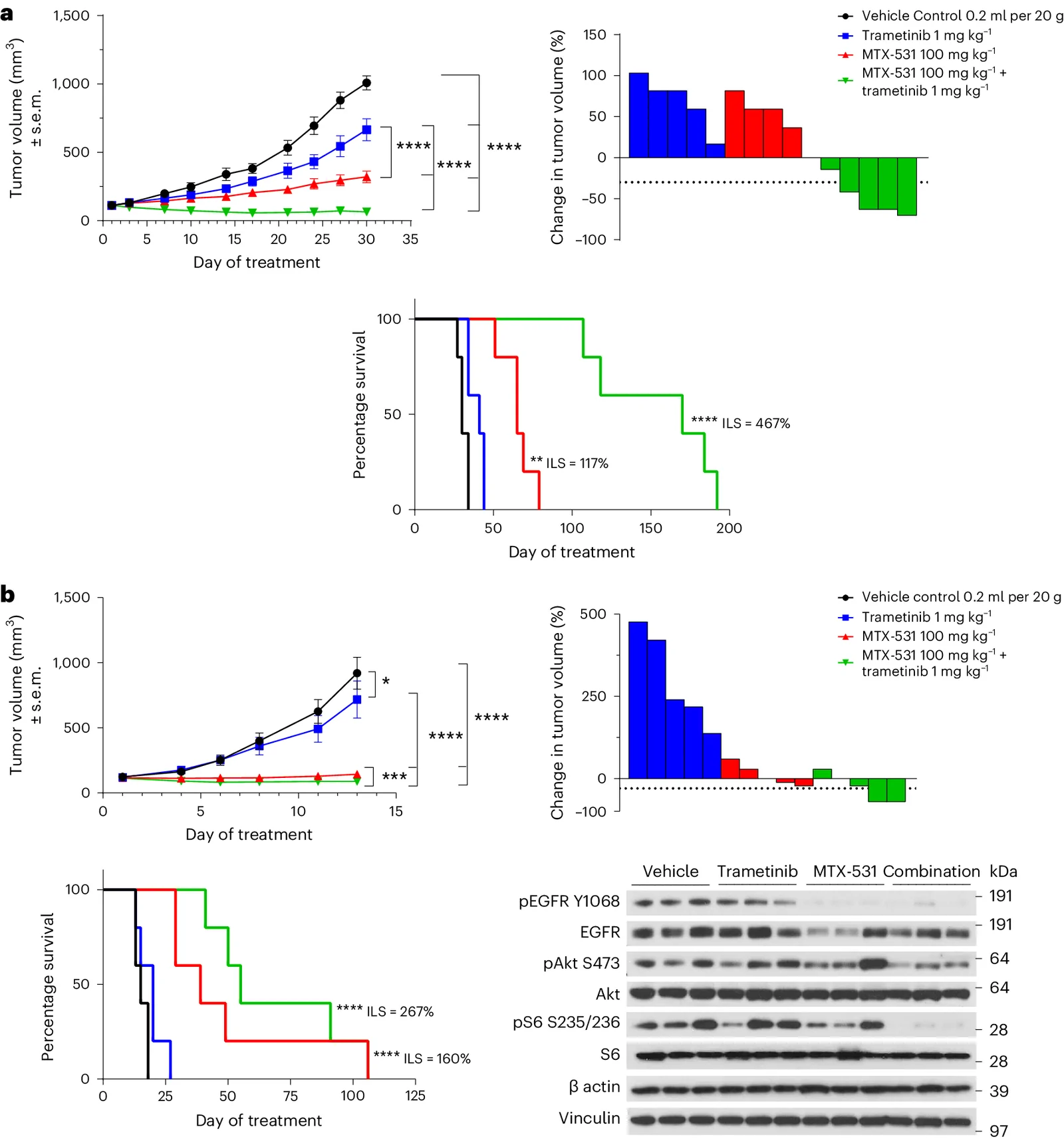
Combination of MTX-531 with the MEK inhibitor trametinib leads to regressions in *BRAF*-mutant and *KRAS*-mutant CRC models. Tumor growth inhibition after treatment with MTX-531, trametinib or their combination administered orally to mice bearing KRAS-A146T NCI CN0375-F725 PDX tumors (**a**; *n* = 5 mice per group) or BRAF-V600E UM-CRC 14-929 PDX tumors (**b**; *n* = 5 mice per group). Top left, data are shown as the mean tumor volume ± s.e.m. Top right, the best antitumor response seen in individual animals from each group is shown as a waterfall plot. The percentage increase in tumor burden observed in the vehicle-treated mice during these studies was 887% (NCI CN0375-F725) or 844% (UM-CRC 14-929). Bottom, the extension in survival conferred by each of the single agents and their combination. Effects of treatment on survival were quantified by euthanizing individual mice when the tumor burden reached 1000 mm^3^. Increase in lifespan (ILS) was determined from comparative survival of median animals in the treated versus control groups. A linear mixed model fit was used to determine the statistical significance of tumor growth rate differences and a Wald test was used for a significance test between the different groups. *P ≤ 0.05, ***P ≤ 0.001 and ****P ≤ 0.0001. Bottom right, pharmacodynamic modulation of EGFR and PI3K–mTOR pathway expression was evaluated in UM-CRC 14-929 xenografts. Mice were treated for 5 days at the same doses as studied in the efficacy experiment. Tumors were excised 2 h after the fifth treatment, followed by immunoblot analysis (*n* = 3 tumors per group). Data are representative of two individual experiments. Source data

Triple combinations directed against EGFR (cetuximab), PI3K (alpelisib) and downstream MAPK pathway targets (encorafenib or trametinib), while scientifically sound, have proven challenging in part because of poor tolerability^19,20^. Because MTX-531 targets two of the three critical signaling nodes targeted in these trials, we investigated the therapeutic impact of combining it with the MEK inhibitor trametinib to treat *KRAS*-mutant and *BRAF*-mutant CRC PDX models. NCI CN0375-F725 (KRAS-A136T) xenografts did not respond to single-agent treatment with trametinib or MTX-531 (Fig. 4a). However, an 80% objective response rate was achieved in the combination arm, where animals exhibited a 467% median increase in survival.

The combination of MTX-531 and trametinib was also efficacious in a *BRAF*^V600E^-mutant CRC PDX model (UM-CRC 14-929) (Fig. 3b). Whereas trametinib was inactive, MTX-531 monotherapy led to tumor stasis in most animals. The combination of MTX-531 and trametinib led to a further improvement in efficacy, reflected by a 40% partial response rate. A pharmacodynamic assessment of kinase expression in excised tumors from the combination arm showed a striking 97% reduction in pS6 levels consistent with improved efficacy over single-agent performance (Fig. 4b). Addition of 1 mg kg^−1^ trametinib to the daily dosing regimen of MTX-531 adopted in the monotherapy studies (100 mg kg^−1^) was well tolerated and did not lead to body weight loss (Extended Data Fig. 5d).

### MTX-531 is synergistic with KRAS-G12C inhibition

**Fig. 5:**
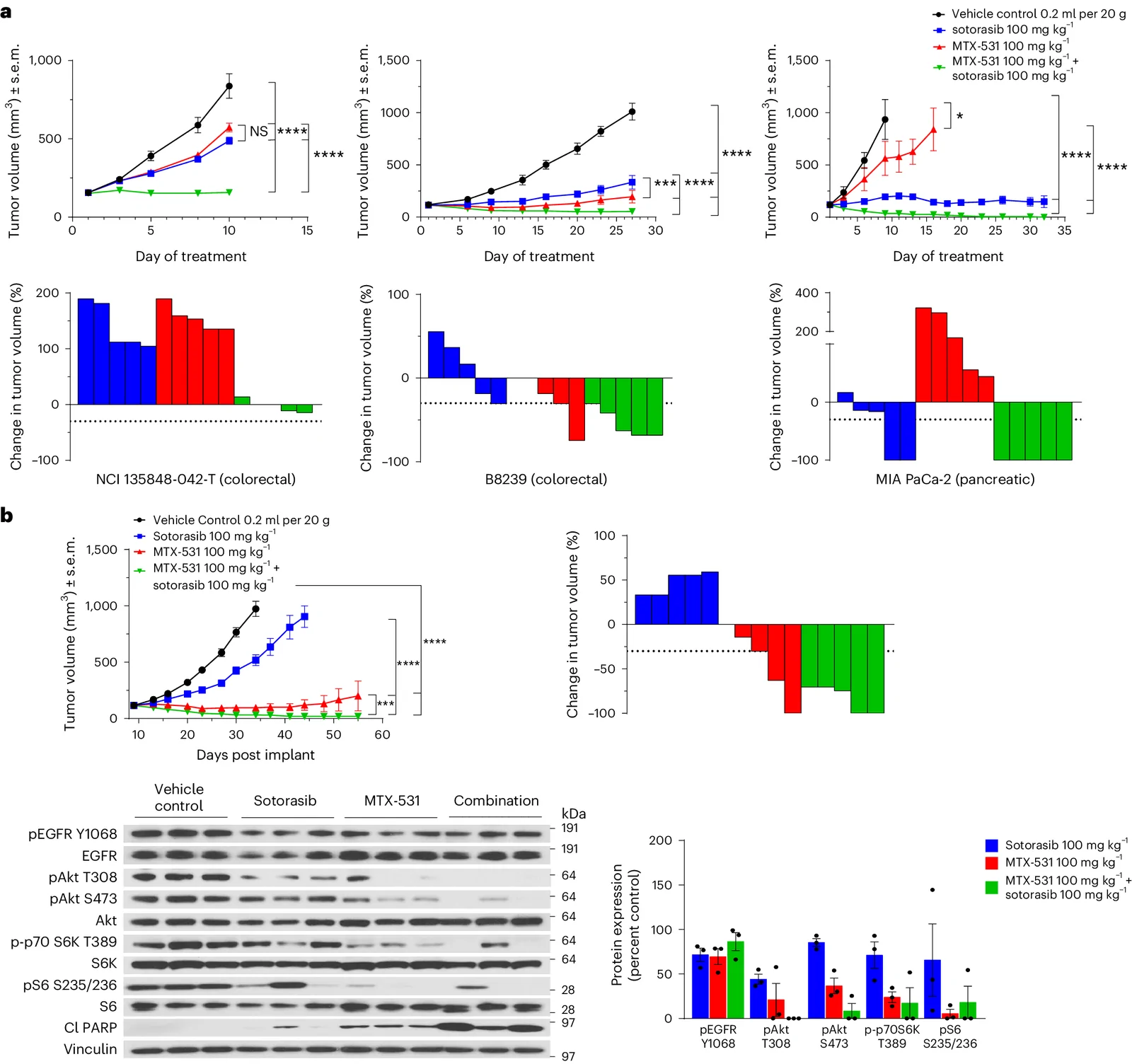
The combination of MTX-531 and sotorasib leads to regressions of *KRAS*^G12C^-mutant CRC and pancreatic tumors. **a**, Tumor growth inhibition was evaluated in response to treatment with MTX-531, sotorasib or their combination administered orally to mice bearing *KRAS*^G12C^-mutant xenografts. Studies were carried out in CRC PDX models NCI 135848-042-T (left) and B8239 (middle) and the pancreatic MIA PaCa-2 model (right) (*n* = 5 mice per group). Top, data are shown as the mean tumor volume ± s.e.m. Bottom, the best antitumor response seen in individual animals from each group is shown as a waterfall plot. The percentage increase in tumor burden observed in vehicle-treated mice was 434% (NCI 135848-042-T), 838% (B8239) and 970% (MIA PaCa-2). A linear mixed model fit was used to determine the statistical significance of tumor growth rate differences and a Wald test was used to determine significance between groups. *P ≤ 0.05, ***P ≤ 0.001 and ****P ≤ 0.0001). **b**, Top, therapeutic response of B8324 xenografts to treatment with MTX-531, sotorasib or their combination (*n* = 5 mice per group). B8324 is a *KRAS*^G12C^-mutant CRC PDX model established after progression during clinical treatment with the combination of sotorasib and panitumumab. Top left, data are shown as the mean tumor volume ± s.e.m. Top right, the best antitumor response seen in individual animals from each group is shown as a waterfall plot. The percentage increase in tumor burden observed in vehicle-treated mice was 817%. A linear mixed model fit was used to determine the statistical significance of tumor growth rate differences and a Wald test was used to determine significance between different groups. ***P ≤ 0.001 and ****P ≤ 0.0001. Bottom left, pharmacodynamic modulation of EGFR and PI3K–mTOR pathway expression in B8324 xenografts. Tumors were excised 2 h after treatment with a single oral dose of 100 mg kg^−1^ MTX-531, 100 mg kg^−1^ sotorasib or their combination, followed by immunoblot analysis (*n* = 3 tumors per group) and quantification of kinase expression by densitometry. Bottom right, representative data are presented as the mean ± s.e.m. of two individual experiments. Source data

Agents directly targeting KRAS-G12C have limited activity against *KRAS*^G12C^-mutant CRC in part because of EGFR-mediated reactivation of extracellular signal-regulated kinase (ERK) signaling, leading to combination trials with agents directed against KRAS-G12C and EGFR (ref. ^33^). However, secondary resistance mechanisms driven by other receptor tyrosine kinases (RTKs) or upregulation of mTOR signaling limit the durability of response to this combination strategy^34,35^. On the basis of its EGFR–PI3K–mTOR-inhibitory profile, we anticipated that MTX-531 would prove efficacious in combination with KRAS-G12C inhibitors. This hypothesis is supported by data generated from the combination of the KRAS-G12C inhibitor sotorasib with MTX-531 in mice bearing *KRAS*^G12C^-mutant CRC or pancreatic tumors. Dosing of the NCI 135848-042-T CRC xenografts, which additionally harbor mutations in *mTOR* (S2215F) and *ERBB2* (S310F), was curtailed to 10 days owing to >10% body weight loss in control animals. Nonetheless, tumor stasis was observed in the combination arm in contrast to inactivity in response to either single agent (Fig. 5a, left). B8239 CRC xenografts, which are also mutated in *PIK3CA* (H1047R) were modestly sensitive to sotorasib alone and comparatively more responsive to the single agent MTX-531, which elicited a 40% objective response rate (Fig. 5a, middle). However, treatment of B8239 tumor-bearing mice with the combination of sotorasib and MTX-531 led to a 100% incidence of partial regressions. The combination of sotorasib and MTX-531 was most efficacious against MIA PaCa-2 xenografts, where all mice showed complete regressions, surpassing the 40% incidence of complete regressions seen with the single agent sotorasib (Fig. 5a, right).

MTX-531 was further evaluated in B8324, a CRC *KRAS*^G12C^-mutant *PIK3CA*^E542K^-mutant PDX model established from a patient after progression on a combination regimen of sotorasib and panitumumab. B8324 xenografts were refractory to sotorasib treatment alone, consistent with clinical progression, yet sensitive to a single-agent treatment with MTX-531, as reflected by a 60% objective response rate (Fig. 5b). The combination of MTX-531 and sotorasib was exceptionally well tolerated (Extended Data Fig. 5e) and led to a 100% incidence of regressions and complete regressions in 40% of the group. Tumors excised from the combination arm showed a significant increase in apoptosis, as measured by increased expression of cleaved PARP, and strong suppression of PI3K–mTOR signaling, reflected by >90% and >80% inhibition of pAkt and pp70S6K expression, respectively (Fig. 5b, right). In contrast, MTX-531 resulted in a modest 14% reduction in pEGFR expression in this *KRAS*–*PIK3CA*-mutant model.

### MTX-531 does not lead to hyperglycemia in mice

**Fig. 6:**
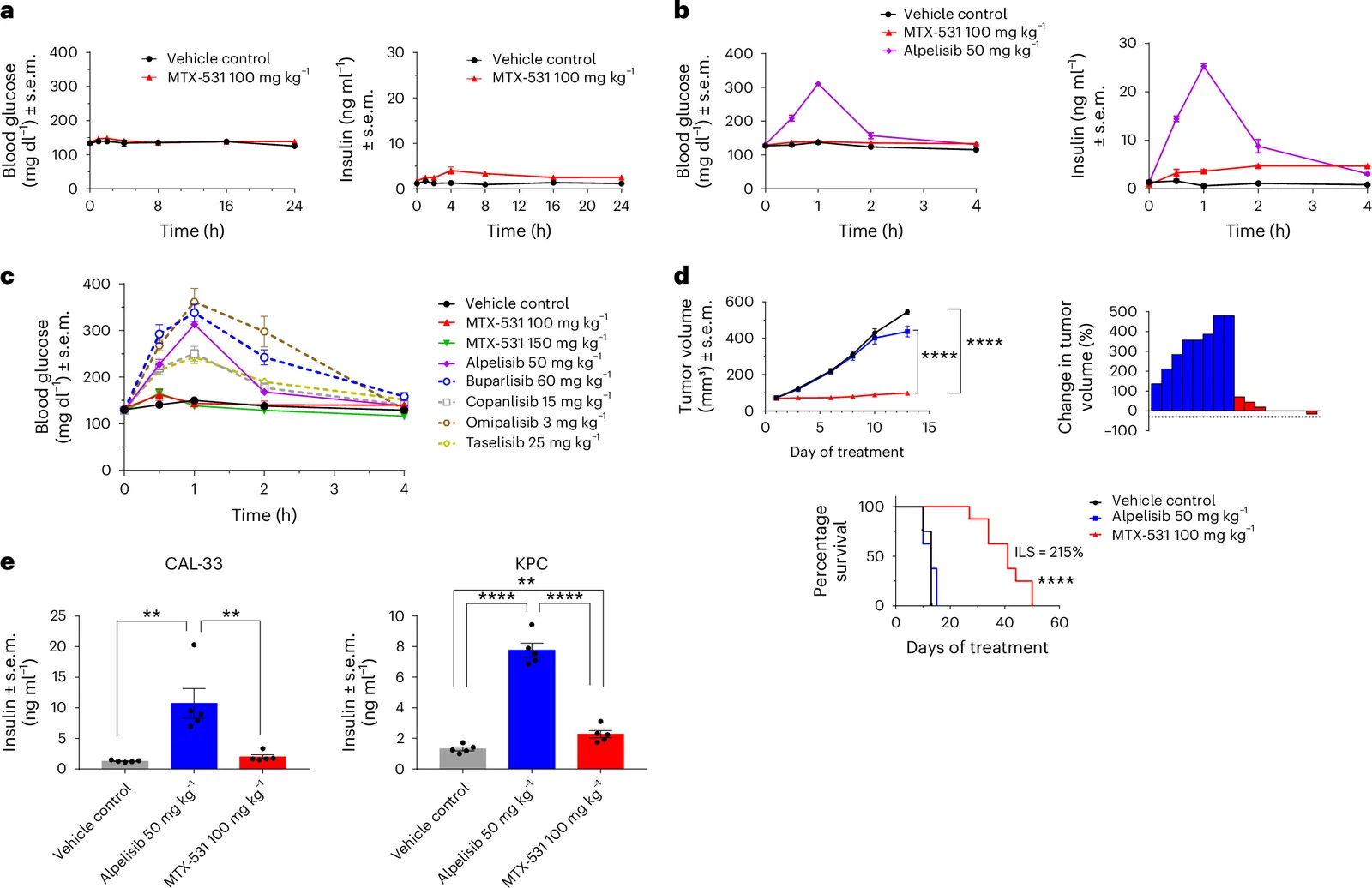
MTX-531 does not lead to hyperglycemia at therapeutic dose levels. **a**, Mice (*n* = 5 mice per group) were treated with a single oral dose of MTX-531 (100 mg kg^−1^), followed by measurement of blood glucose and plasma insulin levels over 24 h. **b**, Right, MTX-531 (100 mg kg^−1^) and alpelisib (50 mg kg^−1^) were compared for their effects on blood glucose and plasma insulin levels in mice treated with a single oral dose (*n* = 6 mice per group). Data are shown as the mean ± s.e.m. Blood glucose data are representative of four individual experiments. Insulin data are representative of three individual experiments. **c**, Blood glucose levels in mice treated with a panel of PI3K inhibitors (*n* = 8 mice per group for vehicle, MTX-531, alpelisib or buparlisib; *n* = 5 mice per group for copanlisib and taselisib; *n* = 3 mice per group for omipalisib). Data are representative of two individual experiments. **d**, Top left, comparative efficacy of MTX-531 versus alpelisib against subcutaneous KPC tumors. Data are shown as the mean tumor volume ± s.e.m. (*n* = 8 mice per group). The statistical significance of differences in tumor growth was determined using a linear mixed model. ****P ≤ 0.0001. Top right, the best antitumor response of individual animals from each group is shown as a waterfall plot. The percentage increase in tumor burden in the control group was 679%. Bottom, extension of survival of KPC-implanted mice treated daily with MTX-531 or alpelisib. Mice were treated daily for 65 days or until tumor burden reached 500 mm^3^. Statistical differences in survival between the vehicle-treated and MTX-531-treated groups was determined using the log-rank (Mantel–Cox) test. ****P ≤ 0.0001. **e**, Mean ± s.e.m. insulin concentrations in tumors excised from mice treated orally for 5 days with alpelisib (50 mg kg^−1^) or MTX-531 (100 mg kg^−1^) (*n* = 5 tumors per group). Data are representative of two individual experiments. Statistical differences in insulin levels between treatment groups were determined using an unpaired two-sided *t*-test (CAL-33: vehicle versus alpelisib, *P* = 0.0047; vehicle versus MTX-531, not significant; alpelisib versus MTX-531, *P* = 0.0075; KPC: vehicle versus alpelisib, *P* < 0.0001; vehicle versus MTX-531, *P* = 0.0065; alpelisib versus MTX-531, *P* < 0.0001). Source data

**Extended Data Fig. 6:**
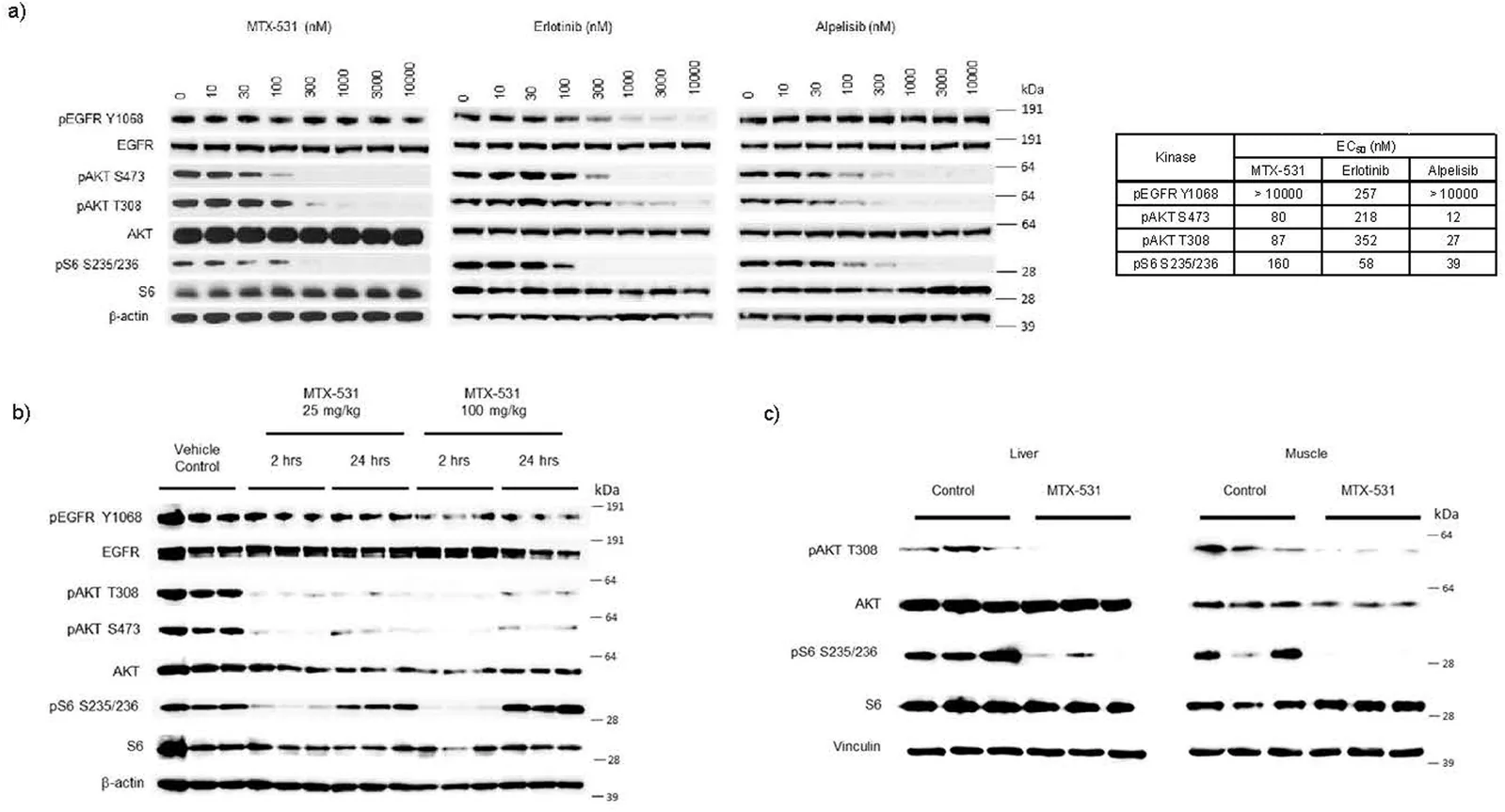
Inhibition of PI3K/mTOR pathway signaling in KPC tumors and normal tissues in response to MTX-531. **a**, Comparative cellular potency of MTX-531, erlotinib and alpelisib against EGFR and PI3K signaling in KPC cells. Cells were treated with the indicated drug for 2 hours across a broad dose range followed by immunoblot analysis. Densitometry was carried out to determine comparative EC_50_ values (right panel). Data are representative of two independent experiments. **b**, MTX-531 inhibits PI3K/mTOR signaling in KPC tumors. Pharmacodynamic modulation of PI3K/mTOR signaling in KPC tumors excised from mice (n = 3 tumors/group) treated with a single oral dose of MTX-531. Tumors were harvested at the indicated time point followed by immunoblot analysis. **c**, Evaluation of PI3K/mTOR pathway expression in liver and gastrocnemius (muscle) tissue excised from mice (n = 3 samples per tissue type) treated with a single oral dose of MTX-531 at 100 mg/kg. Tissues were harvested at two hours followed by immunoblot analysis. Representative data are shown from two independent experiments. Source data

Hyperglycemia is the most common on-target side effect of PI3K inhibitors because of the central role of PI3Kα in insulin signaling. Because MTX-531 is a pan-PI3K inhibitor, the observation that mice dosed at therapeutic levels did not show a significant rise in blood glucose levels was unexpected (Fig. 6a). Consistent with the absence of hyperglycemia, MTX-531 treatment also had no effect on blood insulin levels, unlike alpelisib, which elicited a significant rise in both plasma glucose and insulin levels (Fig. 6b). This result was confirmed upon testing an expanded panel of PI3K inhibitors including both clinically approved and failed agents (Fig. 6c). An escalation in dose of MTX-531 to 150 mg kg^−1^ also failed to induce hyperglycemia. Cantley and colleagues reported a role for systemic glucose–insulin feedback in mediating the reactivation of PI3K signaling in tumors treated with a PI3K inhibitor^36^. They showed that mice bearing syngeneic *Kras*;*Tp53*;Pdx-Cre (KPC) pancreatic tumors failed to respond to alpelisib unless placed on a ketogenic diet. When we directly compared the antitumor activity of MTX-531 to alpelisib in the KPC model, both molecules inhibited PI3K signaling in the 10–100 nM range in cultured cells, with alpelisib being ~5-fold more potent (Extended Data Fig. 6a). However, in KPC tumor-bearing mice, striking differences were observed in their activity. All control and alpelisib-treated mice were euthanized before 2 weeks of dosing could be completed, reflecting the highly aggressive nature of this tumor model (Fig. 6d). In contrast, tumor stasis was observed in MTX-531-treated mice. The impact of MTX-531 on median survival (215% increase) was comparable to that shown for mice treated with alpelisib and placed on a ketogenic diet^36^. Furthermore, insulin levels in CAL-33 and KPC tumors were significantly elevated in response to alplelisib but not MTX-531, consistent with differences in the circulating levels of insulin (Fig. 6e). Collectively, these studies suggest that MTX-531 may be less prone to insulin-mediated feedback mechanisms that compromise the antitumor activity of PI3K inhibitors. An evaluation of pAkt expression in KPC tumors and normal tissues involved in maintaining glucose homeostasis (namely, liver and skeletal muscle) confirmed that MTX-531 inhibited PI3K in both tumors and normal tissues within 2 h of dosing (Extended Data Fig. 6b,c).

### Peroxisome proliferator-activated receptor-γ (PPARγ) agonism is a unique feature of MTX-531

**Fig. 7:**
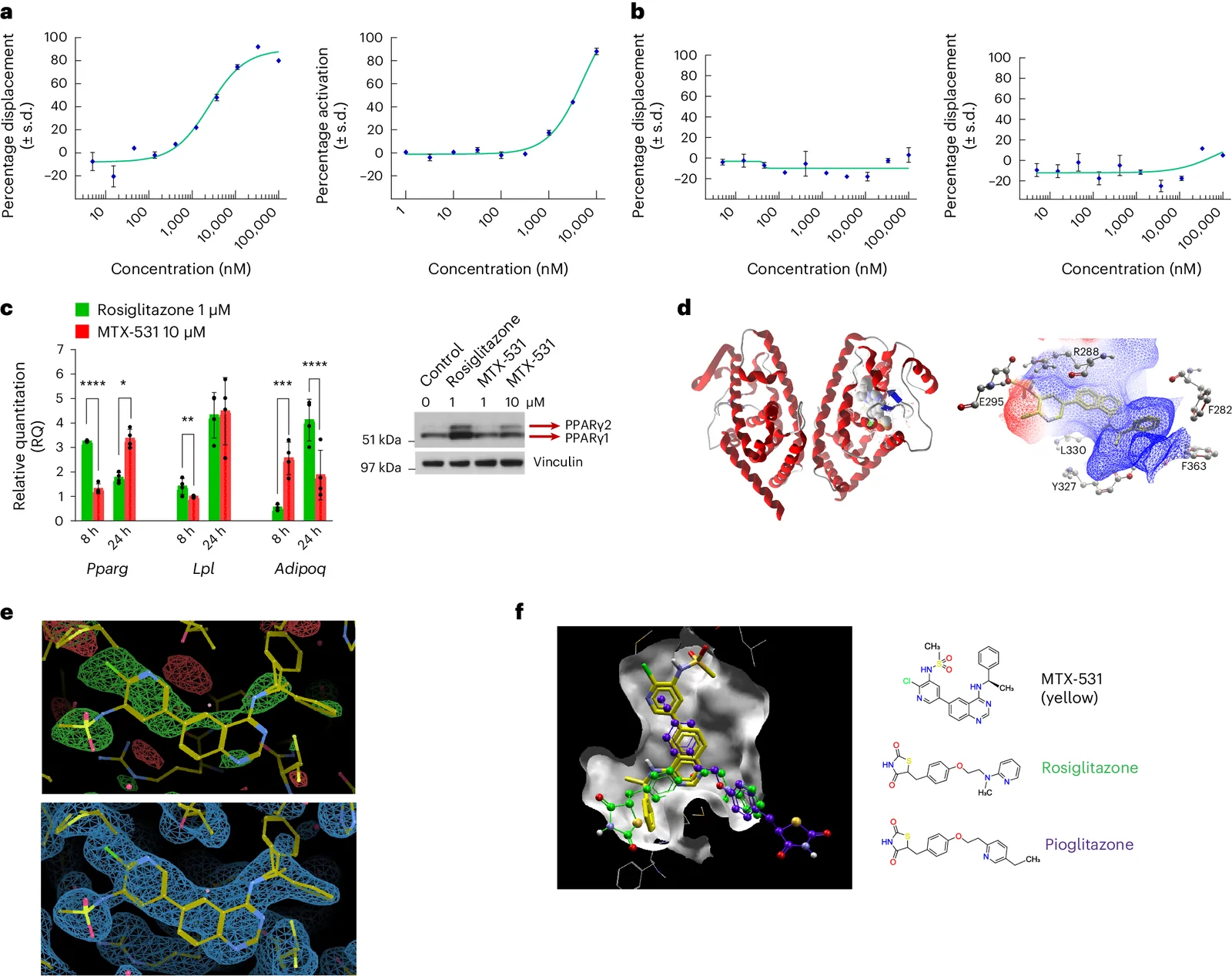
MTX-531 acts as an agonist of PPARγ. **a**, Left, in a competitive binding assay, MTX-531 displaced the PPARγ agonist GW1929 with an EC_50_ of 2.5 µM (*R*^2^ = 0.9712). Right, MTX-531 was further shown to be a weak activator of PPARγ in a cell-based transcription assay carried out in PPARγ-UAS-bla HEK 293H cells with an EC_50_ of 3.4 µM (*R*^2^ = 0.9898). Data from both studies are representative of two individual experiments carried out at concentrations tested in duplicate. **b**, MTX-531 did not exhibit activity when titrated across a ten-point concentration range to test for competitive binding to PPARα against GW7647 (left) and PPARδ against GW0742 (right). Data are representative of two studies carried out at concentrations tested in duplicate. **c**, Left, PPARγ target gene expression levels were analyzed in quadruplicate by RT–qPCR analysis of total RNA extracts from 3T3-L1 cells treated with differentiation medium containing rosiglitazone or MTX-531 for 8 or 24 h. Data are shown as the mean and upper–lower limits of RQ. A two-tailed, unpaired *t*-test or Mann–Whitney test was used to determine the statistical differences between treatment groups. *P = 0.0286, **P = 0.0213, ***P = 0.0001 and ****P < 0.0001. Right, an immunoblot analysis of PPARγ expression was carried out in 3T3-L1 adipocytes at 7 days after induction of differentiation. PCR and immunoblotting data are representative of two independent experiments. **d**, Crystal structure of PPARγ cocomplexed with MTX-531 solved at 1.9 Å (PDB 8SC9). Left, the secondary structure; right, a view of MTX-531 bound to PPARγ from the ligand-binding pocket. **e**, Electron density maps of the MTX-531-binding site of PPARγ. Top, the initial *F*_o_ − *F*_c_ difference electron density map of the model (contoured at 3.0 σ) before modeling of the compound with BUSTER. Shown is the region of the compound-binding site in chain B and the final refined coordinates. Bottom, the final 2*F*_o_ − F_c_ electron density map (contoured at 1.0 σ) resulting from refinement of the final model. Shown is the region of the compound-binding site. **f**, Overlay o**f** MTX-531 X-ray binding models with commercial PPARγ agonists rosiglitazone (PDB 4EMA) and pioglitazone (PDB 2XKW). Source data

We hypothesized that the absence of hyperglycemia in mice treated with MTX-531 stems from the molecule’s unique ability to act as an agonist of the nuclear hormone receptor PPARγ. Time-resolved fluorescence resonance energy transfer (TR-FRET) competitive binding assays showed MTX-531 to be a weak agonist of PPARγ (IC_50_ = 2.5 μM) and inactive against PPARα and PPARδ at concentrations as high as 100 μM (Fig. 7a,b). The intracellular effects of MTX-531 on PPARγ activity were evaluated in reporter gene assays carried out in 293H cells, where agonist activity was shown against PPARγ (EC_50_ = 3.4 μM; Fig. 7a, right) with significantly less potency compared to rosiglitazone (EC_50_ = 14 nM). The expression of PPARγ target genes involved in the induction of adipocyte differentiation was measured by reverse transcription (RT)–qPCR in treated 3T3-L1 cells, providing further evidence for a functional interaction between MTX-531 and PPARγ. The 3T3-L1 preadipocytes were cultured in differentiation induction medium containing MTX-531 (10 μM) or rosiglitazone (1 μM) for 8 or 24 h followed by an assessment of the gene expression of adipocyte markers PPARγ (encoded by *Pparg*), lipoprotein lipase (encoded by *Lpl*) and adiponectin (encoded by *Adipoq*) (Fig. 7c). These markers are known to be upregulated in response to rosiglitazone^37^. The maximal induction of *PPARγ* ranged from twofold to fourfold in response to both compounds, providing further support for the ability of MTX-531 to act as an agonist of PPARγ. At the protein level, the extent of upregulation in expression levels of both PPARγ1 and PPARγ2 were again consistent with the significant potency differences between rosiglitazone and MTX-531 (Fig. 7c, right).

We next sought to generate an X-ray crystal structure of MTX-531 bound to PPARγ to provide structural evidence for their interaction. The three-dimensional complex of PPARγ and MTX-531 was solved from crystals diffracted to 1.9-Å resolution (Fig. 7d). Clear electron density at the compound-binding site revealed binding of the entire compound, allowing an unambiguous placement of the ligand (Fig. 7e). The final solved structure contains two molecules of PPARγ (chains A and B) and one molecule of MTX-531 bound to chain B. The PPARγ ligand-binding region is known to be a large, mostly hydrophobic cavity capable of binding a wide variety of small molecules. The quinazoline core of MTX-531 appears to sit in a pocket of PPARγ formed by stacking of the aliphatic side chains of L330 and R288 (Fig.7d). The functional groups at the 6-position quinazoline core in MTX-531 bind at a site distinct from the orthosteric pocket that binds rosiglitazone (Fig. 7f).

## Discussion

There are few examples of approved kinase-targeted drugs that lead to durable single-agent activity. The PI3K–mTOR pathway drives resistance to a broad assortment of targeted therapies, including highly selective allosteric MEK inhibitors and covalent inhibitors of KRAS-G12C (refs. ^38–41^). Design of MTX-531 was driven by the concept of developing a single molecule capable of selectively targeting the PI3K–mTOR pathway and EGFR. Such a molecule would ostensibly be useful to deliver single-agent therapy to selected patient populations, for example, HNSCC, where EGFR and PI3K–mTOR signaling drives progression. Furthermore, this approach could provide an attractive candidate for combination with RAS pathway inhibitors, which are subverted by these resistance drivers. The combination of cetuximab and adagrasib nearly doubled the RECIST (response evaluation criteria in solid tumors) response rate compared to adagrasib monotherapy in patients with *KRAS*^G12^-mutant CRC^42^. Nonetheless, secondary resistance to cotargeting EGFR and KRAS-G12C, in some cases driven by upregulation of mTOR signaling, has been reported in CRC^35^. MTX-531, by virtue of its dual inhibitory activity against EGFR and the PI3K–mTOR pathway, offers a single-molecule strategy for overcoming multiple resistance mechanisms encountered in *KRAS*^G12C^-mutant CRC. It is promising that MTX-531 monotherapy led to tumor regressions in the two *PIK3CA*-mutant *KRAS*^G12C^-mutant CRC PDX models studied here. Like *KRAS*, activating mutations in *PIK3CA* are associated with clinical resistance of CRC to EGFR-targeted therapies^43,44^. Tumors harboring mutations in both *KRAS* and *PIK3CA* frequently require effective inhibition of dual MEK–ERK and PI3K–Akt pathway signaling^45^.

MTX-531 was rationally designed to inhibit EGFR and PI3K, selectively and concurrently. The reversed binding mode of the quinazoline core between EGFR and PI3Kγ was an important element in the design of MTX-531, which shows low-nanomolar potency against both of its intended targets. Knight and colleagues were the first to report the feasibility of designing dual inhibitors of tyrosine and phosphoinositide kinases^46^. Through iterative medicinal chemistry and X-ray crystallography, they identified molecules that adopt a single binding mode to inhibit PI3Ks and multiple tyrosine kinases with a high degree of potency. In contrast, MTX-531 is a remarkably selective ATP-competitive protein kinase inhibitor. Only four of 432 kinases tested were found to exhibit an IC_50_ < 1 μM and none came within tenfold of MTX-531’s on-target potency against EGFR (IC_50_ ≈ 15 nM). Erlotinib, which is roughly one log more potent against EGFR than MTX-531, was reported to exhibit an IC_50_ < 1 μM against a minimum of 20 off-target kinases^47^.

Preclinical proof of concept supporting advanced development of MTX-531 comes in part from monotherapy studies conducted against HNSCC models, where objective responses were observed in all models tested. While every HNSCC PDX model studied here was mutated in *PIK3CA*, it remains unclear whether this marker is a prerequisite for efficacy. We find this unlikely because genes encoding multiple kinases in the PI3K–mTOR pathway are mutated in HNSCC, including other *PIK3C* isoforms, *AKT*, *PTEN*, *MTOR* and *RICTOR*^7^. Furthermore, differences in *PIK3CA* hotspots are observed when comparing human papillomavirus (HPV)^+^ to HPV^−^ cases. *PIK3CA* mutations occur predominantly in the helical domain in HPV^+^ HNSCC but throughout the gene in HPV^−^ HNSCC^48^. It is noteworthy that the exceptional responder model NCI 944545-341-R is HPV^−^ and the only PDX model tested here that harbors a mutation (H1047R) in the *PIK3CA* kinase domain. Furthermore, the expression of both pPRAS40 and p4E-BP1 was significantly upregulated in 944545-341-R (Extended Data Fig. 4b). Interestingly, pPRAS40 has been reported to correlate with insulin-like growth factor 1 receptor-induced resistance to EGFR inhibition in HNSCC^49^. PRAS40 is an inhibitor of mTOR that leads to increased mTOR signaling upon phosphorylation by Akt^50^. Given the pan-PI3K–mTOR-inhibitory profile of MTX-531, its striking activity against 944545-341-R tumors is perhaps not surprising. mTOR has been reported to restrain the tumor suppressor function of 4E-BP1 by phosphorylation, which can be reactivated in response to mTOR inhibition^51^.

The effectiveness of MTX-531 at inhibiting both EGFR and PI3K–mTOR signaling is likely a major factor in its strong efficacy against *KRAS*^G12C^-mutant CRC. EGFR signaling has been identified as the dominant mechanism of CRC resistance to KRAS-G12C-targeted agents^33^. The later discovery that cotargeting of EGFR and KRAS-G12C leads to upregulation of mTOR signaling illustrates the robust adaptive capacity of these tumors to rely on bypass signaling routes to survive^35^. Targeting mTOR signaling to prevent adaptive resistance to KRAS inhibitors has also been reported for non-small cell lung cancer and pancreatic cancer models^52,53^. The importance of sustained mTOR inhibition to prevent resistance also extends to therapeutics targeting EGFR or PI3K^54^.

Because the PI3K–Akt–mTOR pathway is the most frequently dysregulated pathway in human cancers, it has received intense scrutiny from the drug discovery community. Consequently, a multitude of drug candidates targeting this pathway have advanced to the clinic^9^. Unfortunately, most trials have failed because of poor tolerability, drug resistance and inability to deliver single-agent efficacy. MTX-531 has the potential to challenge the widely accepted view that dual PI3K–mTOR inhibitors are inherently more toxic than isoform-specific PI3K inhibitors. Treatment with MTX-531 led to complete regressions of CAL-33 xenografts over a wide dose range spanning 25 to 100 mg kg^−1^. In contrast, alpelisib (BYL-719, Piqray), the only clinically approved PI3Kα-selective inhibitor, resulted in stasis but not regressions of CAL-33 tumors^55^. The favorable therapeutic index seen with MTX-531 in preclinical animal studies allays concerns regarding overlapping toxicities incurred from cotargeting EGFR and PI3K. MTX-531 represents a unique single-molecule approach to balanced combination therapy that minimizes off-target effects while eliminating the need to optimize the pharmacokinetic profiles of multiple agents. Whereas genomic heterogeneity may impact the relative contribution of EGFR versus PI3K inhibition to the overall activity of MTX-531 against individual tumors, the reciprocal roles of these kinases in driving adaptive resistance will likely promote the durability of response.

It is noteworthy that hyperglycemia was reported to be problematic when the approved PI3K inhibitors, alpelisib or copanlisib, were combined with cetuximab in clinical trials^12^. The finding that MTX-531 does not lead to PI3K inhibitor-induced hyperglycemia in mice is encouraging because that side effect is common to both mice and humans. In clinical trials, hyperglycemia has become a surrogate biomarker for the demonstration of effective PI3K inhibition resulting from a disruption of systemic glucose homeostasis. Interestingly, PI3Kα-selective inhibitors have been reported to possess an improved preclinical therapeutic window compared to pan-PI3K inhibitors based on their reduced propensity for hyperglycemia^56^. However, we found that PI3K inhibitors from both classes led to an elevation in blood glucose levels, a property not shared with MTX-531. Our working hypothesis is that the agonistic activity of MTX-531 against PPARγ counteracts PI3K-driven hyperglycemia. PPARγ agonists are commonly used in the treatment of type 2 diabetes to increase sensitivity to insulin. Consistent with our data, Powis and colleagues reported that the thiazolidinedione pioglitazone prevents hyperglycemia caused by the PI3K inhibitor PX-866 (ref. ^57^). MTX-531 is a pan-PI3K inhibitor that uniquely does not cause hyperglycemia. Consistent with this observation, mice treated with MTX-531 do not need to be placed on a ketogenic diet to be active against highly aggressive KPC tumors and may be less susceptible to the insulin-mediated reactivation of PI3K signaling reported for alpelisib^36^.

Ultimately, MTX-531 will require clinical testing to validate its favorable preclinical toxicity profile. If this molecule proves to be well tolerated in patients, its versatility in both single-agent and combination settings offers a breadth of development strategies that continue to evolve as combination candidates become available. Future avenues of exploration include *KRAS*^G12D^-mutant cancers where the HER family and the S6 pathway impact the response to KRAS-G12D inhibitors^58^. As a single agent, MTX-531 could be envisioned to have a role in the treatment of not only HSNCC but also squamous lung cancers^59^, as well as a subset of triple-negative breast cancers driven by EGFR and PI3K^60^. MTX-531, which has the unique capability of concurrently and selectively inhibiting EGFR, PI3K and mTOR, illustrates the power of rational computational drug design to target multiple adaptive resistance mechanisms in a single molecule.

## Methods

### Crystallization studies

The specific constructs used were G696-G1022 (Protein Data Bank (PDB) 2GS2) for EGFR^61^, S144-A1102 (PDB 1E8Y) for PIK3CG^62^ and L232-Y505 of UniProt entry P37231-2 (PDB 3SZ1) for PPARG^63^.

Crystals of apo-EGFR were obtained using hanging-drop vapor diffusion setups and EGFR at a concentration of 5.6 mg ml^−1^ (25 mM Tris-HCl, 50 mM NaCl and 2 mM DTT; pH 7.5). The protein solution (2 μl) was mixed with 0.6 μl of reservoir solution (0.10 M HEPES–NaOH pH 6.70 and 1.05 M Na-succinate pH 7.00) and equilibrated at 12 °C over 0.4 ml of reservoir solution. Well-diffracting crystals were selected for data collection after 13 days. Crystals were soaked for 3.5 h with 1 mM MTX-531 in 30% solubilizing mix 1 (12.5% v/v diethylene glycol, 25% v/v ethylene glycol, 12.5% v/v 1,2-propanediol, 25% v/v dimethyl sulfoxide and 25% v/v 1,4-dioxane).

Crystals of apo-PI3Kγ were obtained using hanging-drop vapor diffusion setups and PI3Kγ at a concentration of 7.1 mg ml^−1^ (20 mM Tris-HCl, 0.5 mM ammonium sulfate, 5 mM DTT, 0.02% (v/v) CHAPS and 1% ethylene glycol; pH 7.2). Then, 1 μl of the protein solution was mixed with 1 μl of reservoir solution (100 mM Tris-HCl pH 8.2, 0.2 M lithium sulfate and 15% (w/v) PEG 4000) and equilibrated at 20 °C over 300 μl of reservoir solution. Well-diffracting crystals were selected for soaking after 20 days. Crystals were soaked with 5 mM MTX-531 for 16 h in 30% ligand solubilization mix 4 (12.5% v/v diethylene glycol, 12.5% v/v glycerol, 12.5%, v/v 1,2-propanediol, 25% v/v dimethyl sulfoxide and 25% v/v 1,4-dioxane).

Crystals of apo-PPARγ were obtained using sitting-drop vapor diffusion setups. An aliquot (0.2 μl) of the PPARγ protein solution at a concentration of 8.9 mg ml^−1^ (20 mM Tris-HCl, 100 mM NaCl, 5% glycerol and 1 mM TCEP; pH 8.0) was mixed with 0.1 μl of reservoir solution (0.10 M Bis-Tris-propane pH 9.00 and 2.20 M (NH_4_)_2_-sulfate) and equilibrated at 20 °C over 70 μl of reservoir solution. Well-diffracting crystals were selected for data collection after 11 days. Crystals were soaked for 20 h with 2.5 mM MTX-531.

Complete datasets of 2.0 Å, 2.7 Å and 1.9 Å were collected for EGFR–MTX-531 and PI3Kγ–MTX-531 crystals at the European Synchrotron Radiation Facility (beamline ID30a1). A complete 1.9-Å dataset of a PPARγ–MTX-531 crystal was collected at the Swiss Light Source (Paul Scherrer Institute, beamline PXI). Refinement statistics for these X-ray crystal structures are provided in Supplementary Table 5. The data were integrated, analyzed and scaled by the programs XDS, Pointless, Aimless and STARANISO from within the autoPROC pipeline.

### Biochemical kinase assays

All biochemical kinase profiling was carried out at Thermo Fisher Scientific using the Z-Lyte, LanthaScreen or Adapta assay formats as previously reported by our group^64^. All assays were carried out at the predetermined ATP *K*_m_ apparent for that kinase. Dose–response curves were curve-fit to model number 205 (sigmoidal dose–response model) using XLfit graphing software from IDBS.

### Nuclear receptor assays

LanthaScreen TR-FRET competitive binding assays were carried out at Thermo Fisher Scientific for screening MTX-531 against the family of PPAR nuclear hormone receptors. Compounds were screened in 1% DMSO (final concentration) and threefold serial dilutions were conducted for ten-point titrations. A known inhibitor was titrated on each plate (GW1929 (PPARγ), GW7647 (PPARα) and GW0742 (PPARβ)) to ensure that reference compounds were displaced within an expected IC_50_ range previously determined. Dose–response curves were curve-fit to model number 205 (sigmoidal dose–response model) using XLfit graphing software from IDBS.

Intracellular effects on the PPARγ nuclear hormone receptor were determined at Thermo Fisher Scientific using GeneBLAzer β-lactamase reporter technology. PPARγ-UAS-bla HEK 293T cells were thawed and resuspended in assay medium (DMEM phenol red free, 2% CD-treated FBS, 0.1 mM NEAA, 1 mM sodium pyruvate and 100 U per ml + 100 μg ml^−1^ penicillin–streptomycin) to a concentration of 9.4 × 10^5^ cells per ml. Then, 4 μl of a tenfold serial dilution of rosiglitazone (control agonist starting concentration, 316 nM) or MTX-531 was added to appropriate wells of a 384-well poly(d-lysine) assay plate. Next, 32 μl of cell suspension (30,000 cells) was added to each well, followed by 4 μl of assay medium to bring the final assay volume to 40 μl. Plates were incubated for 16–24 h at 37 °C, 5% CO_2_ in a humidified incubator. Plates were incubated at room temperature for 2 h followed by determination of fluorescence. A *z*′ factor of ≥0.5 was required to meet the quality control criteria.

### Reagents, cell lines and PDX models

MTX-531 was synthesized at WuXi AppTec. Alpelisib (A-4477), erlotinib (E-4997) and trametinib (T-8123) were purchased from LC Labs. Sotorasib (C-1499) was purchased from Chemgood. Omipalisib (6792) was purchased from Tocris. Buparlisib (HY-70063), copanlisib (HY-15346), taselisib (HY-13898) and OTSSP167 (HY-15512A) were purchased from MedChemExpress. Rosiglitazone (NC9560589) was purchased from Cayman Chemical. Bovine insulin (I0516), hydrocortisone (H4001) and 1-methyl-3-isobutylxanthine (IBMX; I5879) were purchased from MilliporeSigma. Dexamethasone (API-04) was purchased from G Biosciences. Human epidermal growth factor (hEGF; PHG0313) was purchased from Gibco (Thermo Fisher Scientific).

CAL-27 (ACC 446) and CAL-33 (ACC 447) cell lines were obtained from the Leibniz Institute DSMZ German Collection of Microorganisms and Cell Cultures. BICR 16 (06031001) and BICR 56 (06031002) cell lines were obtained from the European Collection of Authenticated Cell Cultures. MIA PaCa-2 (CRL-1420), 3T3-L1 (CL-173) and Detroit 562 (CCL-138) cell lines were obtained from the American Type Culture Collection (ATCC). The MOC1 (EWL001-FP) cell line was obtained from KeraFast. KPC tumors originated from 65-671 cells (FVB/N strain), which were kindly provided by M. Pasca di Magliano (University of Michigan)^65^. The following models were received from the NCI PDMR as cryopreserved cells: 135848-042-T, 455876-151-R, 848979-319-R and CN0375-F725-PDC. The following models were received from the NCI PDMR as cryopreserved tumor fragments: 245127-232-R, 354836-022-R and 944545-341-R. The PDX model CRC 14-929 was established at the University of Michigan (Leopold lab)^66^. The PDX models B8239 and B8324 were developed at the MD Anderson Cancer Center (Kopetz lab). Animal models established from persons with CRC required informed consent under institutional review board-approved protocols HUM00065489 and LAB10-0982 at the University of Michigan and MD Anderson Cancer Center, respectively. Demographic data for the PDX models used in this study are provided in Supplementary Table 6.

### Cell culture

RPMI 1640 medium (11875-093), DMEM (11965-092), penicillin–streptomycin (15150-122), GlutaMax (35050-061), sodium pyruvate (11360-070) and PBS (10010-023) were all obtained from Gibco (Thermo Fisher Scientific). FBS was obtained from Cytivia (SH3039603). Calf bovine serum (CBS) was purchased from the ATCC (30-2022). Cells were grown in the appropriate medium and maintained at 37 °C in a humidified incubator with 5–10% CO_2_. Standard operating procedures issued by the NCI PDMR were followed to thaw, expand and maintain the PDC lines. All cell lines were determined to be *Mycoplasma* free using Lonza’s MycoAlert *Mycoplasma* Detection kit (Lonza). Cell lines were authenticated by short tandem repeat (STR) analysis (ATCC, human and mouse STR profiling service).

### Adipocyte differentiation

The 3T3-L1 cells were differentiated according to methods published previously^67^. Cells were seeded at a density of 6.0 × 10^5^ cells per T-75 flask (Corning) in DMEM supplemented with 10% CBS, 100 U per ml penicillin, 100 mg ml^−1^ streptomycin and 1 mM sodium pyruvate and grown to confluency. Cells were grown for an additional 48 h after the addition of fresh medium. To induce differentiation, the cells were cultured for 48 h in differentiation induction medium containing DMEM supplemented with 10% FBS, 100 U per ml penicillin, 100 mg ml^−1^ streptomycin, 1 mM sodium pyruvate, 1.0 µM dexamethasone, 0.5 mM IBMX and 1.0 μg ml^−1^ insulin containing DMSO, 1 μM rosiglitazone or 10 μM MTX-531. Thereafter, the medium was changed to DMEM, 10% FBS, 100 U per ml penicillin, 100 mg ml^−1^ streptomycin, 1 mM sodium pyruvate and 1.0 μg ml^−1^ insulin every 48 h until 7 days after induction.

### Immunoblot analysis

To generate cell lysates for immunoblot analysis, cells were seeded 6.0 × 10^5^ cells per dish in 60-mm tissue culture plates with the appropriate growth medium, including all supplements. The following day, the medium was removed from the dishes and replaced with the appropriate serum-free growth medium. The next day, the cells were treated with DMSO or MTX-531 at the indicated concentrations. At 15 min before lysis, the cells were stimulated with hEGF. Cells were lysed at 2 h following treatment.

Cells were washed with cold PBS and lysed with NP-40 lysis buffer (50 mM Tris pH 7.5, 1% NP-40, 150 mM NaCl, 10% glycerol and 1 mM EDTA) supplemented with protease and phosphatase inhibitors (Roche). Tumors were minced and manually homogenized in NP-40 lysis buffer. Lysates were rocked at 4 °C for 30–60 min and cleared by centrifugation. Equal amounts of protein (10–20 μg) in lysates normalized using BioTek Gen5 software were resolved by SDS–PAGE, transferred to 0.2-µm or 0.45-µm polyvinyldifluoride (PVDF) membranes (MilliporeSigma) and probed with specific primary and secondary antibodies. Bands representing the proteins of interest were detected by chemiluminescence with enhanced chemiluminescence detection reagents (Cytiva). Western blot images were acquired using the ChemiDoc Imaging System (12003153) purchased from Bio-Rad.

Unless otherwise noted, the following primary antibodies were used at 1:1,000 dilution: anti-pEGFR (Y1068) 3777, anti-pEGFR (Y1068) 2234, anti-EGFR 2646 (1:10,000), anti-pAkt (T308) 13038, anti-pAkt (S473) 4060, anti-Akt 9272 (1:5,000), anti-pS6 (S235 or S236) 4857, anti-S6 2217 (1:10,000), anti-pPRAS40 (T246) 2997, anti-PRAS40 2691 (1:10,000), anti-p4E-BP1 (S65) 9451, anti-4E-BP1 9644, anti-pp70S6K (T389) 97596, anti-p70S6K 9202, anti-PPARγ 2443, anti-cleaved PARP 9541 (all from Cell Signaling Technologies), anti-β-actin HRP-conjugated 197277 (1:10,000) and anti-vinculin 129002 (1:10,000) (both from Abcam). AffiniPure goat anti-rabbit IgG secondary antibody (Jackson ImmunoResearch Laboratories, 111-035-003) was used at 1:10,000 dilution.

The pixel intensity for each selected protein band was quantified using ImageJ software. The images were converted to grayscale and saved as .TIF files. A rectangular region of interest (ROI) was drawn around the largest band for each protein on a given blot. The same ROI was applied to each band for a given protein and histograms were generated indicating the pixel intensity. The ratio of the signal intensity of the phosphorylated protein to the housekeeping protein was calculated for each target protein of interest using Excel. The percentage inhibition was calculated as the ratio of the signal intensity of the treatment group to the control-treated samples.

### RT–qPCR Analysis of PPARγ Target Genes

Total RNA was extracted from 3T3-L1 cells using Trizol (Invitrogen) according to the manufacturer’s instructions at 8 and 24 h following the addition of differentiation induction medium containing DMSO, rosiglitazone (1 µM) or MTX-531 (10 µM). RNA extracts were treated with the RNAse-Free DNase Kit and purified using the RNeasy MinElute Cleanup Kit (Qiagen). RNA extracts were quantified and assessed for quality with the RNA BR Assay Kit and the RNA IQ kit, respectively, using a Qubit 4 Flourometer (Invitrogen). The SuperScript VILO complementary DNA (cDNA) Synthesis Kit (Invitrogen) was used to synthesize first-strand cDNA by combining 20 µl of 5× VILO reaction mix, 10 µl of 10× SuperScript enzyme mix and 65 µl of RNAse-free distilled H_2_O with 5 µg of RNA in a 100-µl reaction volume. A Veriti 96-well thermal cycler (Applied Biosystems) was used to amplify the PCR reactions using the following conditions: 10 min at 25 °C, 90 min at 42 °C and 5 min at 85 °C. Reactions without reverse transcriptase were included to detect genomic DNA contamination. The qPCR reactions were performed in quadruplicate using mouse-specific Taqman Gene Expression Single-Tube Assays (Applied Biosystems) for lipoprotein lipase (*Lpl*) (Mm00434764_m1), *Pparg* (Mm00440940_m1) and *Adipoq* (Mm04933656_m1). The housekeeping gene, *Rplp0* (Mm00725448_s1), was used to normalize target gene expression. Reactions were prepared according to the manufacturer’s instructions. Control reactions without cDNA template were included to detect contamination. The amplification and analysis of PCR reactions were performed using the QuantStudio 5 Real-Time PCR System (Applied Biosystems) programmed as follows: 20 s at 95 °C followed by 40 cycles at 95 °C for 1 s and annealing and extending for 20 s at 60 °C. The fold gene expression (RQ) was calculated using the formula, RQ = 2^(−ΔΔ*C*t)^ where ΔΔ*C*_t_ = Δ*C*_t_ (treated sample) − Δ*C*_t_ (DMSO-treated sample) and Δ*C*_t_ = (target gene *C*_t_ − *Rplp0* reference gene *C*_t_). Data are represented as the mean RQ, RQ_max_ = 2^−(RQ + s.e.m.)^ and RQ_min_ = 2^−(RQ − s.e.m.)^. The analysis of statistical differences between treatment groups was performed using an unpaired *t*-test or Mann–Whitney test.

### In vivo xenograft studies

All procedures related to animal handling, care and treatment were performed under an approved protocol (PRO00010150) according to the guidelines set forth by the University of Michigan Institutional Animal Care and Use Committee (IACUC) and following the guidance of the Association for Assessment and Accreditation of Laboratory Animal Care (AAALAC). Animals were housed per institutional guidelines as determined by the affiliated IACUC, consisting of typical 12-h light–dark cycles in ambient temperatures of 68–75 °C with 30–70% humidity. Mice were maintained under pathogen-free conditions and food and water were provided ad libitum. Sex was not considered in the study design because these studies center around cancer signaling and drug targets that generally do not require sex-based considerations. Only female mice were used because of animal housing considerations.

For human-derived cell lines and PDX models, 6–8-week-old female CIEA NOG mice (NOD.Cg-*Prkdc*^*scid*^ *Il2rg*^*tm1Sug*^/JicTac from Taconic) or female athymic homozygous nude mice (CrTac:NCr-*Foxn1*^*nu*^ from Taconic or Crl:NU(NCr)-*Foxn1*^*nu*^ from Charles River Laboratories) were used. For models originating from cell culture (CAL-27, CAL-33, MIA PaCa-2, 135848, 455876, 848979 and CN0375), mice were inoculated subcutaneously in the right axilla with 1 × 10^6^–5 × 10^6^ cells suspended in 100 µl of a 1:1 ratio of serum-free medium to Matrigel. For models originating from tumor fragments (UM-CRC 14-929, 245127, 354836, 944545, B8318 and B8324), mice were implanted with tumor fragments 2–3 mm in diameter into the right axilla by trocar.

For the KPC model, female inbred FVB mice (6–8 weeks of age) were obtained from Taconic (FVB/NTac). Mice were inoculated subcutaneously in the right axilla with 1 × 10^6^ 65-671 (FVB/N strain) cells suspended in 100 µl of a 1:1 ratio of serum-free medium to Matrigel.

MTX-531 was prepared as a suspension in a 1:2 ratio of propylene glycol to 1% Tween 80–Na_3_PO_4_ or as a solution in 50% propylene glycol, 35% PEG400 and 10% TPGS in water with 5% 1 N NaOH and administered by oral gavage. Compounds were prepared fresh daily and administered according to individual mouse body weight (0.2 ml per 20 g).

On reaching a mean tumor volume of ~100 to 200 mm^3^, mice were randomized into treatment arms before initiation of treatment on day 1 of study. For the advanced-stage CAL-33 xenograft study, mice were randomized into treatment arms when the mean tumor volume was ~500 mm^3^ before treatment initiation. Subcutaneous tumor volume and body weights were measured 2–3 times a week. Tumor volumes were calculated by measuring two perpendicular diameters with calipers and using the formula, tumor volume = (length × width^2^)/2. Mice were treated and monitored daily until individual mouse tumor burdens reached IACUC-approved limitations (either 500 mm^3^ for ulcer-prone models or 1,000 mm^3^). The percentage change in treated versus control mice was calculated on the day the median control mouse was euthanized as follows: [(T_final_ − T_initial_)/(C_final_ − C_initial_)] × 100, where C_initial_ and C_final_ are the median tumor volumes on the first day of treatment and the day indicated for the vehicle control group and T_initial_ and T_final_ are the median tumor volumes on the first day of treatment and the day indicated for the treated group. The increase in lifespan was calculated as [(T_day_ − C_day_)/C_day_] × 100, where T_day_ is the day the median treated mouse was killed and C_day_ is the day the median control mouse was euthanized. No statistical methods were used to predetermine group sizes but our group sizes were similar to those reported in previous publications^33,36,58,66,68^. For pharmacodynamic studies, when tumors reached a mean tumor volume of ~150–300 mm^3^, mice were randomized into treatment arms and treated with the vehicle or test article. At the indicated time points, mice were euthanized and tumors were isolated, snap-frozen in liquid nitrogen and stored at −80 °C.

For animal studies, quantitative data are presented as the mean ± s.e.m. Animals were randomized on the basis of tumor size before treatment. All animals treated were included in the analyses. Data collection and analysis were not performed blind to the conditions of the experiments.

The maximal tumor size allowed by the protocol approved by the IACUC at the University of Michigan was 2,000 mm^3^. This maximal tumor size was not exceeded for any animals used in these experiments.

#### Modified RECIST in preclinical efficacy studies

For the control animals, the percentage change in tumor volume was calculated when the tumor volume reached a predetermined size of 500 mm^3^ or 1,000 mm^3^, depending on the tumor model. This percentage change in tumor volume was calculated from baseline as follows: ΔT = (T_final_ − T_initital_)/T_initial_ × 100. For treated animals, the response was determined by comparing the tumor volume change at time *t* to its baseline: ΔT = (T_final_ − T_initital_)/T_initial_ × 100. The best overall response for each animal was defined as the minimum percentage change in tumor volumeoccurring after the first 7 days of treatment.

The preclinical criteria for response were adapted from RECIST 1.1 (refs. ^68,69^) and defined as follows: a complete response (CR) is the disappearance of the subcutaneous tumor (tumor no longer palpable); a partial response (PR) is a ≥30% decrease in tumor volume; progressive disease (PD) is defined as a >2-fold increase in tumor volume; stable disease (SD) is defined as neither sufficient tumor shrinkage to qualify as a PR nor sufficient growth to qualify as PD.

### Glucose and insulin measurements

Female athymic nude mice (8–10 weeks of age) were acclimated for a minimum of 3 days before study initiation. Mice were administered the vehicle control or test article on the basis of individual body weight (0.2 ml per 20 g). For the assessment of blood glucose levels, 1–2 drops of blood were taken from the tails of the mice and measured using an Accu-Chek Aviva glucometer at the indicated time point before or after treatment. For the assessment of blood insulin levels, ~30–40 µl of blood was collected into EDTA microvette tubes immediately following blood glucose measurement, centrifuged at 4 °C for 20 min at 2,000*g* to collect plasma and stored at −80 °C. The insulin levels in plasma and tumor samples were determined by ELISA (Crystal Chem, cat. no. 90080).

### Statistical analyses

Quantitative data are presented as the mean ± s.e.m. For animal studies, animals were randomized before treatment and all animals treated were included for the analyses. For assessing the treatment effects over time, we used a linear mixed effects model with treatment as the fixed effect and time as the random effect. A Wald test was used for the significance comparisons among different treatment groups. Individual data points were included for all data presented. The exact models are detailed in the Supplementary Information. The analyses were run with R package LME4 and were versified using SAS version 9.4.

### Pharmacokinetic studies

Plasma samples were collected in CD1 mice from the dorsal metatarsal vein at all time points except 24 h. For the 24-h time point, samples were collected after cardiac puncture. A volume of 0.05–0.1 ml was collected at each time point. K_2_-EDTA was used as the anticoagulant. Samples were centrifuged at 4,000*g* for 5 min at 4 °C to obtain plasma and stored at −75 ± 15 °C before analysis. WinNonlin (Phoenix version 6.1) or similar software was used for pharmacokinetic calculations.

### Synthesis of MTX-531

The synthesis scheme for MTX-531 is included in the Supplementary Information.

### Reporting summary

Further information on research design is available in the Nature Portfolio Reporting Summary linked to this article.

## Supplementary information

Supplementary Information Supplementary note describing the synthetic scheme for MTX-531. Supplementary discussion of the statistical analysis of PDX efficacy studies.

Reporting Summary

Supplementary Table 1 Workbook containing Supplementary Tables 1 (structure–activity relationships leading to the prelead molecule MTX-211), 2 (evolution of MTX-531), 3 (broad kinome screening of MTX-531), 4 (testing of MTX-531 in Eurofins SafetyScreen 87 panel of enzyme and uptake assays), 5 (refinement statistics for EGFR, PI3Kγ and PPARγ X-ray crystal structures) and 6 (demographic data for PDX models).

Supplementary Data 1 Statistical source data.

## Source data

Source Data Fig. 1 Statistical source data.

Source Data Fig. 2 Unprocessed western blots.

Source Data Fig. 2 Statistical source data.

Source Data Fig. 3 Statistical source data.

Source Data Fig. 3 Unprocessed western blots.

Source Data Fig. 4 Statistical source data.

Source Data Fig. 4 Unprocessed western blots.

Source Data Fig. 5 Statistical source data.

Source Data Fig. 5 Unprocessed western blots.

Source Data Fig. 6 Statistical source data.

Source Data Fig. 7 Statistical source data.

Source Data Fig. 7 Unprocessed western blots.

Source Data Extended Data Fig. 1 Statistical source data.

Source Data Extended Data Fig. 1 Unprocessed western blots.

Source Data Extended Data Fig. 2 Statistical source data.

Source Data Extended Data Fig. 2 Unprocessed western blots.

Source Data Extended Data Fig. 3 Statistical source data.

Source Data Extended Data Fig. 4 Statistical source data.

Source Data Extended Data Fig. 4 Unprocessed western blots.

Source Data Extended Data Fig. 4 Statistical source data.

Source Data Extended Data Fig. 5 Statistical source data.

Source Data Extended Data Fig. 6 Statistical source data.

Source Data Extended Data Fig.6 Unprocessed western blots.

## Data Availability

Cocrystal structures that support the findings of this study were deposited to the PDB under accession numbers 8SC7, 8SC8 and 8SC9 and are listed in in the pertinent figure legends and Supplementary Table 5. Data supporting the findings of this study are available from the corresponding author upon reasonable request. Source data are provided with this paper.
