# Author Correction: A first-in-class selective inhibitor of EGFR and PI3K offers a single-molecule approach to targeting adaptive resistance

**DOI:** 10.1038/s43018-026-01269-1

**Published:** 2026-10-12

**Authors:** Christopher E. Whitehead, Elizabeth K. Ziemke, Christy L. Frankowski-McGregor, Rachel A. Mumby, June Chung, Jinju Li, Nathaniel Osher, Oluwadara Coker, Veerabhadran Baladandayuthapani, Scott Kopetz, Judith S. Sebolt-Leopold

**Affiliations:** 1 Department of Radiology, University of Michigan, Ann Arbor, MI USA; 2 MEKanistic Therapeutics, Inc., Ann Arbor, MI USA; 3 Department of Biostatistics, The University of Michigan School of Public Health, Ann Arbor, MI USA; 4 The University of Texas MD Anderson Cancer Center, Houston, TX USA; 5 University of Michigan Rogel Cancer Center, Ann Arbor, MI USA; 6 Department of Pharmacology, University of Michigan, Ann Arbor, MI USA

**Keywords:** Cancer, Drug discovery

Correction to: *Nature Cancer* https://doi.org/10.1038/s43018-024-00781-6, published online 11 July 2024.

In the version of the article originally published, due to a clerical error when transferring the insulin ELISA values from mouse serum from the original data file generated by the plate reader, three measurements were inadvertently duplicated in Fig. 6b and the corresponding source data file. The underlying experiment was performed using samples from six mice, and Fig. 6b and the accompanying source data file have been corrected to reflect the complete set of original measurements, as seen in Fig. 1 below.

**Fig. 1:**
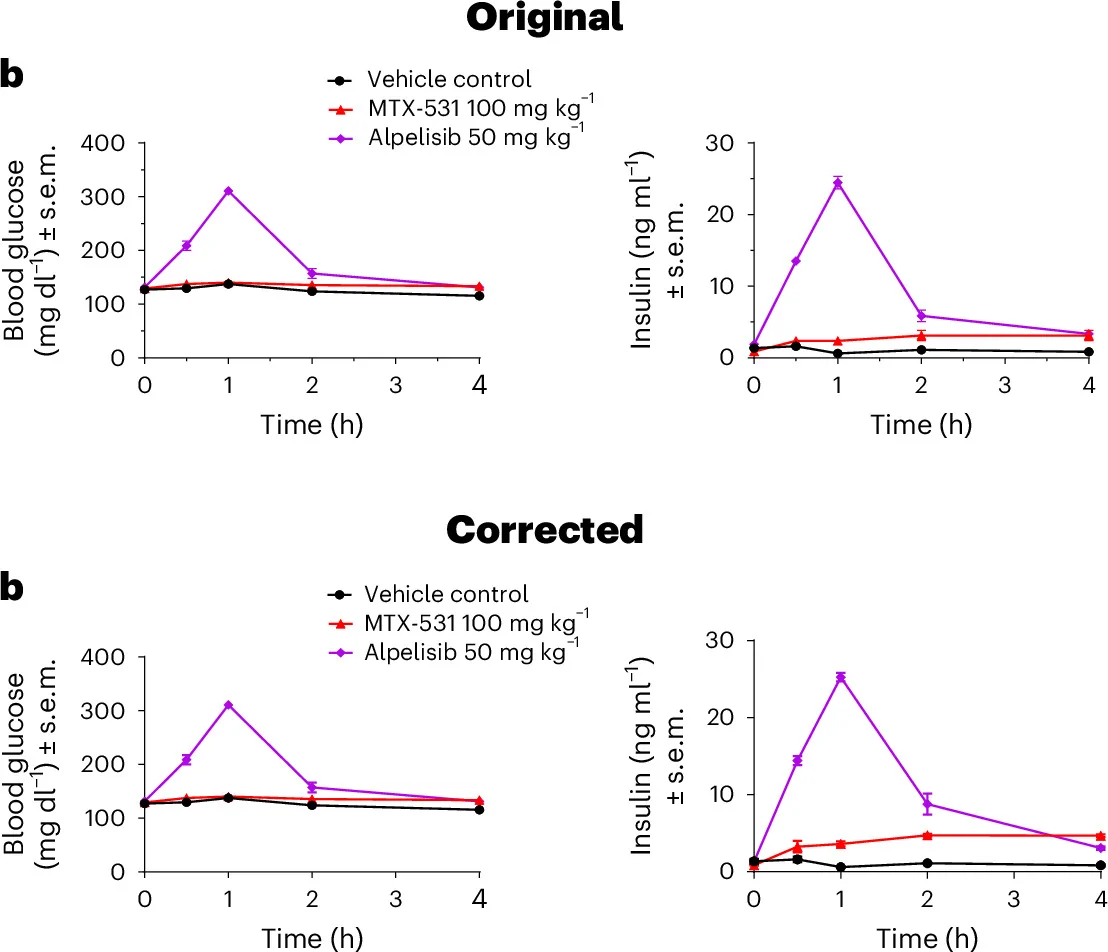
**Original and corrected Fig. 6b.**

