# Supplementary material for: A first-in-class selective inhibitor of EGFR and PI3K offers a single-molecule approach to targeting adaptive resistance
Source: Nat Cancer. 2024 Jul 11;5(8):1250–66. doi: 10.1038/s43018-024-00781-6 (PMC11357990; doi:10.1038/s43018-024-00781-6)
Supplement: Supplementary file 1 — Supplementary note describing the synthetic scheme for MTX-531. Supplementary discussion of the statistical analysis of PDX efficacy studies. [file 43018_2024_781_MOESM1_ESM.pdf]

nature cancer

Supplementary information https://doi.org/10.1038/s43018-024-00781-6

A first-in-class selective inhibitor of EGFR and PI3K offers a single-molecule approach to targeting adaptive resistance

In the format provided by the authors and unedited

Supplementary Note

Scheme 1. Synthesis of MTX-531

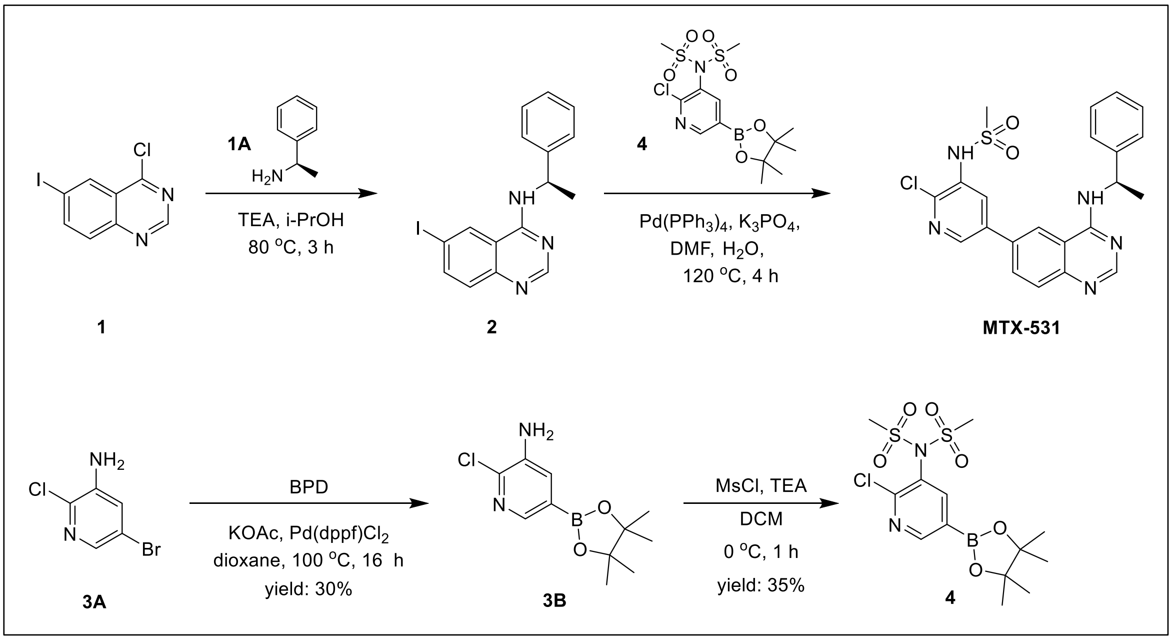

Synthesis of 6-iodo-N-[(1R)-1-phenylethyl]quinazolin-4-amine (2)

(1R)-1-phenylethanamine (208.58 mg, 1.72 mmol, 219.10 uL, 1 *eq*) and TEA (278.67 mg, 2.75 mmol, 383.32 uL, 1.6 *eq*) was added to a stirred solution of 4-chloro-6-iodo-quinazoline (500 mg, 1.72 mmol, 1 *eq*) in i-PrOH (5 mL) and the mixture was stirred at 80 °C for 3 h. LCMS showed that the starting material was consumed completely and desired MS was detected. The reaction mixture was concentrated in vacuum to give a crude product. Compound 6-iodo-N-[(1R)-1-phenylethyl]quinazolin-4-amine (800 mg, crude) was obtained as an off-white solid.

MS $(M + H)^+$ =375.9

Synthesis of 2-chloro-5-(4, 4, 5, 5-tetramethyl-1, 3, 2-dioxaborolan-2-yl) pyridin-3-amine (3B)

Pd(dppf)Cl_2.CH_2Cl_2 (5.51 g, 6.75 mmol, 0.1 *eq*), AcOK (19.87 g, 202.45 mmol, 3 *eq*), and BPD (17.14 g, 67.48 mmol, 1 *eq*) was added to a stirred solution of 5-bromo-2-chloro-pyridin-3-amine (14 g, 67.48 mmol, 1 *eq*) in dioxane (200 mL). The mixture was purged with N_2 for 3 times and stirred at 100 °C for 16 h. LCMS showed starting material was consumed completely and desired MS was detected. The reaction mixture was cooled to room temperature, quenched by water (80mL) and extracted with ethyl acetate (100 mL – 2 times). The combined organics were washed with brine (80 mL), dried over Na_2SO_4, filtered and concentrated under reduced pressure to give a residue. The residue was purified by prep-HPLC (ISCO 80 g silica, 10-60 % ethyl acetate in petroleum ether, gradient over 30 min). Compound 2-chloro-5-(4, 4, 5, 5-tetramethyl-1, 3, 2-dioxaborolan-2-yl) pyridin-3-amine (4 g, 15.72 mmol, 23.29% yield) was obtained as a yellow solid.

^{1}H NMR (400 MHz, DMSO-d6) δ ppm 7.77 (s, 1 H), 7.41 (s, 1 H), 5.56 (s, 2 H), 1.28 (s, 12 H).

MS $(M + H)^+$ = 255.2

Synthesis of N-[2-chloro-5-(4, 4, 5, 5-tetramethyl-1, 3, 2-dioxaborolan-2-yl)-3-pyridyl]-N-methylsulfonyl-methanesulfonamide (4)

TEA (6.36 g, 62.86 mmol, 8.75 mL, 4 *eq*) and MsCl (6.790 g, 59.27 mmol, 4.59 mL, 3.77 *eq*) was added

dropwise to a solution of 2-chloro-5-(4, 4, 5, 5-tetramethyl-1, 3, 2-dioxaborolan-2-yl)pyridin-3-amine (4 g, 15.72 mmol, 1 *eq*) in DCM (35 mL). The mixture was purged with N_2 and the reaction mix was stirred at 0 °C for 1 h. TLC (Petroleum ether/Ethyl acetate=3:1, R_f=0.84) showed that starting material was consumed completely and a new spot was formed. The reaction mixture was concentrated in vacuum and the residue was poured into MeOH (10 mL). The mixture was stirred at 20 °C for 1 h and filtered. The filter cake was concentrated in vacuum to give a crude product. Compound N-[2-chloro-5-(4, 4, 5, 5-tetramethyl-1, 3, 2-dioxaborolan-2-yl)-3-pyridyl] -N-methylsulfonyl methanesulfonamide (4.2 g, 10.23 mmol, 65.07% yield) was obtained as a pale green solid.

^{1}H NMR (400 MHz, DMSO-d6) δ ppm 8.65 (d, *J*=1.71 Hz, 1 H), 8.26 (d, *J*=1.71 Hz, 1 H), 3.63 (s, 6 H), 1.33 (s, 12 H).

Synthesis of (R)-N-(2-chloro-5-(4-((1-phenylethyl)amino)quinazolin-6-yl)pyridin-3-yl)methane-sulfonamide) (MTX-531)

N-[2-chloro-5-(4,4,5,5-tetramethyl-1,3,2-dioxaborolan-2-yl)-3-pyridyl]-N-methylsulfonylmethanesulfonamide (200 mg, 486.97 umol, 1 eq), K_3PO_4 (310.10 mg, 1.46 mmol, 3 eq), and $Pd(PPh_3)_4$ (56.27 mg, 48.70 umol, 0.1 eq) was added to a stirred solution of 6-iodo-N-[(1R)-1-phenylethyl]quinazolin-4-amine (182.71 mg, 486.97 umol, 1 eq) in DMF (5 mL) and H2O (1 mL). The mixture was purged with N2 3 times and stirred at 100 °C for 4 h under N2. LCMS showed that the starting material was consumed completely, and the desired MS was detected. The reaction mixture was filtered and the filtrate was purified by prep-HPLC (column: Phenomenex Gemini-NX 80*40mm*3um;mobile phase: [water(10Mm NH_4HCO_3)-ACN];B%: 10%-40%,8min). (R)-N-(2-chloro-5-(4-((1-phenylethyl)amino)quinazolin-6-yl)pyridin-3-yl)methane-sulfonamide (23.2 mg, 51.11 umol, 10.49% yield, 100% purity) was obtained as a white solid.

1H NMR (400MHz, DMSO-d6) δ = 9.98 (br s, 1H), 8.85 (s, 1H), 8.79 (d, J=2.2 Hz, 1H), 8.67 (br d, J=7.7 Hz, 1H), 8.44 (s, 1H), 8.24 (d, J=2.2 Hz, 1H), 8.14 (dd, J=1.6, 8.7 Hz, 1H), 7.80 (d, J=8.7 Hz, 1H), 7.45 (d, J=7.5 Hz, 2H), 7.33 (t, J=7.6 Hz, 2H), 7.26 - 7.19 (m, 1H), 5.65 (quin, J=7.1 Hz, 1H), 3.17 (s, 3H), 1.63 (d, J=7.0 Hz, 3H).

MS (M + H)+ =454.1
