# Supplementary material for: A first-in-class selective inhibitor of EGFR and PI3K offers a single-molecule approach to targeting adaptive resistance
Source: Nat Cancer. 2024 Jul 11;5(8):1250–66. doi: 10.1038/s43018-024-00781-6 (PMC11357990; doi:10.1038/s43018-024-00781-6)
Supplement: Supplementary file 2 — Reporting Summary [file 43018_2024_781_MOESM2_ESM.pdf]

nature portfolio

Corresponding author(s): Judith S Sebolt-Leopold

Last updated by author(s): April 12, 2024

Reporting Summary

Nature Portfolio wishes to improve the reproducibility of the work that we publish. This form provides structure for consistency and transparency in reporting. For further information on Nature Portfolio policies, see our Editorial Policies and the Editorial Policy Checklist.

Statistics

For all statistical analyses, confirm that the following items are present in the figure legend, table legend, main text, or Methods section.

n/a	Confirmed	
☐	☒	The exact sample size (*n*) for each experimental group/condition, given as a discrete number and unit of measurement
☐	☒	A statement on whether measurements were taken from distinct samples or whether the same sample was measured repeatedly
☐	☒	The statistical test(s) used AND whether they are one- or two-sided<br>*Only common tests should be described solely by name; describe more complex techniques in the Methods section.*
☐	☒	A description of all covariates tested
☐	☒	A description of any assumptions or corrections, such as tests of normality and adjustment for multiple comparisons
☐	☒	A full description of the statistical parameters including central tendency (e.g. means) or other basic estimates (e.g. regression coefficient) AND variation (e.g. standard deviation) or associated estimates of uncertainty (e.g. confidence intervals)
☐	☒	For null hypothesis testing, the test statistic (e.g. *F*, *t*, *r*) with confidence intervals, effect sizes, degrees of freedom and *P* value noted<br>*Give P values as exact values whenever suitable.*
☒	☐	For Bayesian analysis, information on the choice of priors and Markov chain Monte Carlo settings
☐	☒	For hierarchical and complex designs, identification of the appropriate level for tests and full reporting of outcomes
☐	☒	Estimates of effect sizes (e.g. Cohen's *d*, Pearson's *r*), indicating how they were calculated

Our web collection on statistics for biologists contains articles on many of the points above.

Software and code

Policy information about availability of computer code

Data collection	Excel (version 2301)<br>Biotek Synergy H1 plate reader<br>Veriti 96-well Thermal Cycler<br>LC/MS 8050<br>European Synchrotron Radiation Facility, beamline ID30a1<br>SLS, beamline PXI<br>All software used was from third-party developers.

Data analysis	The following public software were used for data analysis:<br>Excel (version 2301)<br>GraphPad Prism (version 9.1.0)<br>Gen5 (v.2.01.14)<br>QuantStudio Design & Analysis Software v.1.5.2<br>Image J (v.1.53a)<br>XLfit (v.5.5.0)<br>WinNonlin (v.6.1)<br>x-ray analysis programs: AutoPROC (v.1.1.7), XDS (v.20220820), POINTLESS (v.1.12.14), AIMLESS (v.0.7.9) STARANISO (v.2.3.87), REFMAC5 (v.5.8.0352), BUSTER (v. 2.11.8), GRADE (v.1.2.15)<br>Molecular modeling program: Molegro Virtual Docker 5.5

For manuscripts utilizing custom algorithms or software that are central to the research but not yet described in published literature, software must be made available to editors and reviewers. We strongly encourage code deposition in a community repository (e.g. GitHub). See the Nature Portfolio guidelines for submitting code & software for further information.

Data

Policy information about availability of data

All manuscripts must include a data availability statement. This statement should provide the following information, where applicable:

- Accession codes, unique identifiers, or web links for publicly available datasets
- A description of any restrictions on data availability
- For clinical datasets or third party data, please ensure that the statement adheres to our policy

Co-crystal structures that support the findings of this study have been deposited to the Protein Data Bank with the accession numbers 8sc7, 8sc8, and 8sc9 and are listed in the pertinent figure legends and in Supplementary Table 5. Data supporting the findings of this study are available from the corresponding author upon reasonable request. Source Data are provided with this paper.

Research involving human participants, their data, or biological material

Policy information about studies with human participants or human data. See also policy information about sex, gender (identity/presentation), and sexual orientation and race, ethnicity and racism.

Reporting on sex and gender	No sex and gender-based analysis was performed, nor did sex or gender factor into model selection. We chose the PDX models based on tumor type, KRAS and PIK3CA mutation status, and growth characteristics.
Reporting on race, ethnicity, or other socially relevant groupings	Ethnicity information on PDX models used in this study is provided in Supplemental Data.
Population characteristics	Demographic information on PDX models used in this study is provided in Supplemental Data.
Recruitment	The majority of PDX models used in this study were obtained from the NCI Patient-Derived Models Repository (PDMR). Other models were established from colorectal cancer patients undergoing treatment at University of Michigan Hospital or MD Anderson Cancer Center following informed consent under IRB-approved protocols.
Ethics oversight	All procedures related to animal handling, care and treatment were performed under an approved protocol (PRO00010150) according to the guidelines set forth by the University of Michigan Institutional Animal Care and Use Committee (IACUC) and following the guidance of the Association for Assessment and Accreditation of Laboratory Animal Care (AAALAC). Animal models established from colorectal cancer patients required informed consent under IRB-approved protocols HUM00065489 and LAB10-0982 at University of Michigan and MD Anderson Cancer, respectively.

Note that full information on the approval of the study protocol must also be provided in the manuscript.

Field-specific reporting

Please select the one below that is the best fit for your research. If you are not sure, read the appropriate sections before making your selection.

☒ Life sciences ☐ Behavioural & social sciences ☐ Ecological, evolutionary & environmental sciences

For a reference copy of the document with all sections, see nature.com/documents/nr-reporting-summary-flat.pdf

Life sciences study design

All studies must disclose on these points even when the disclosure is negative.

Sample size	For ex vivo pharmacodynamic (PD) analysis of treated tumors, western blot samples were generated from a minimum of three individual mice. In vivo efficacy data were generated in 5-10 individual mice were treated in each group so that Standard Error of the Mean (SEM) could be calculated for each treatment group and is indicated with error bars. No statistical methods were used to pre-determine group sizes, but ours were similar to those reported in previous publications cited in the Methods section. For in vivo efficacy studies, n = 5-10 was sufficient

to provide statistical significance in all experiments generating robust activity, while balancing the cost and housing requirements for mice (5 mice per cage). For PD studies, n=3/time point provided sufficient replicates to evaluate heterogeneity of response to treatment, while balancing cost and the number of mice required to show consistency of outcome in multiple replicate studies. For in vitro assays, sample size was determined based on observation of consistent data in biological replicates, and the number of experimental samples that could reasonably fit in a multi-well plate.

Data exclusions	No animals or data points were excluded from analyses. In efficacy studies, tumor growth curves are not shown after loss of all vehicle treated animals. However, drug treated animals continued to be dosed to capture impact on survival.
Replication	All in vitro cellular data sets were duplicated to confirm replication of data outcome. We provide information in the figure legends pertaining to the number of times in vitro experiments were performed when representative data are shown. Replication of data outcome was confirmed in each instance. Replicate in vivo studies (efficacy and PD) were generated for some but not all models. However, internal controls were included in each study to confirm outcomes. For in vivo studies that were replicated, the outcomes were in agreement.
Randomization	Sample randomization is not relevant to the in vitro studies presented as no relevant differing covariates could be identified. All in vitro samples were processed and measured in a consistent manner. Animals were enrolled and randomized onto study based on tumor measurements on a specific day post-inoculation. Groups were established so that the mean tumor size of each group was similar across all groups prior to treatment initiation Body weights were measured to confirm animal health prior to the start of the study, but were not used as a covariate for randomization.
Blinding	Investigators were not blinded in this preclinical study. Since tumor models can vary in tumor growth, investigators were not blinded during randomization to ensure that tumors of equal volume were distributed equally between treatment groups. Furthermore, treatment was not blinded, since mice were dosed daily and at the same time as tumor measurements were obtained.

Reporting for specific materials, systems and methods

We require information from authors about some types of materials, experimental systems and methods used in many studies. Here, indicate whether each material, system or method listed is relevant to your study. If you are not sure if a list item applies to your research, read the appropriate section before selecting a response.

Materials & experimental systems

n/a	Involved in the study	
☐	☒	Antibodies
☐	☒	Eukaryotic cell lines
☒	☐	Palaeontology and archaeology
☐	☒	Animals and other organisms
☒	☐	Clinical data
☒	☐	Dual use research of concern
☒	☐	Plants

Methods

n/a	Involved in the study	
☒	☐	ChIP-seq
☒	☐	Flow cytometry
☒	☐	MRI-based neuroimaging

Antibodies

Antibodies used	The following primary antibodies were obtained from Cell Signaling Technologies and used at 1:1000 dilution, unless otherwise noted: anti-p-EGFR (tyr1068) #3777, anti-p-EGFR (tyr1068) #2234, anti-EGFR #2646 (1:10,000), anti-p-AKT (thr308) #13038, anti-p-AKT (ser473) #4060, anti-AKT #9272 (1:5000), anti-pS6 (ser235/236) # 4857, anti-S6 #2217 (1:10,000), anti-p-PRAS40 (thr246) #2997, anti-PRAS40 #2691 (1:10,000), anti-p-4E-BP1 (ser65) #9451), anti-4E-BP1 #9644, anti-p-p70 S6K (thr389) #97596, anti-p70 S6K #9202, anti-PPARγ #2443, anti-cleaved PARP #9541. Anti-β-actin HRP conjugated #197277 (1:10,000) and anti-vinculin #129002 (1:10,000) were obtained from Abcam. For the secondary antibody, peroxidase-conjugated AffiniPure goat anti-rabbit IgG (1:10,000) was obtained from Jackson ImmunoResearch Laboratories, #111-035-003).
Validation	All antibodies were validated by the manufacturers (Cell Signaling Technologies or Abcam) and have been extensively used in published studies cited on the manufacturer websites, as exemplified below:<br>Anti-Phospho-EGFR – Validated in "Tulpule et al., Cell (2021); Ki et al., Nature Comm (2021); Britain et al., J Biol Chem (2020); Galini et al., Nature (2023)"<br>Anti-EGFR – Validated in "Abouantoun, et al., Mol Cancer Ther (2009); Henjes, et al., Oncogenesis (2012)"<br>Anti-phospho-AKT –Validated in "Lu, et. al., J Clin Invest (2014); Izquierdo et al., Cancer Discov (2022)"<br>Anti-AKT – "Westbrook, et. al., Cell (2005); Oh, et. al., J Biol Chem (2005)"<br>Anti-phospho-S6 – Validated in "Wang, et. al., Nat Comm (2017); Vujic, et. al., Oncotarget (2014)"<br>Anti-S6 – Validated in "Westbrook, et. al., Cell (2005); Lee, et. al., Cancer Discov (2013)<br>Anti-phospho-PRAS40 - Validated in "Cassell, et. al., Neoplasia (2012); Yi, et. al., Oncotarget (2013)"<br>Anti-PRAS40 – Validated in "Deng et al., J Biol Chem (2022); Cooper, et. al., Cancer Res (2017)"<br>Anti-phospho-4E-BP1 – Validated in "Szaflarski, et. al., Oncotarget (2016); Salas, et. al., Oncogene (2016)"<br>Anti-4E-BP1 – Validated in Hägerstrand, et. al., Cancer Discov (2013); Wang, et. al., Cancer Res (2013)"<br>Anti-phospho-p70 S6K – Validated in "Tan, et. al., Front NeuroSci (2019); Wang, et. al., Int J Med Sci (2021)"<br>Anti-p70 S6K – Validated in "Lin, et. al., J Immunol (2009); Wang, et. al., J Biol Chem (2012)"<br>Anti-PPARgamma – Validated in "Liu, et. al., J Clin Invest (2012); Soofi, et. al., J Biol Chem (2017)"<br>Anti-cleaved PARP – Validated in "Mungrue, et. al., J Immunol (2009); Monick, et. al., J Biol Chem (2005)"<br>Anti-beta-actin HRP – Validated in "Zafar et al., Biochemistry (2018); Schröder et al., Front Immunol (2019)"<br>Anti-vinculin – Validated in "Zhang et al., Cancer Res (2020); Sorrentino et al., Gastroenterology (2020)"

AffiniPure goat anti-rabbit IgG – Validated in "Mizuno et al., J Biol Chem (2022); Nunes-Santos et al., Nat Comm (2023)"

Eukaryotic cell lines

Policy information about cell lines and Sex and Gender in Research

Cell line source(s)	CAL-27 (ACC 446) and CAL-33 (ACC 447) were obtained from Leibniz Institute DSMZ German Collection of Microoganisms and Cell Cultures. BICR 16 (06 31001) and BICR 56 (060031002) cell lines were obtained from the European Collection of Authenticated Cell cultures through MilliporeSigma. MIA PaCa-2 (CRL-1420), Detroit-562 (CCL-138), and 3T3-L1 (CL-173) cell lines were obtained from the American Type Culture Collection. HEK 293H cells, used by Thermo Fisher to engineer a beta lactamase reporter PPARgamma assay, can be obtained from Gibco (11631017). The mouse OSCC cell line MOC1 (EWL001--FP) was obtained from KeraFast. KPC tumors originated from 65 671 cells (FVB/N strain), which were obtained from Marina Pasca di Magliano (University of Michigan).
Authentication	All cell lines were STR profiled for authenticity.
Mycoplasma contamination	All cell lines were routinely tested for mycoplasma and tested negative throughout the course of these studies.
Commonly misidentified lines (See ICLAC register)	No commonly misidentified cell lines were used.

Animals and other research organisms

Policy information about studies involving animals; ARRIVE guidelines recommended for reporting animal research, and Sex and Gender in Research

Laboratory animals	6-8 week old, female, athymic NCr-Foxn1 (nu/nu), 6-8 week old, female CIEA NOG mice, 6-8 weeks old, female inbred FVB mice.
Wild animals	This study did not involve wild animals.
Reporting on sex	Only female mice were used in these studies due to animal housing requirements. Sex was not considered in the study design, since these studies center around cancer signaling and drug targets that generally do not require sex-based consideration.
Field-collected samples	This study did not involve samples collected from the field.
Ethics oversight	All studies were conducted with an approved protocol (PRO00010150) and in compliance with guidelines set forth by the University of Michigan Institutional Animal Care and Use Committee (IACUC) and following the guidance of the Association for Assessment and Accreditation of Laboratory Animal Care (AAALAC). The maximal tumor size allowed by this protocol was 2000 mm3. This maximal tumor size was not exceeded for any animals used in these experiments.

Note that full information on the approval of the study protocol must also be provided in the manuscript.
