# Supplementary material for: A first-in-class selective inhibitor of EGFR and PI3K offers a single-molecule approach to targeting adaptive resistance
Source: Nat Cancer. 2024 Jul 11;5(8):1250–66. doi: 10.1038/s43018-024-00781-6 (PMC11357990; doi:10.1038/s43018-024-00781-6)

Figure 2a: MTX-531 titration in CAL33 cells

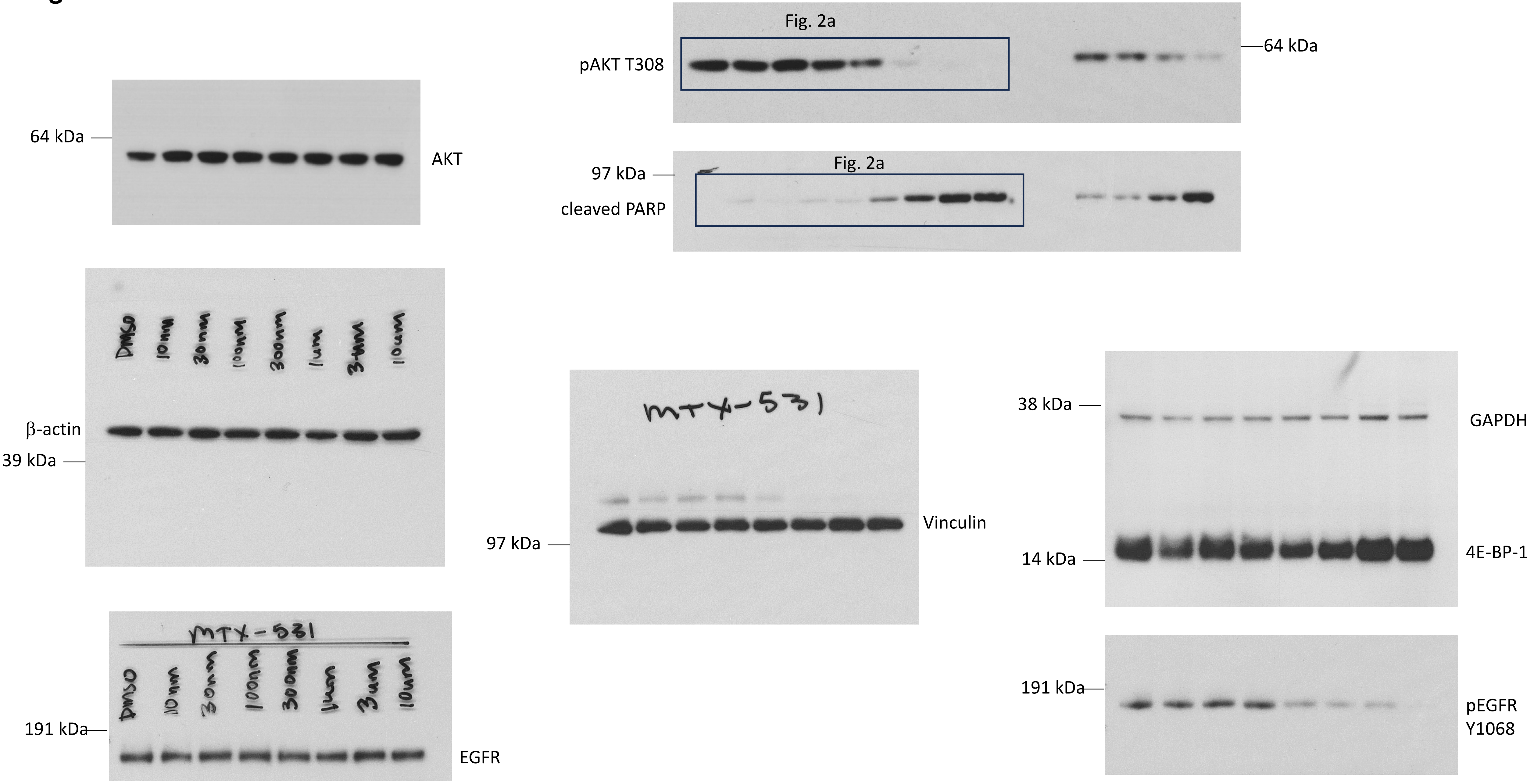

Figure 2a cont'd: MTX-531 titration in CAL33 cells

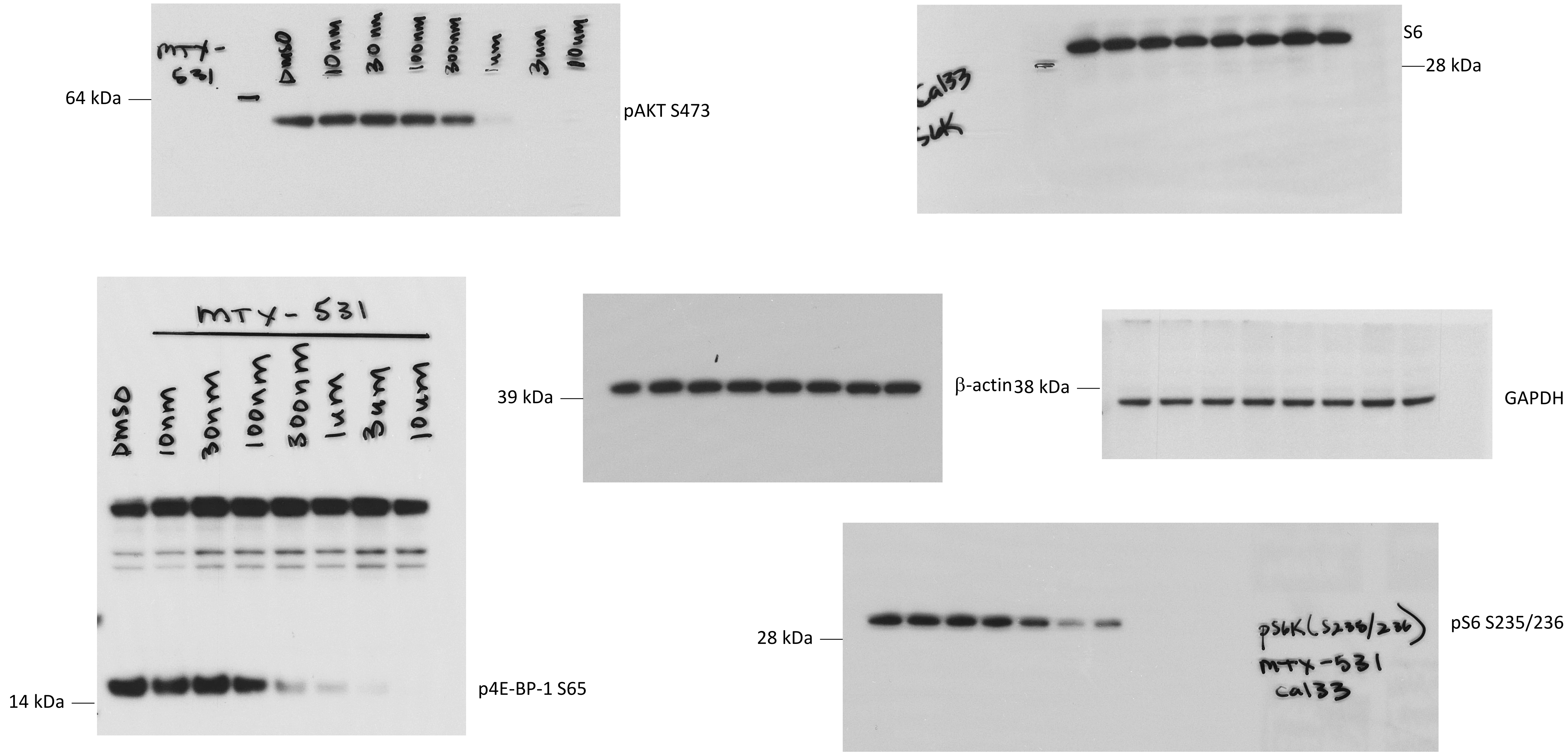

Figure 2b: MTX-531 100 mg/kg time course PD in CAL33

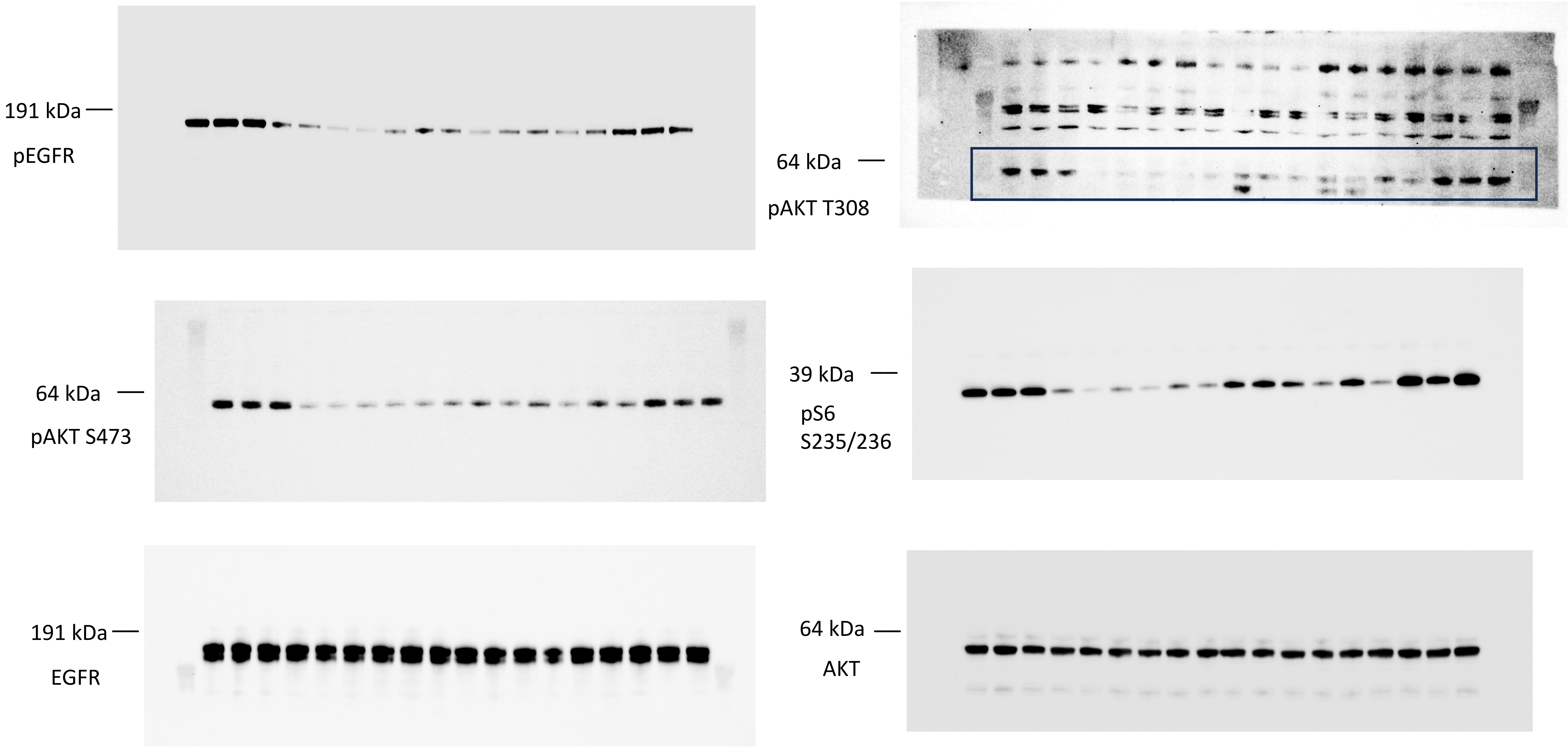

Figure 2b cont'd: MTX-531 100 mg/kg time course PD in CAL33

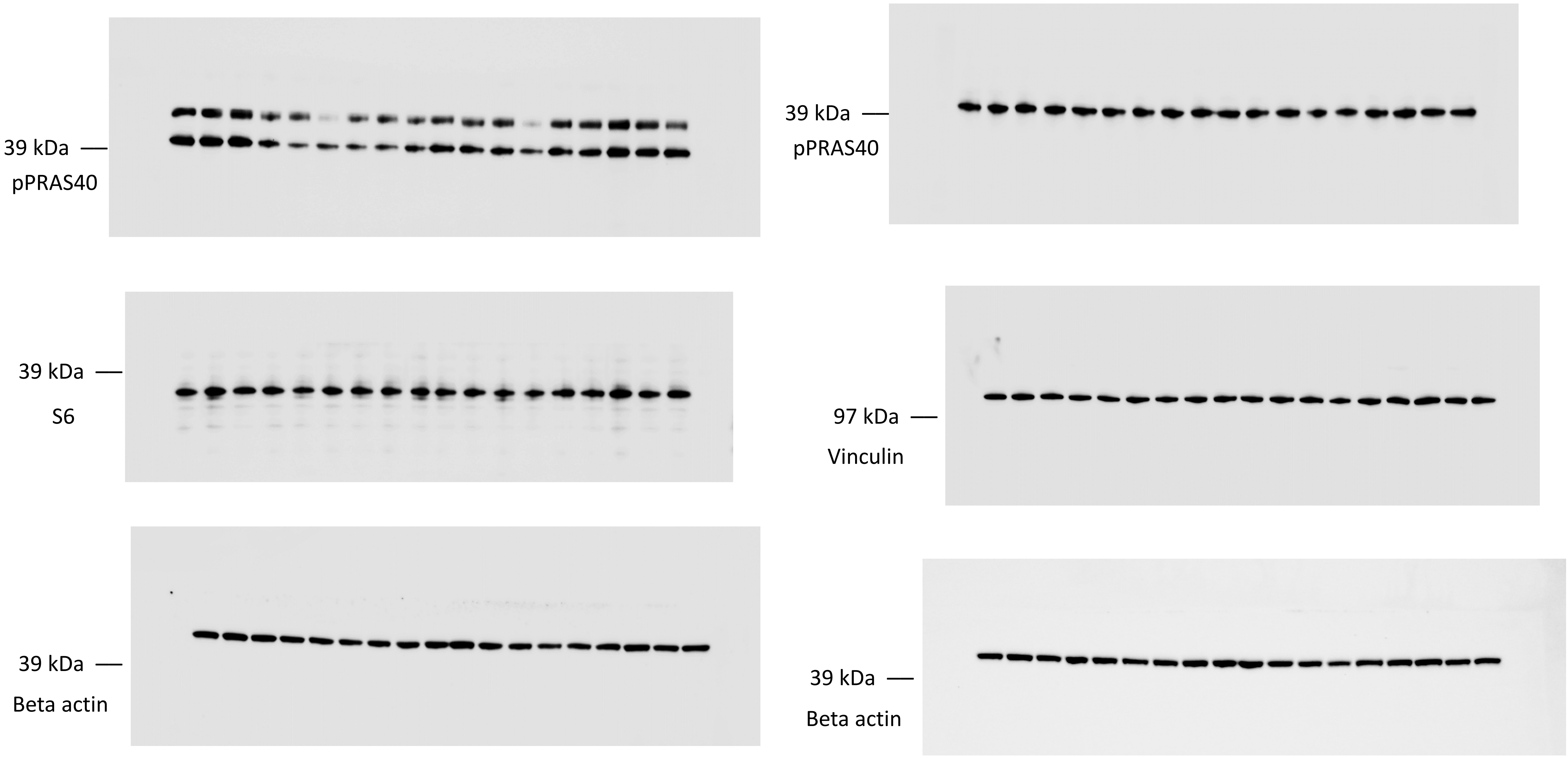

Supplement: Supplementary file 6 — Unprocessed western blots. [file 43018_2024_781_MOESM6_ESM.pdf]
