# Supplementary figures and images for: A first-in-class selective inhibitor of EGFR and PI3K offers a single-molecule approach to targeting adaptive resistance
Source: Nat Cancer. 2024 Jul 11;5(8):1250–66. doi: 10.1038/s43018-024-00781-6 (PMC11357990; doi:10.1038/s43018-024-00781-6)

Figure 3a: MTX-531 2-hour PD in 848979

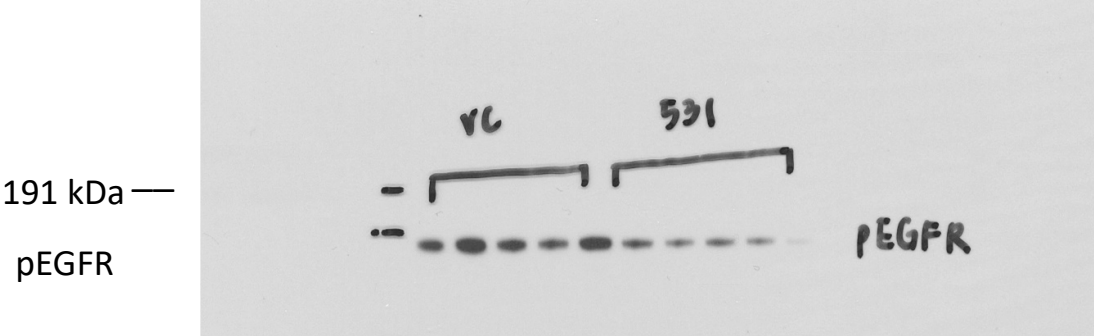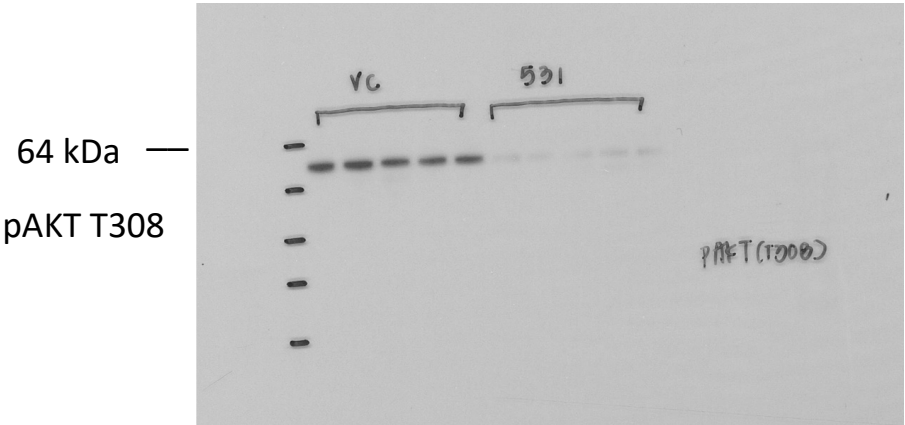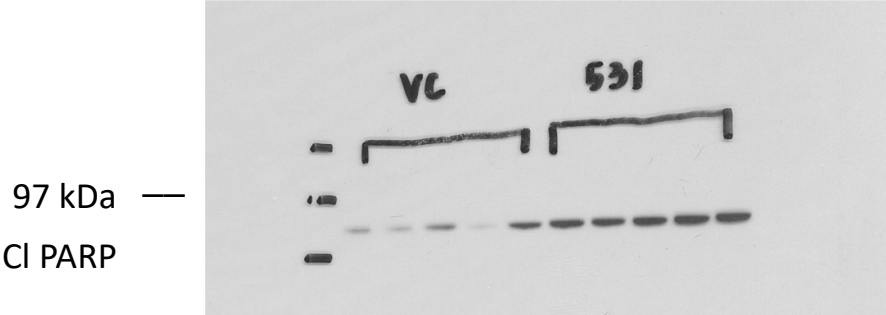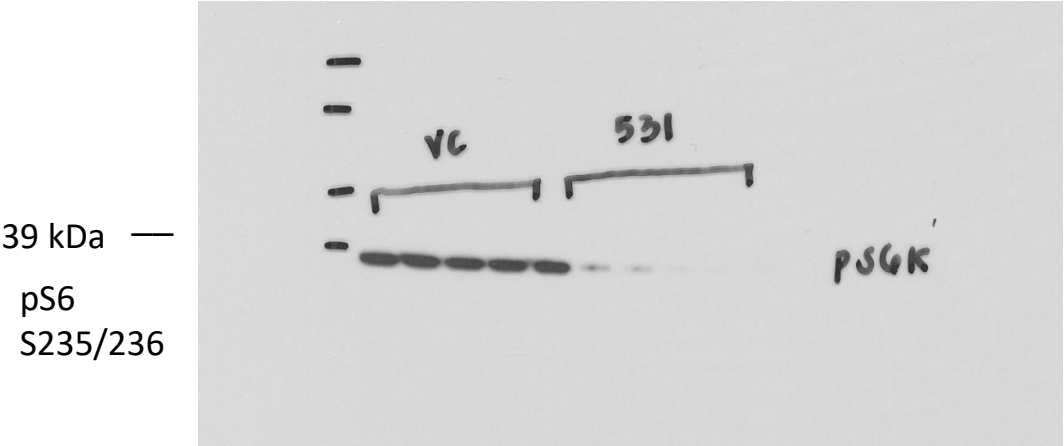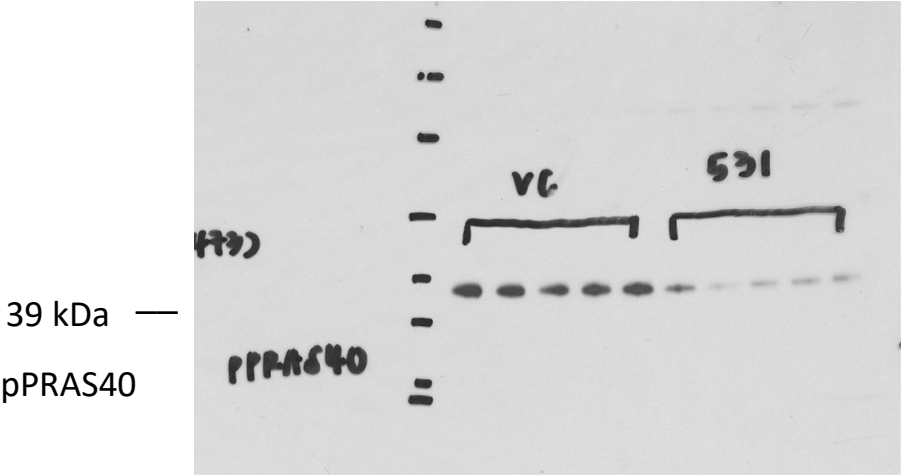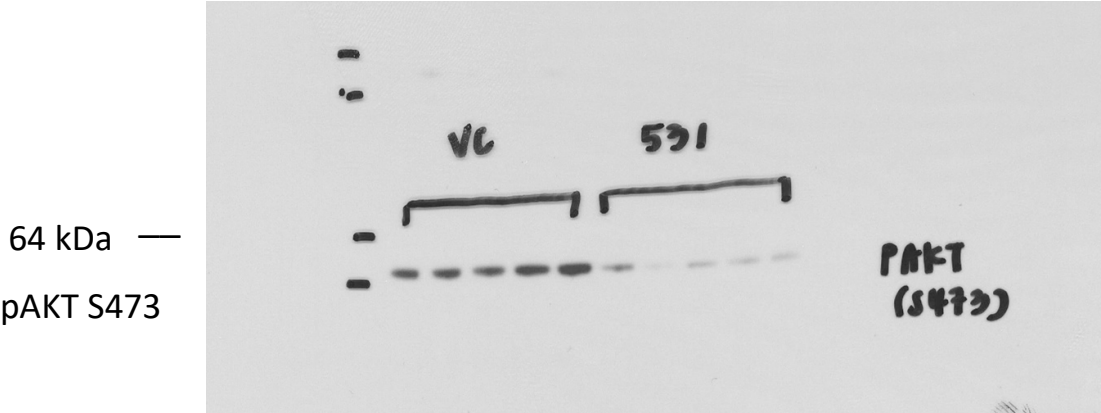

Figure 3a cont'd: MTX-531 2-hour PD in 848979

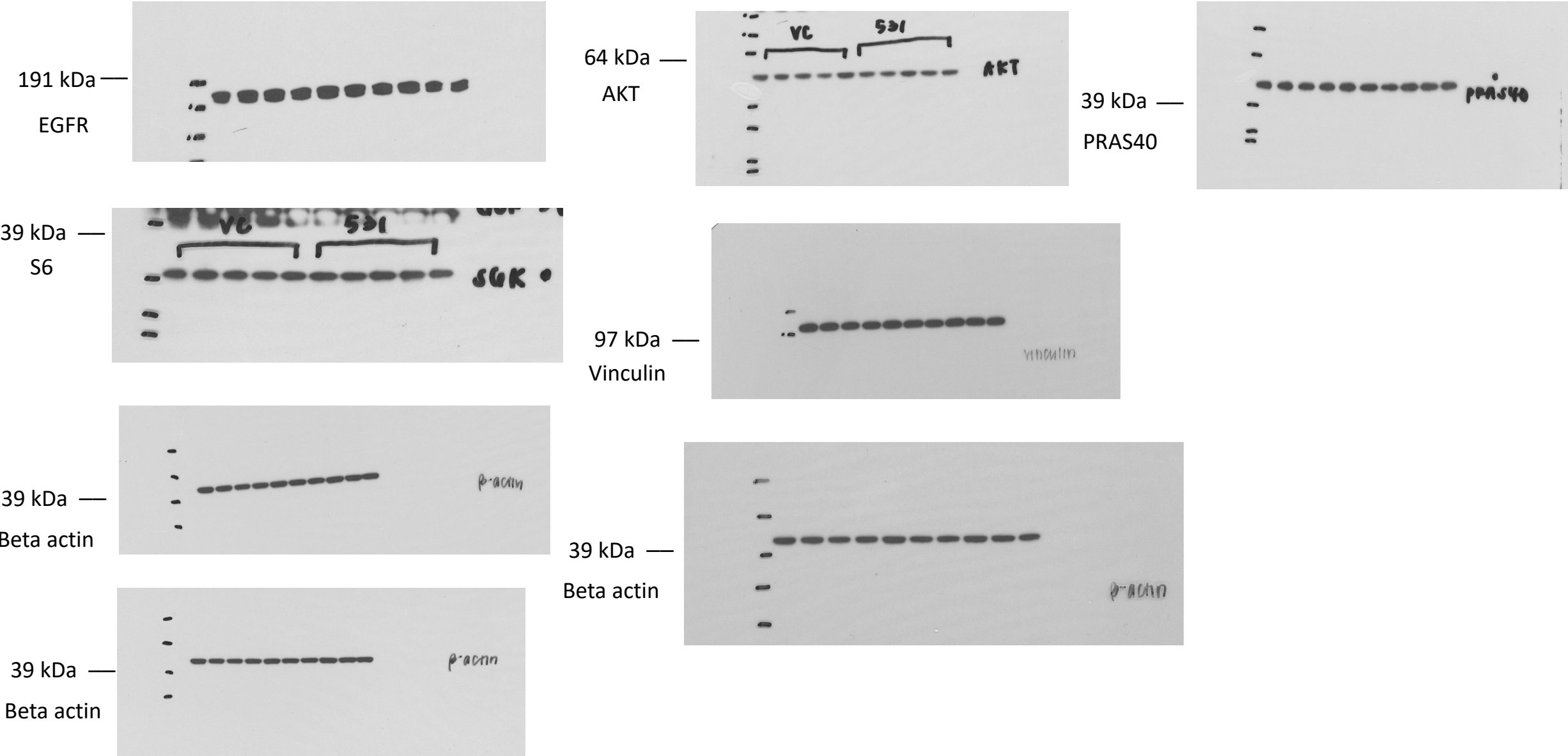

Supplement: Supplementary file 9 — Unprocessed western blots. [file 43018_2024_781_MOESM9_ESM.pdf]

Figure 4b: MTX-531 + trametinib 5-day PD in UM-CRC 14-929

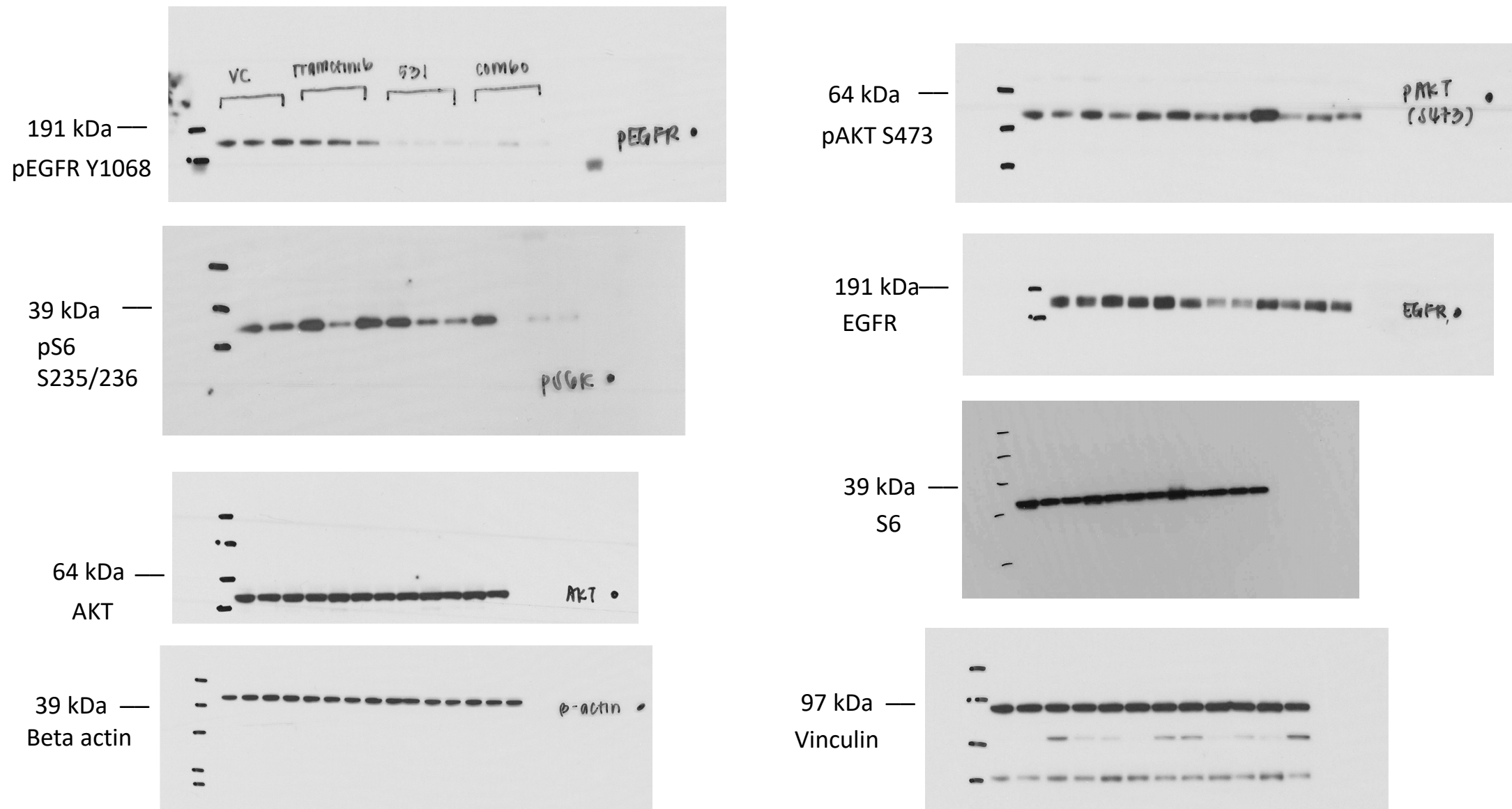

Supplement: Supplementary file 11 — Unprocessed western blots. [file 43018_2024_781_MOESM11_ESM.pdf]

Figure 7c: MTX-531 vs Rosiglitazone in 3T3 L1 Adipocytes

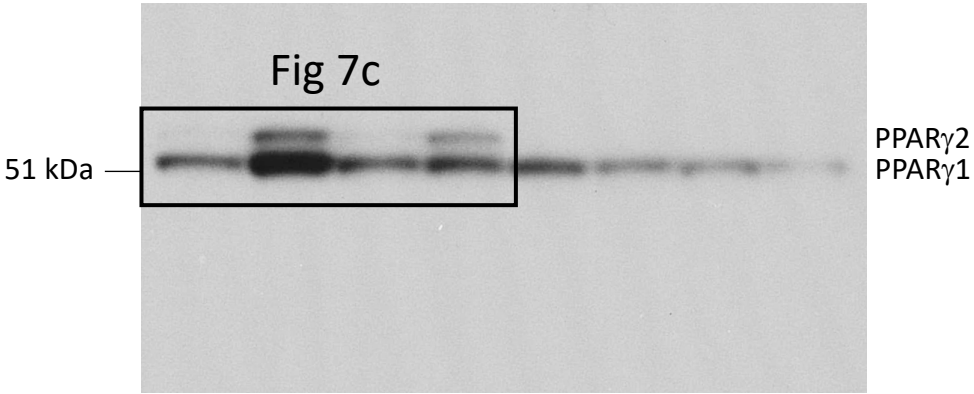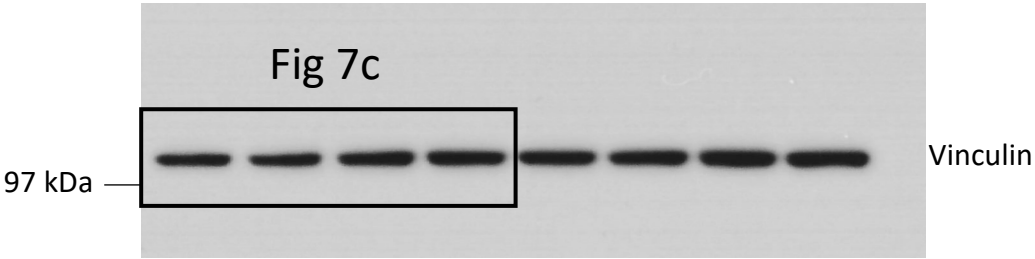

Supplement: Supplementary file 16 — Unprocessed western blots. [file 43018_2024_781_MOESM16_ESM.pdf]

Extended Data Figure 1e: MELK Inhibition in CAL-33 Cells (MTX-531 vs OTSSP)

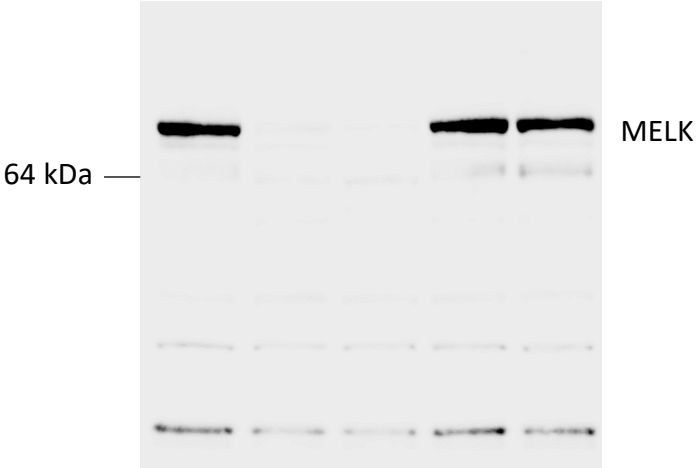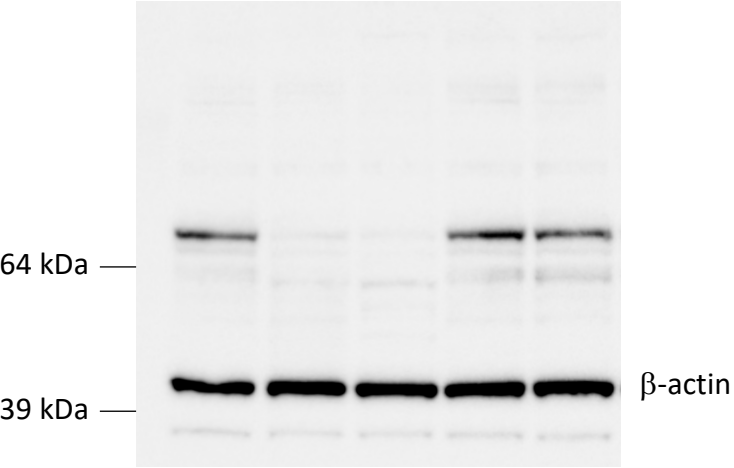

Supplement: Supplementary file 18 — Unprocessed western blots. [file 43018_2024_781_MOESM18_ESM.pdf]
