# Supplementary material for: A first-in-class selective inhibitor of EGFR and PI3K offers a single-molecule approach to targeting adaptive resistance
Source: Nat Cancer. 2024 Jul 11;5(8):1250–66. doi: 10.1038/s43018-024-00781-6 (PMC11357990; doi:10.1038/s43018-024-00781-6)

Figure 5b: MTX-531 + Sotorasib Combination 2-hour PD in B8324

64 kDa —
pAKT T308

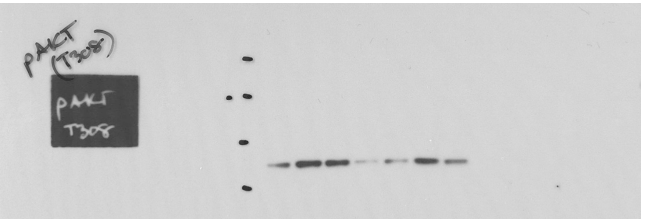

64 kDa —
pAKT S473

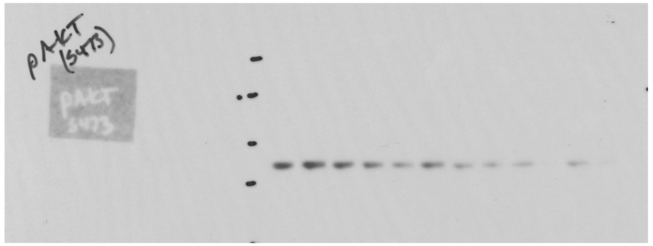

64 kDa —
p-pS6K T389

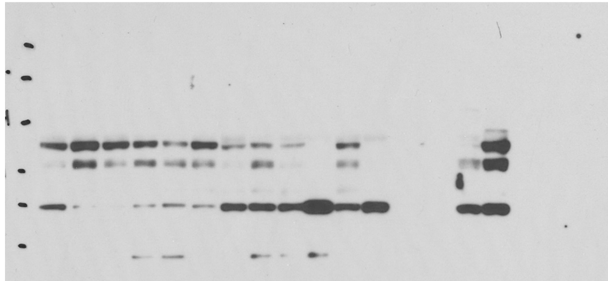

39 kDa —
pS6
S235/236

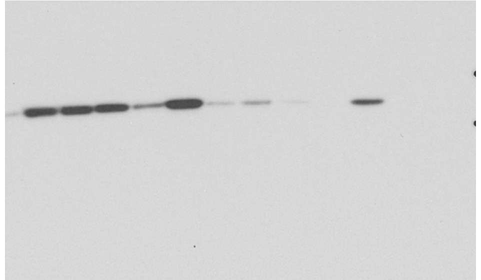

64 kDa —
AKT

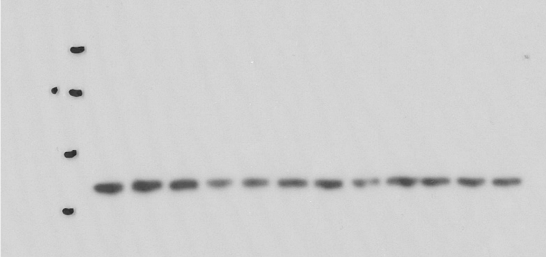

97 kDa —
CL PARP

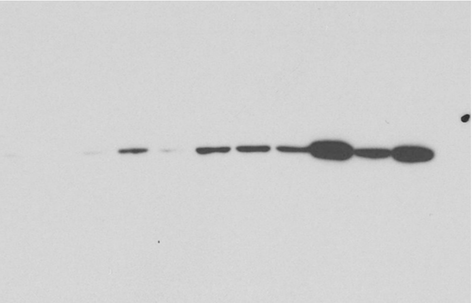

39 kDa —
S6

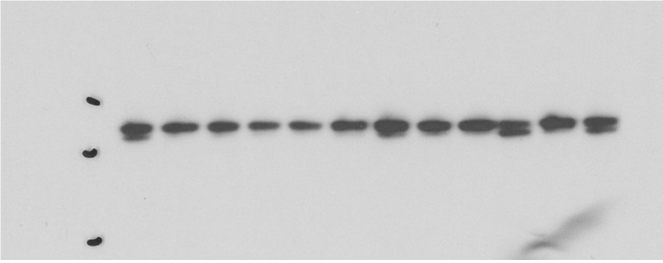

64 kDa —
pS6K

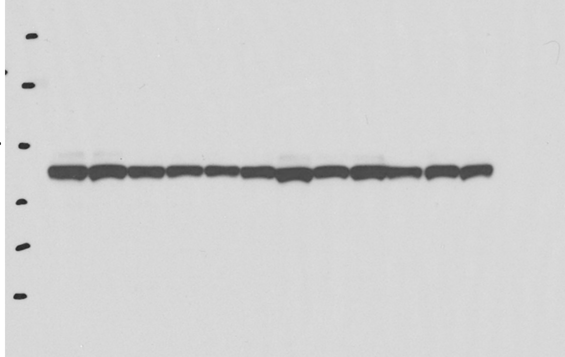

Figure 5b cont'd: MTX-531 + Sotorasib Combination 2-hour PD in B8324

97 kDa —
Vinculin

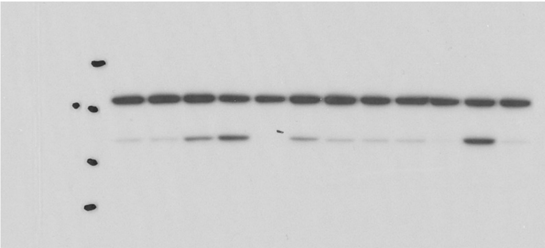

97 kDa —
Vinculin

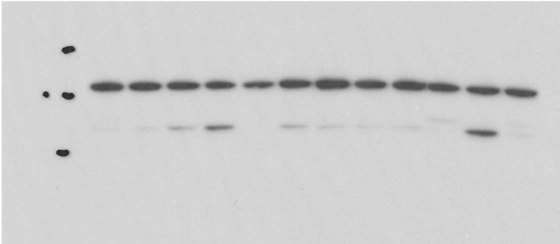

97 kDa —
Vinculin

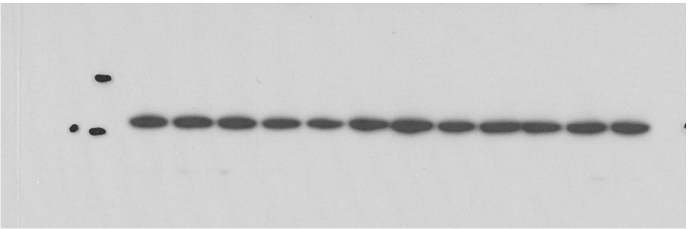

97 kDa —
Vinculin

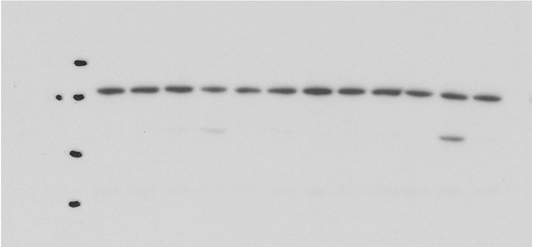

191 kDa —
pEGFR Y1068

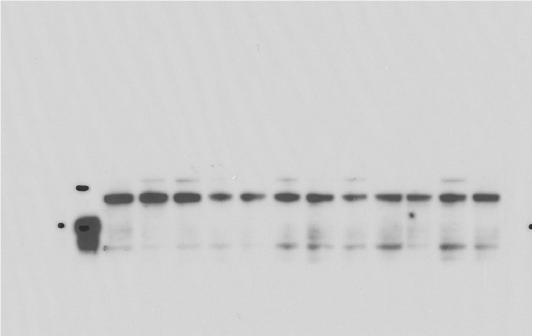

191 kDa —
EGFR

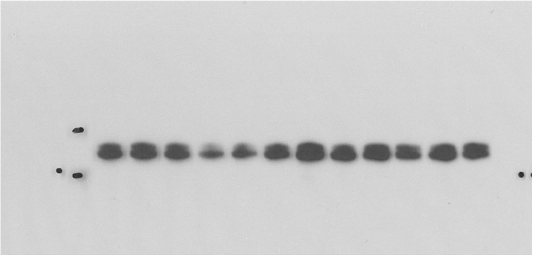

Supplement: Supplementary file 13 — Unprocessed western blots. [file 43018_2024_781_MOESM13_ESM.pdf]
