# Supplementary material for: A first-in-class selective inhibitor of EGFR and PI3K offers a single-molecule approach to targeting adaptive resistance
Source: Nat Cancer. 2024 Jul 11;5(8):1250–66. doi: 10.1038/s43018-024-00781-6 (PMC11357990; doi:10.1038/s43018-024-00781-6)

Extended Data Figure 2a: MTX-531 5- and 15-min. time course in CAL-33 cells

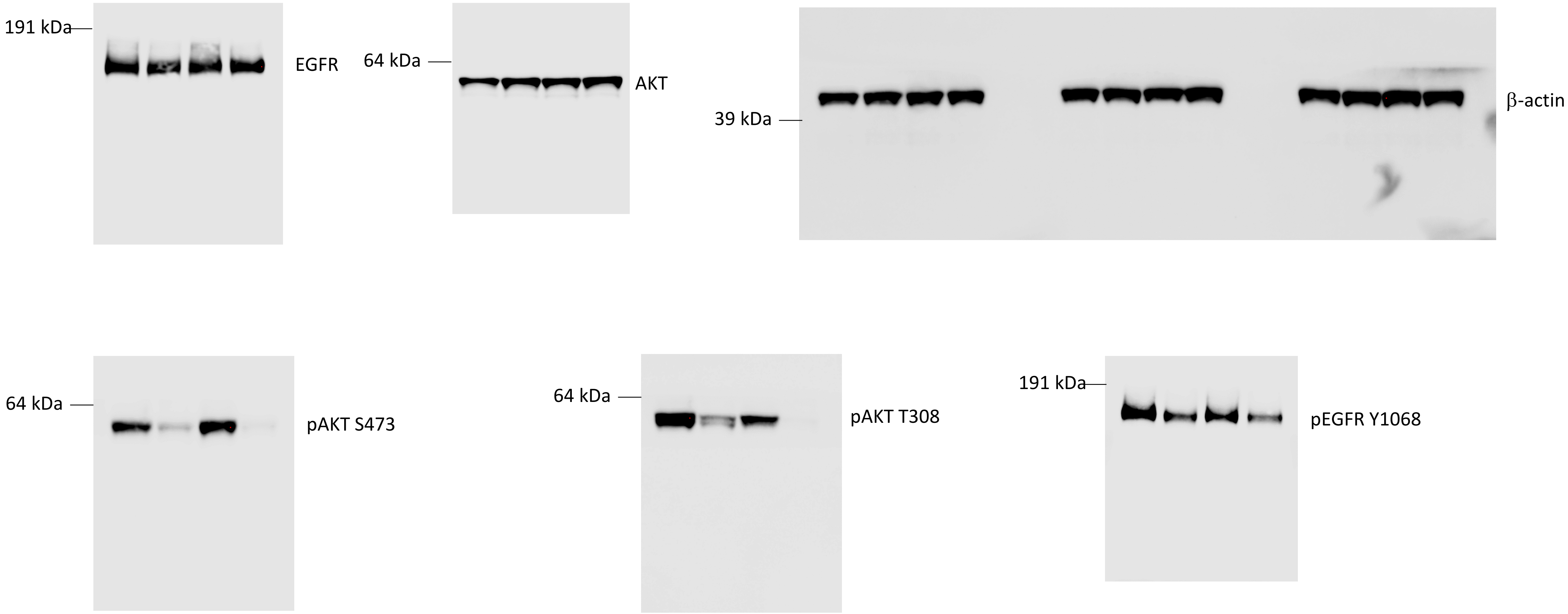

Extended Data Figure 2b: Alpelisib and Erlotinib titrations in CAL-33 cells

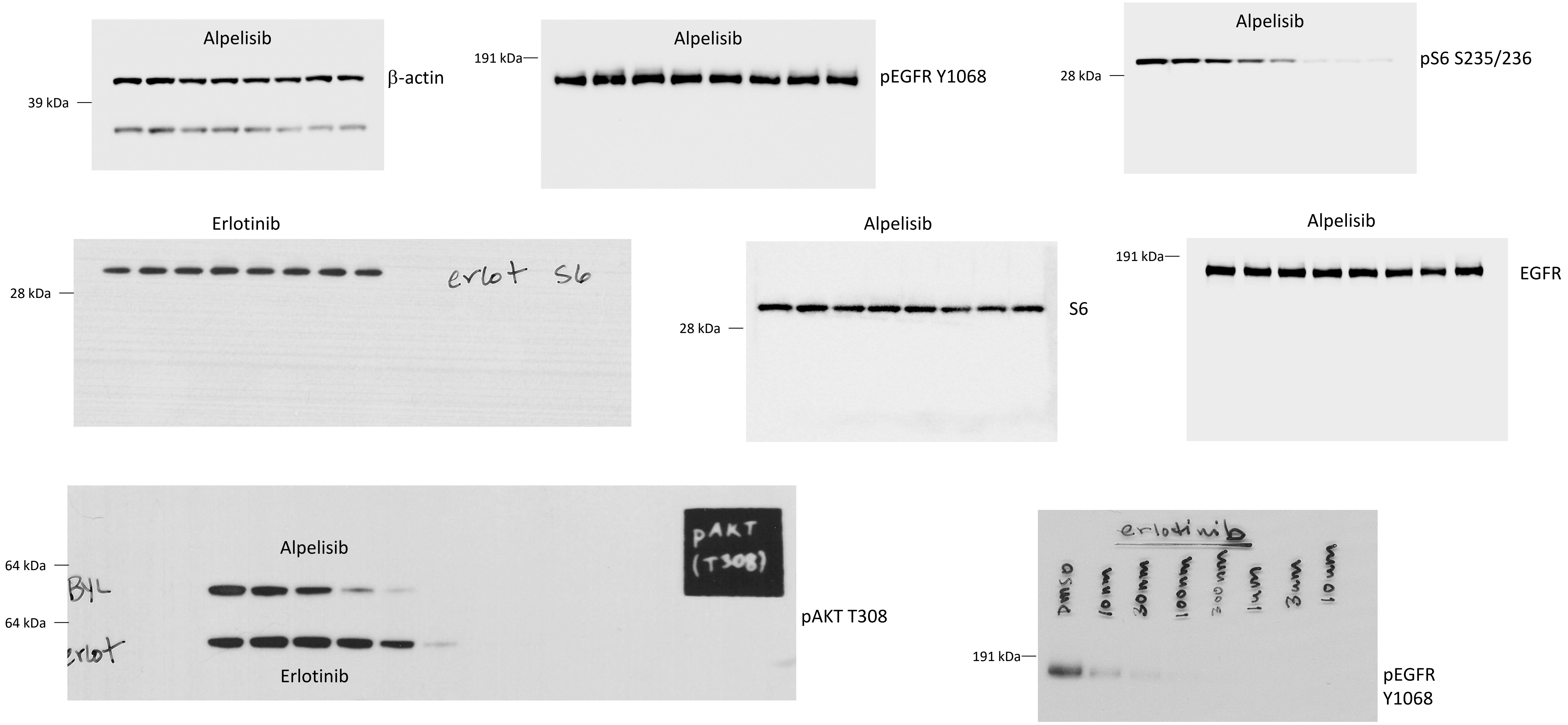

Extended Data Figure 2b cont'd: Alpelisib and Erlotinib titrations in CAL-33 cells

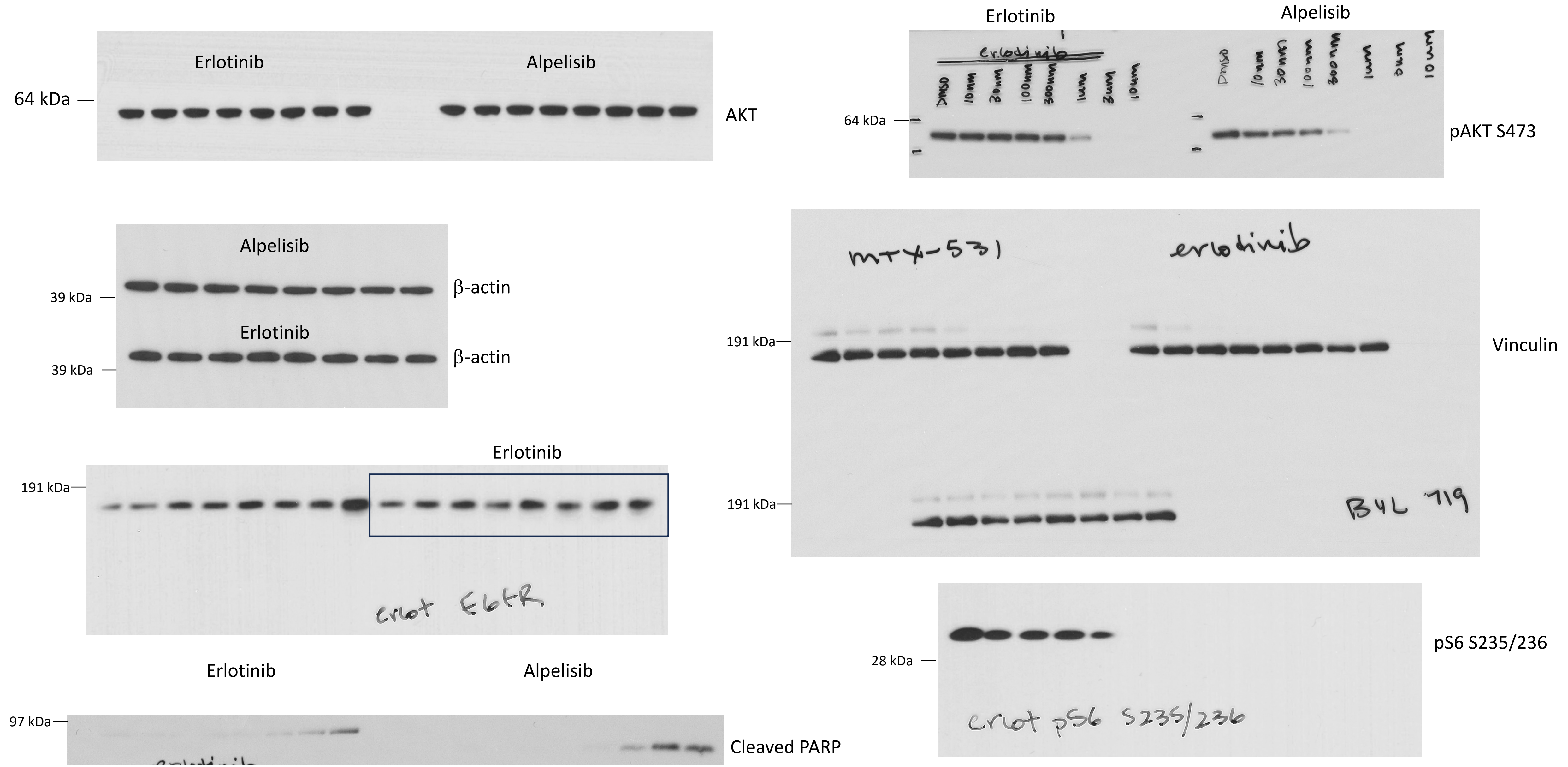

Extended Data Figure 2c: MTX-531 titration in CAL-27 cells

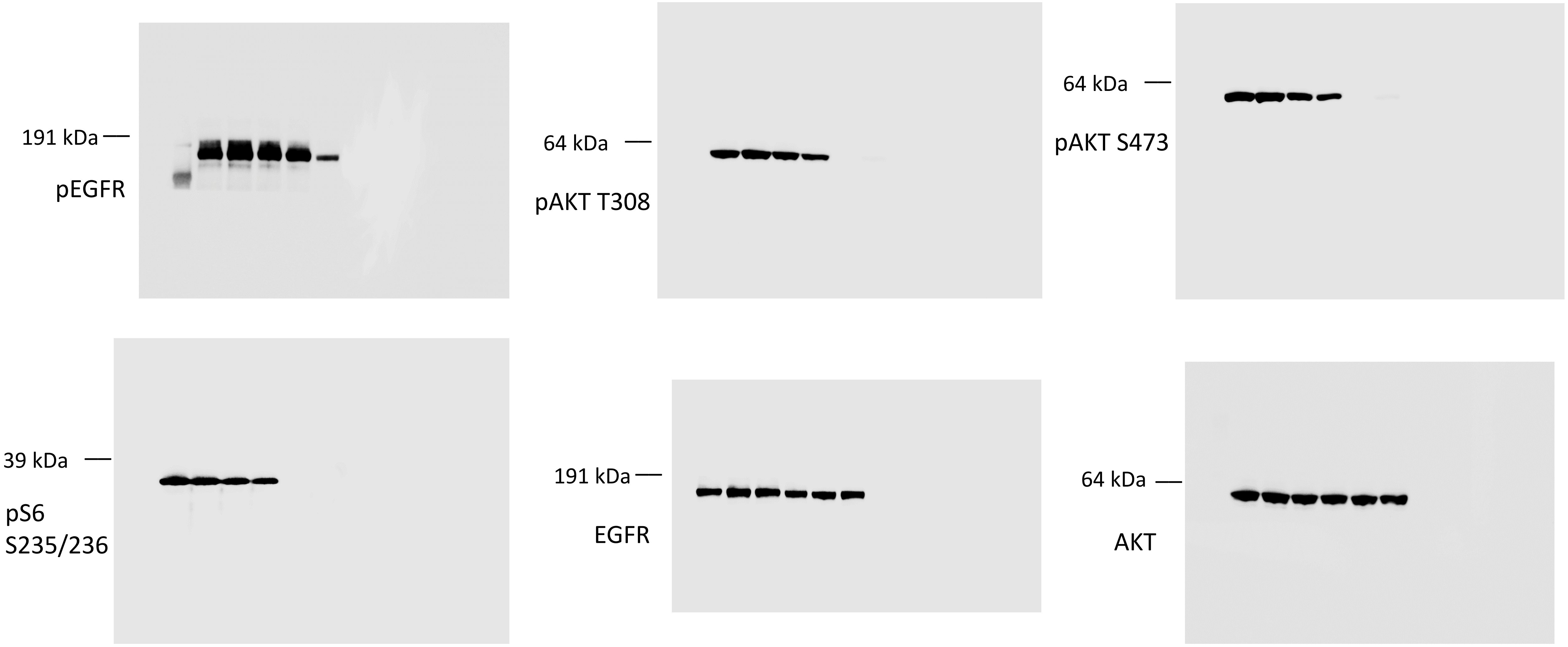

Extended Data Figure 2c cont'd: MTX-531 titration in CAL-27 cells

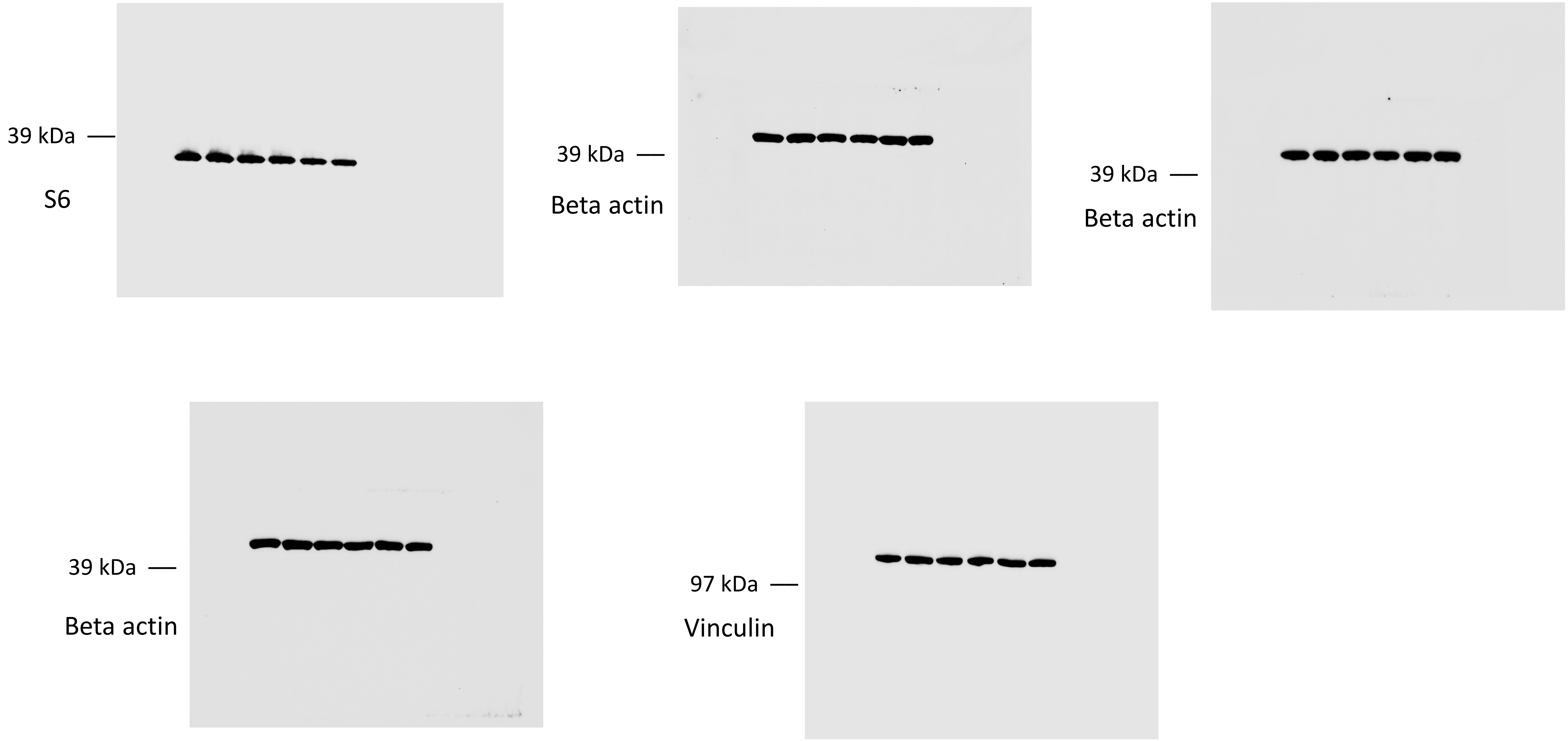

Extended Data Figure 2c cont'd: MTX-531 titration in MOC1 cells

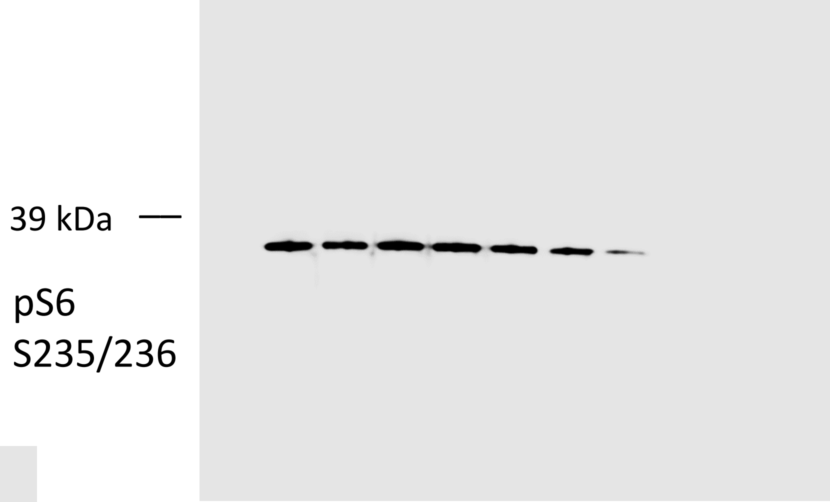

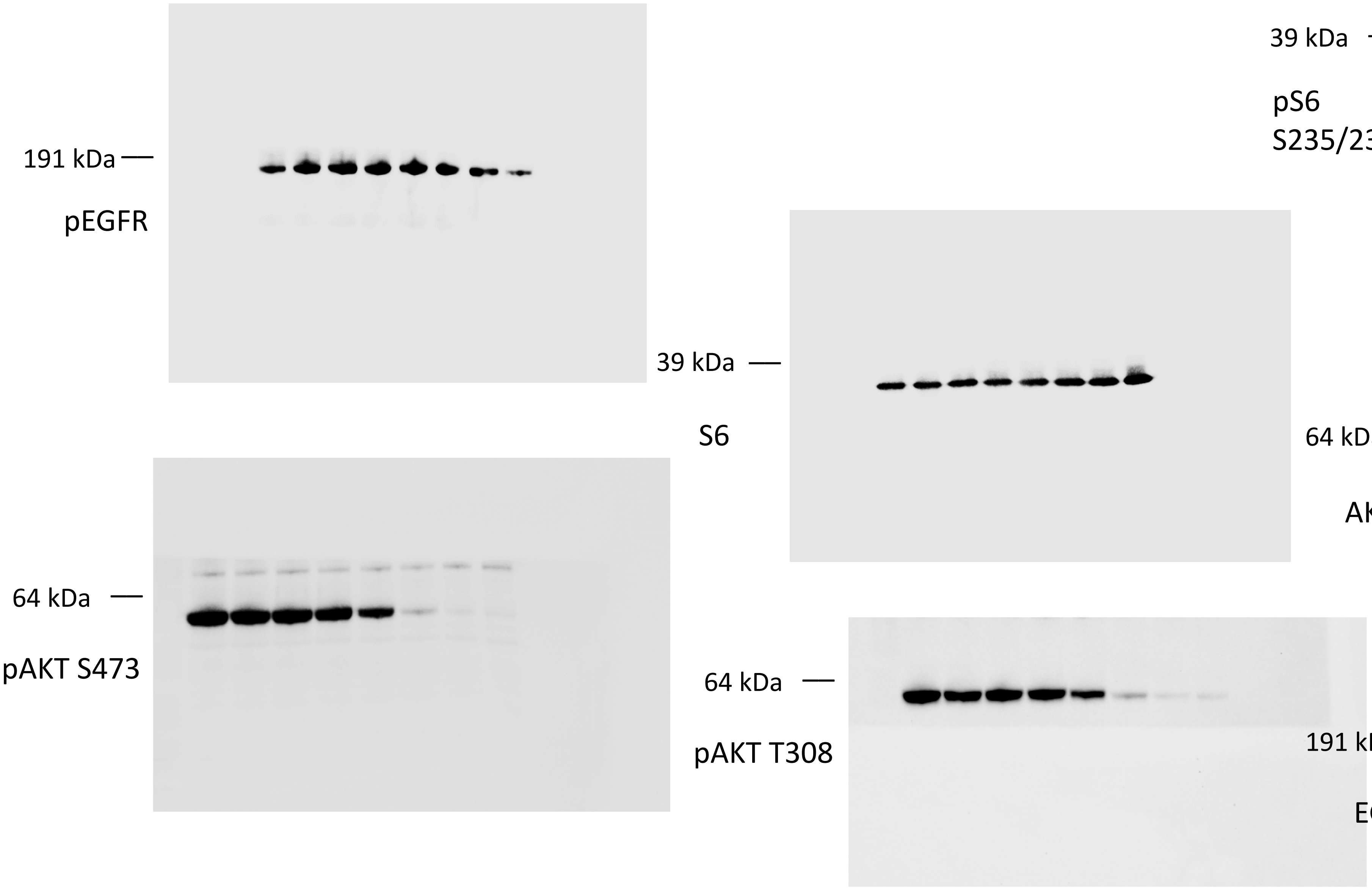

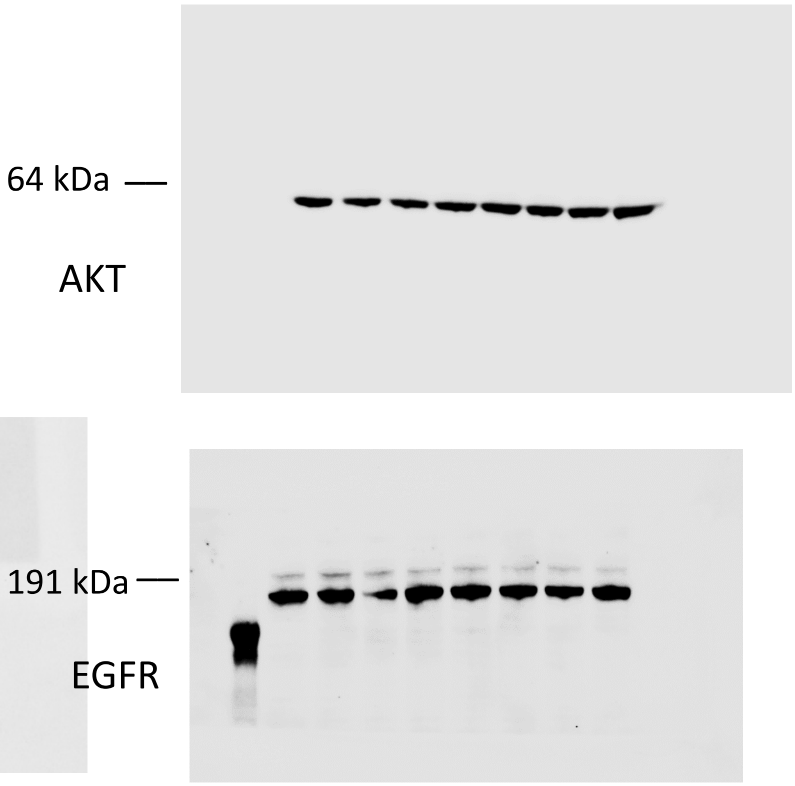

Extended Data Figure 2c cont'd: MTX-531 titration in MOC1 cells

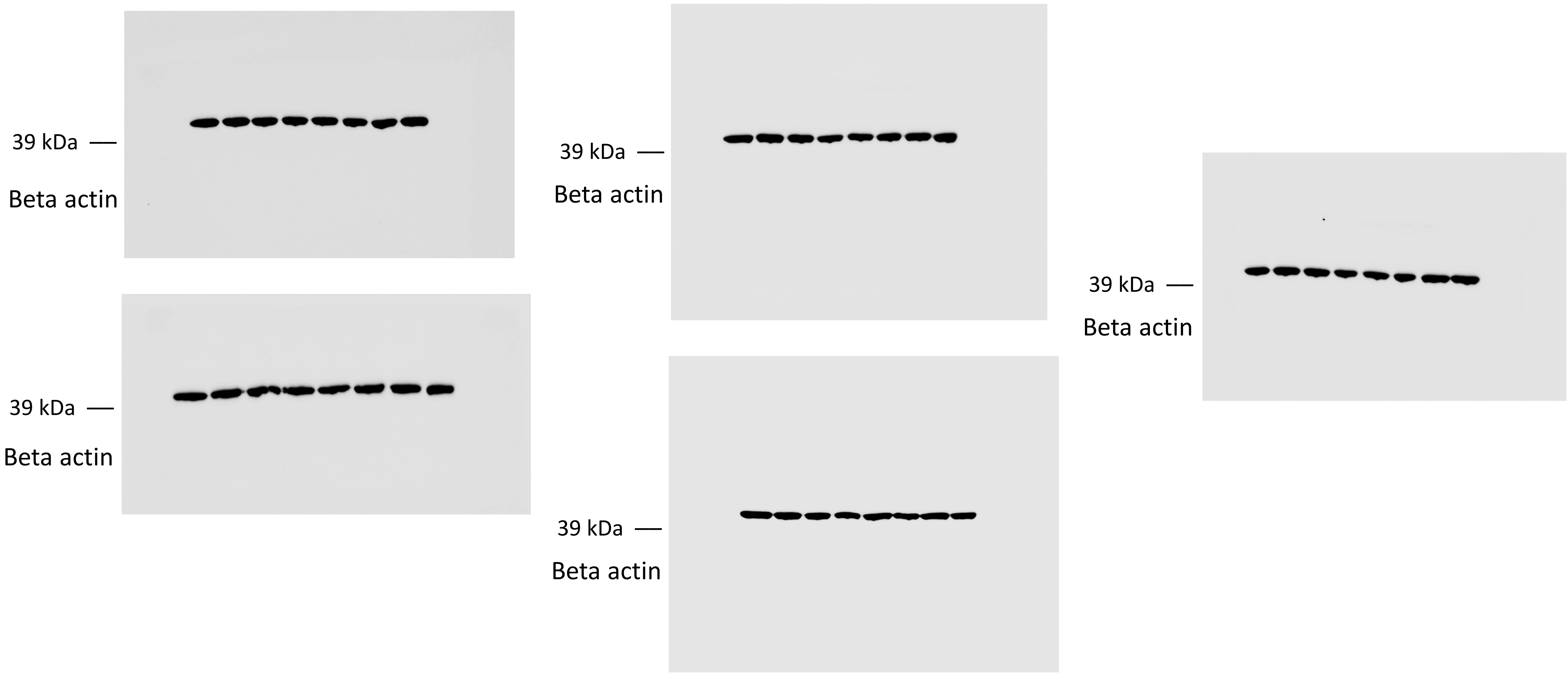

Extended Data Figure 2c cont'd: MTX-531 titration in BICR 16 cells

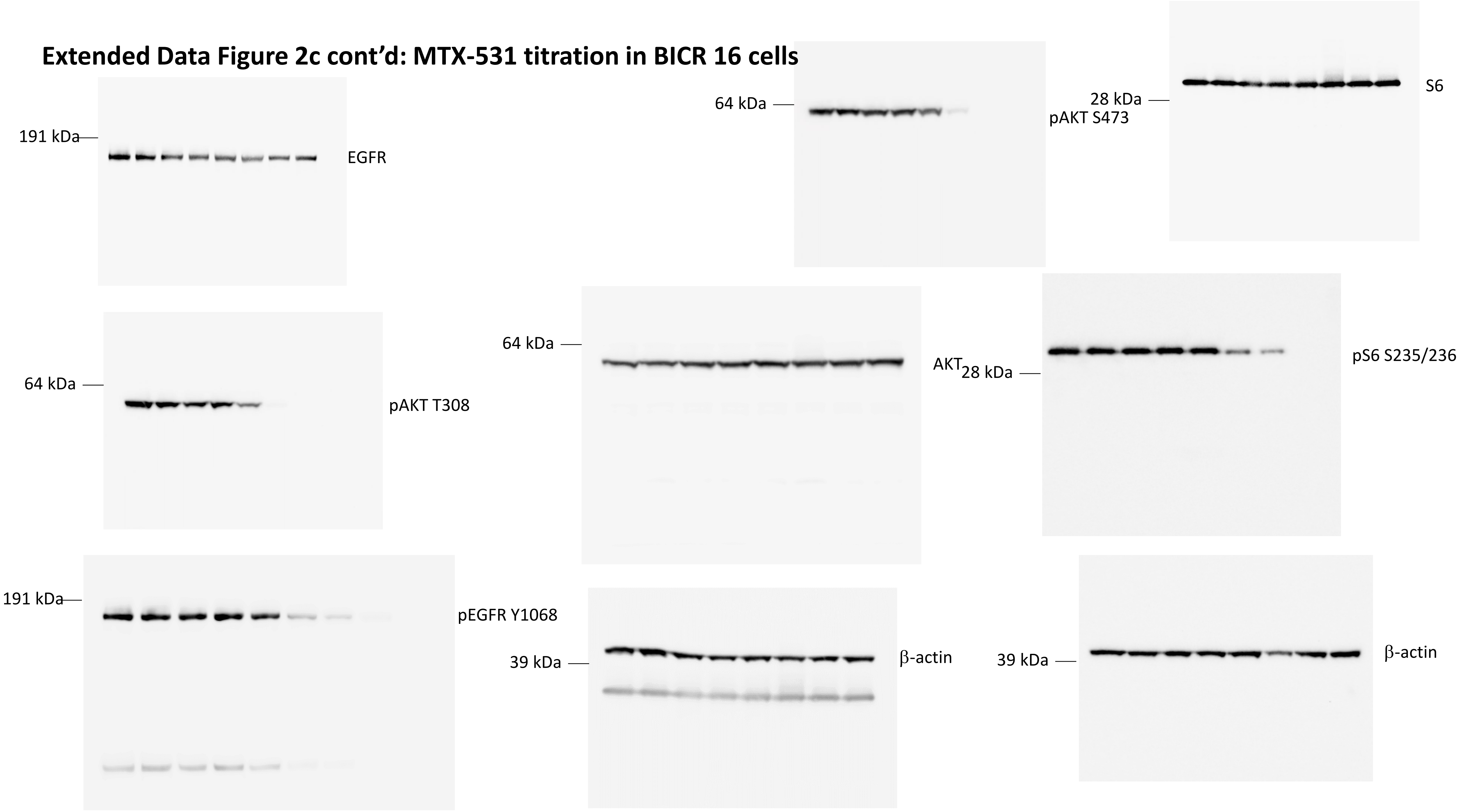

Extended Data Figure 2c cont'd: MTX-531 titration in BICR 56 cells

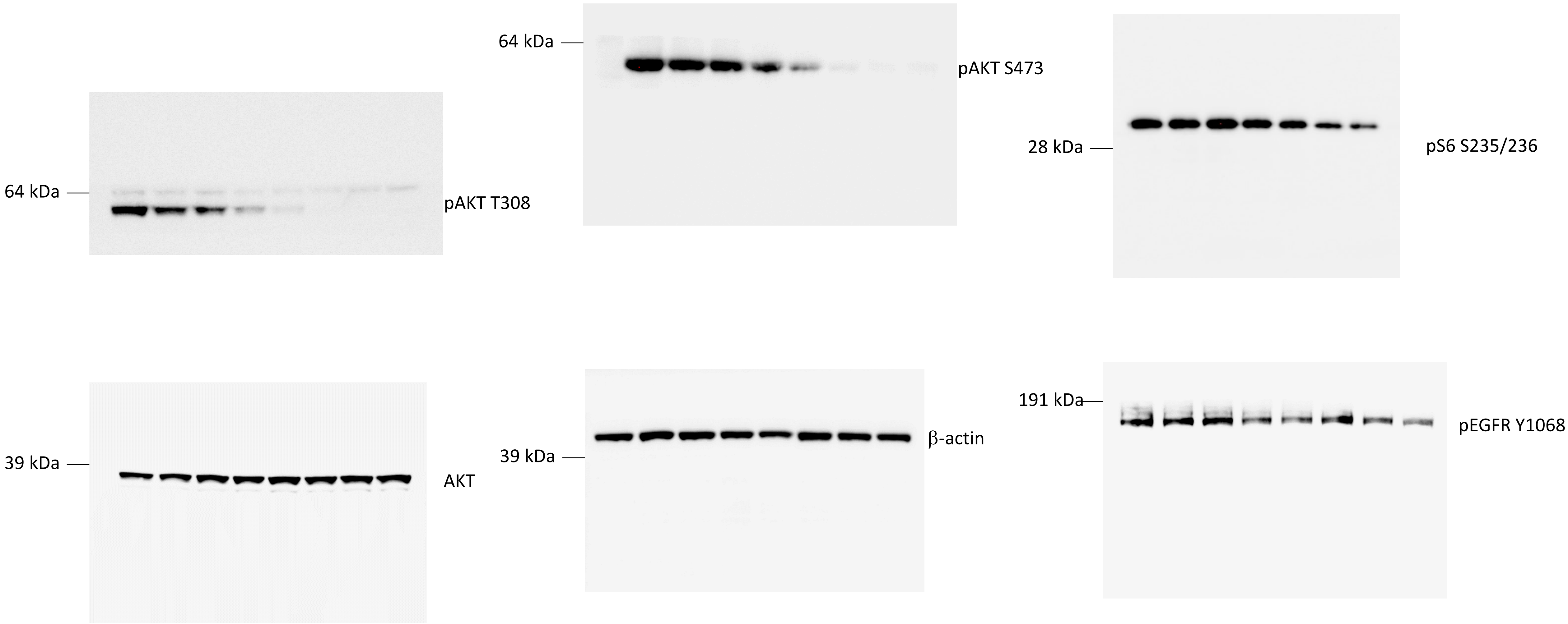

BICR 56 Titration MTX-531

Extended Data Figure 2c cont'd: MTX-531 titration in BICR 56 cells

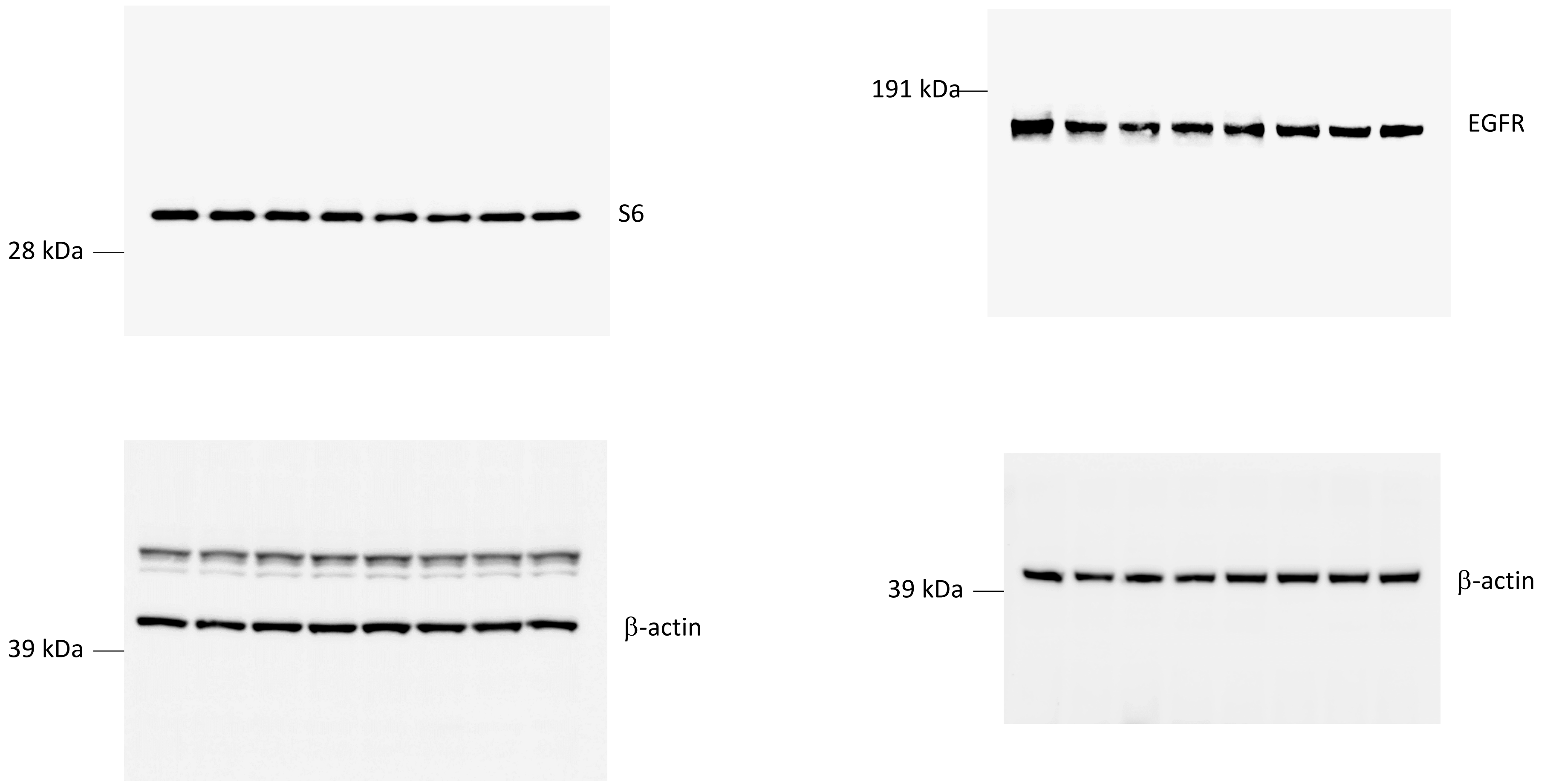

BICR 56 Titration MTX-531

Extended Data Figure 2c cont'd: MTX-531 titration in Detroit 562 cells

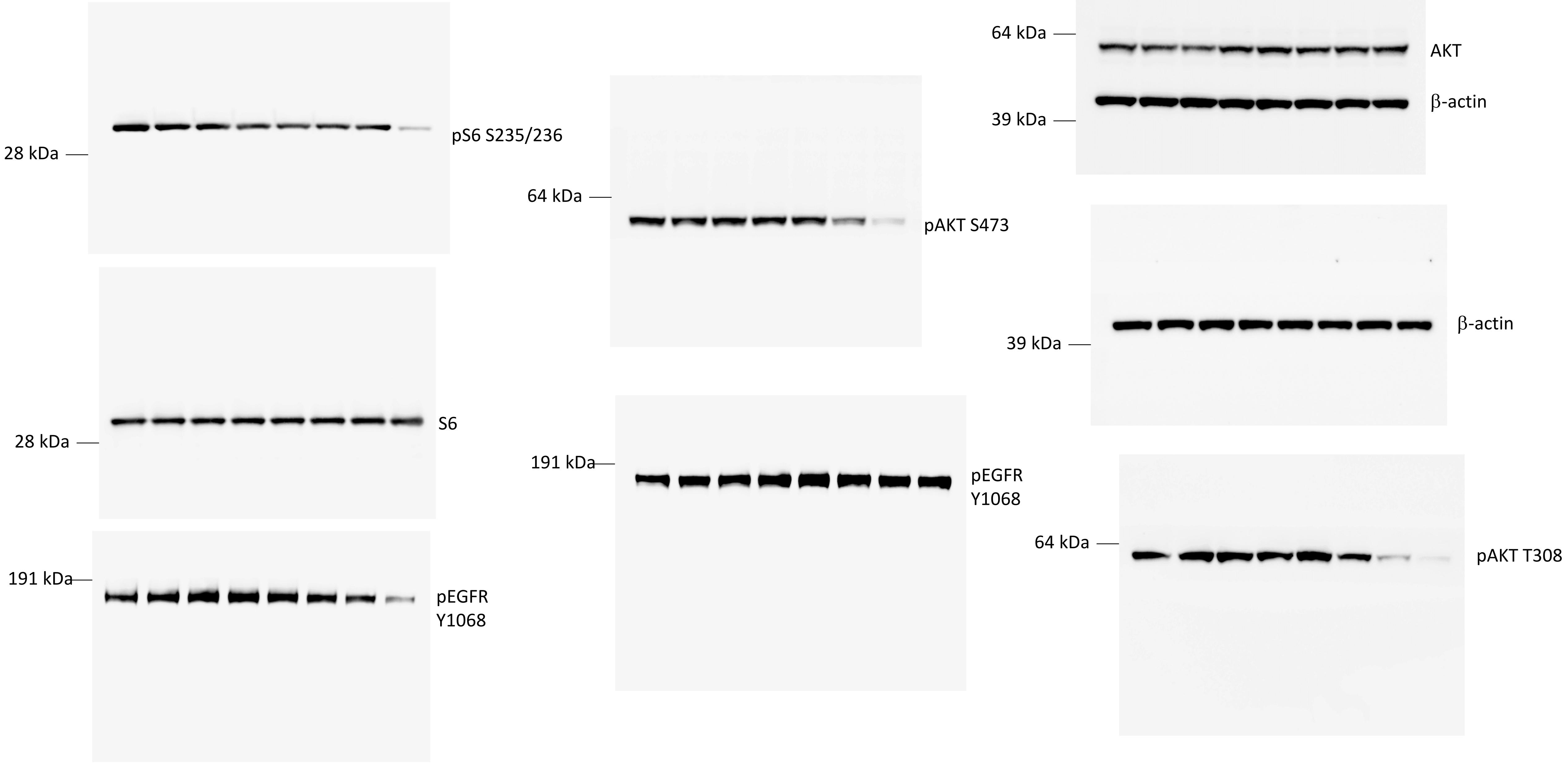

Extended Data Figure 2e cont'd: MTX-531 titration in PI3K WT cells (CAL-27 and MOC1)

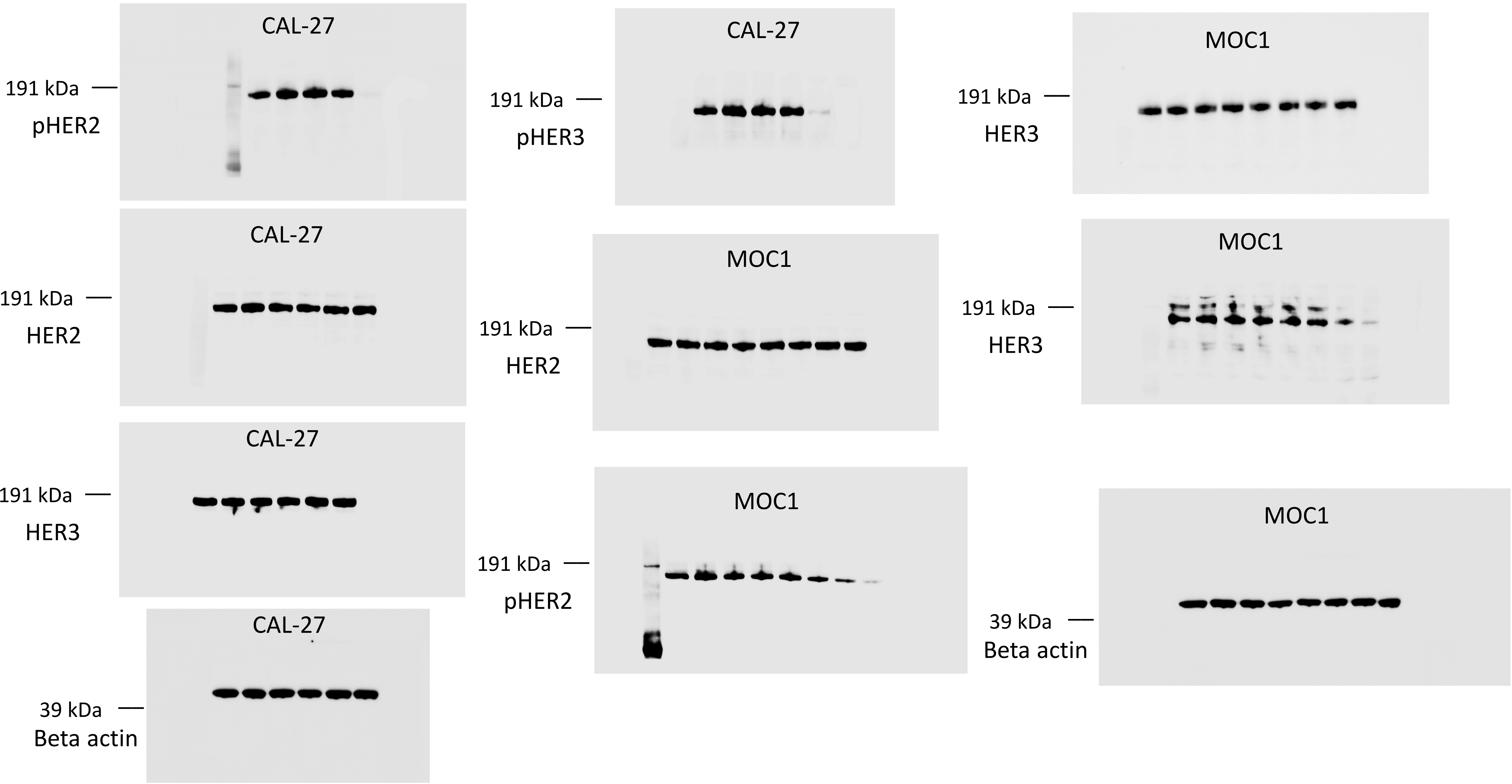

Supplement: Supplementary file 20 — Unprocessed western blots. [file 43018_2024_781_MOESM20_ESM.pdf]
