# Supplementary material for: A first-in-class selective inhibitor of EGFR and PI3K offers a single-molecule approach to targeting adaptive resistance
Source: Nat Cancer. 2024 Jul 11;5(8):1250–66. doi: 10.1038/s43018-024-00781-6 (PMC11357990; doi:10.1038/s43018-024-00781-6)

Extended Data Figure 4a: MTX-531 25 mg/kg time course PD in CAL-33

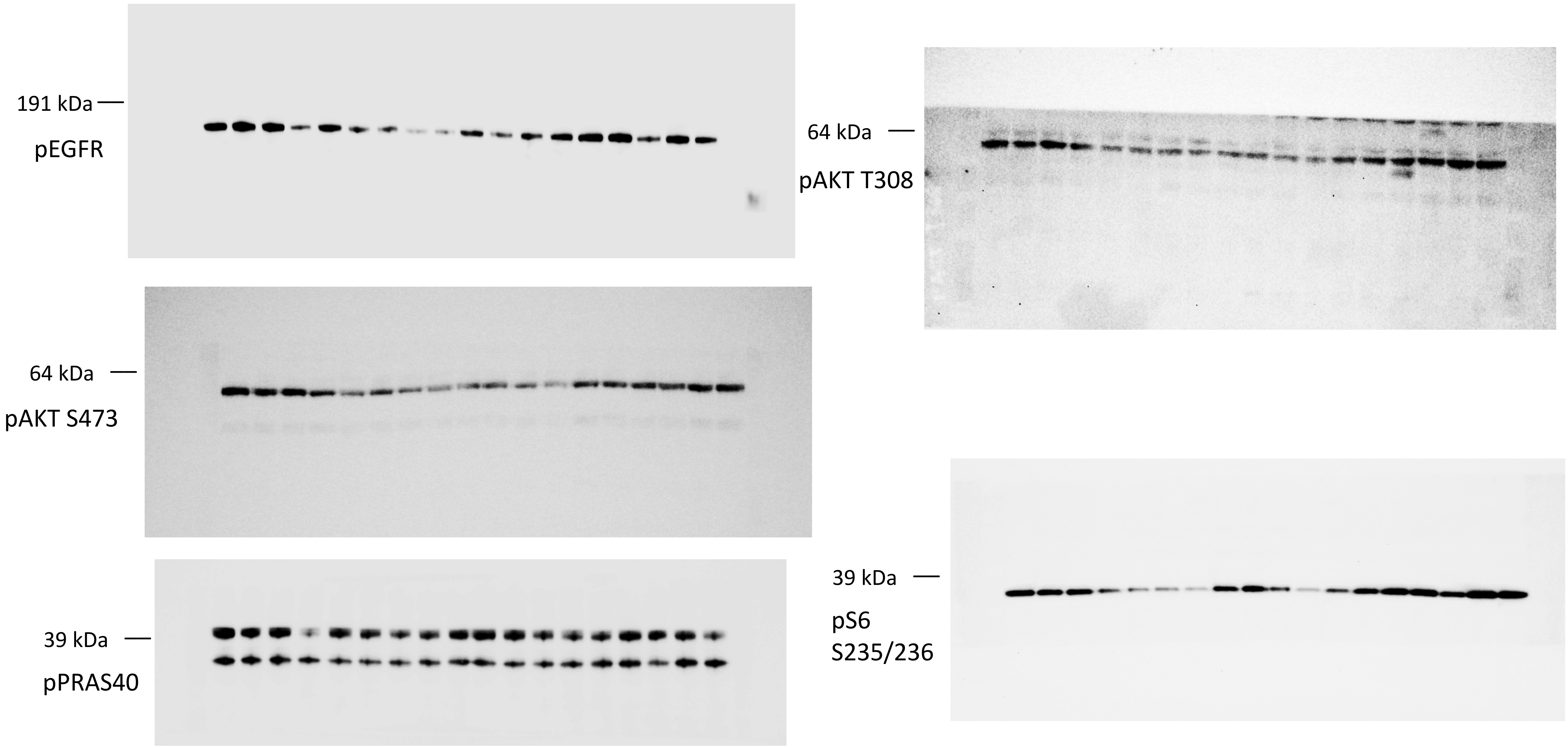

Extended Data Figure 4a cont'd: MTX-531 25 mg/kg time course PD in CAL-33

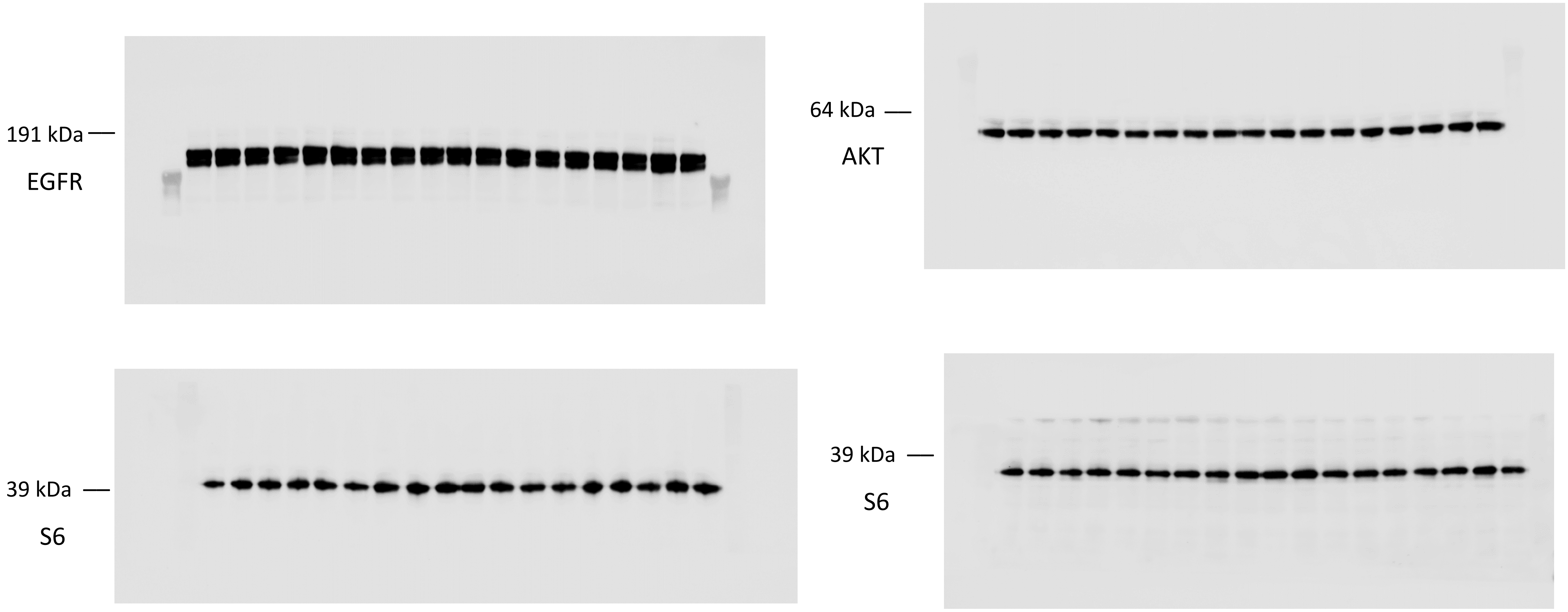

Extended Data Figure 4a cont'd: MTX-531 25 mg/kg time course PD in CAL-33

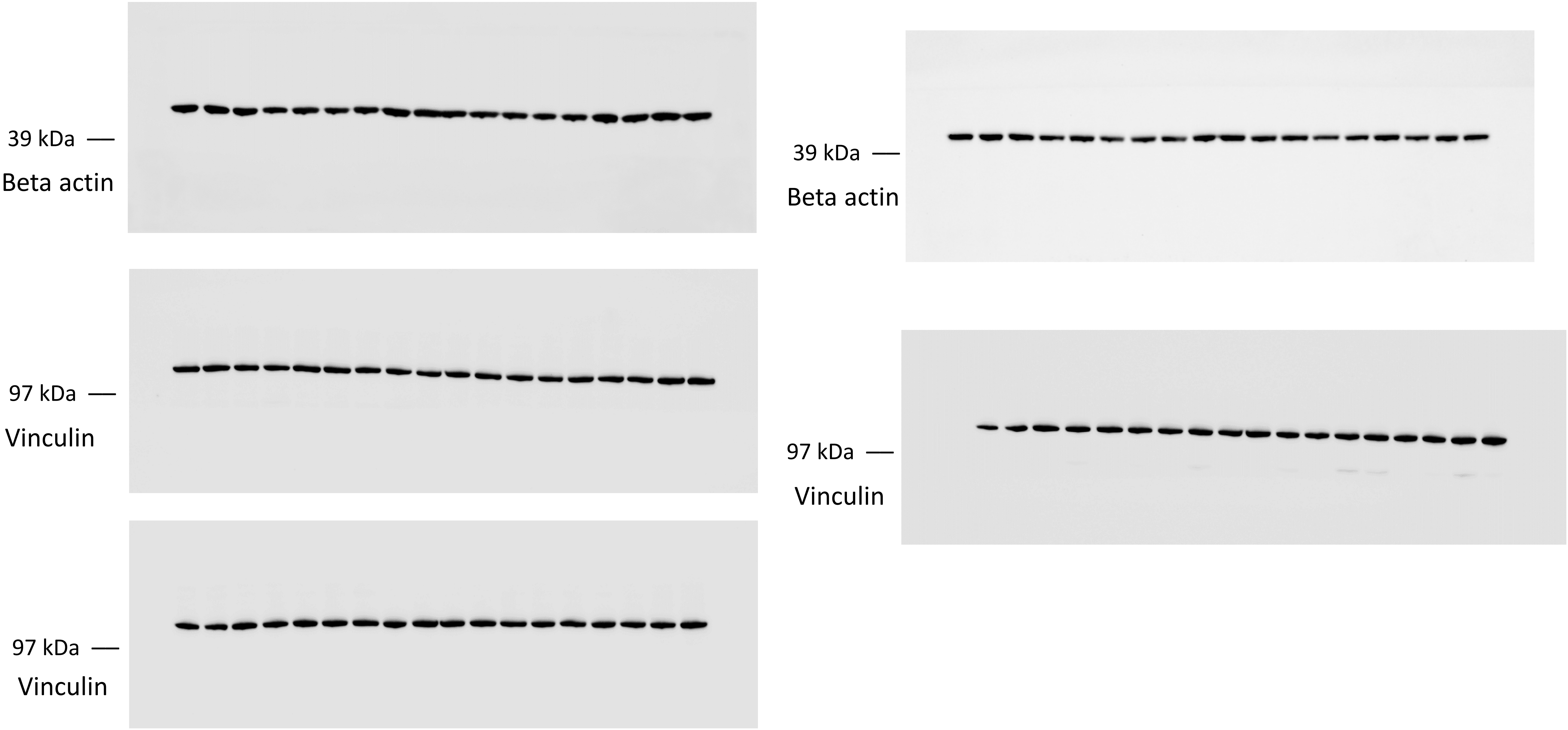

Extended Data Figure 4b: Baseline expression analysis of HNSCC PDX model panel

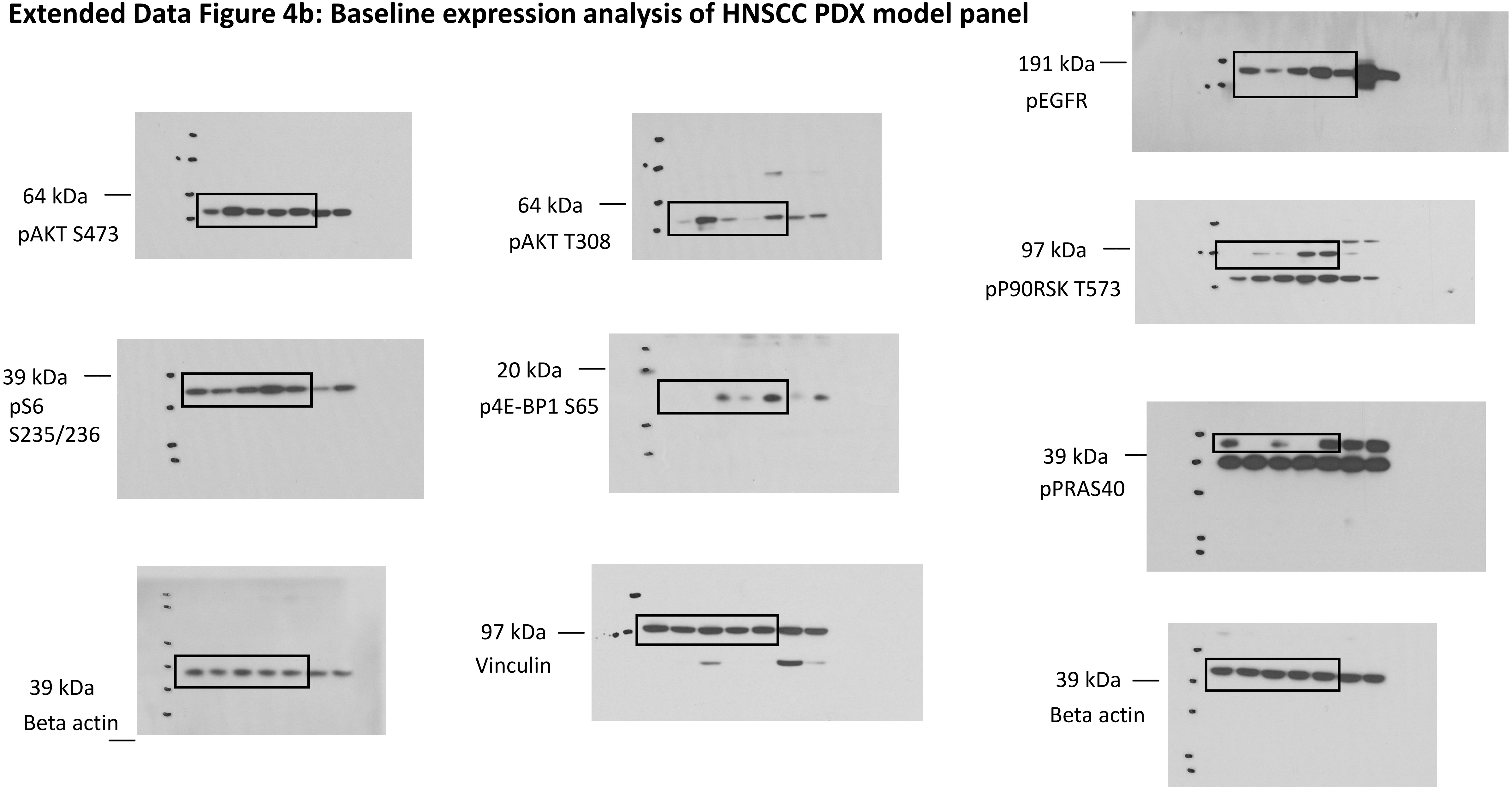

Extended Data Figure 4c: MTX-531 100 mg/kg 5-day PD in NCI 944545-341-R

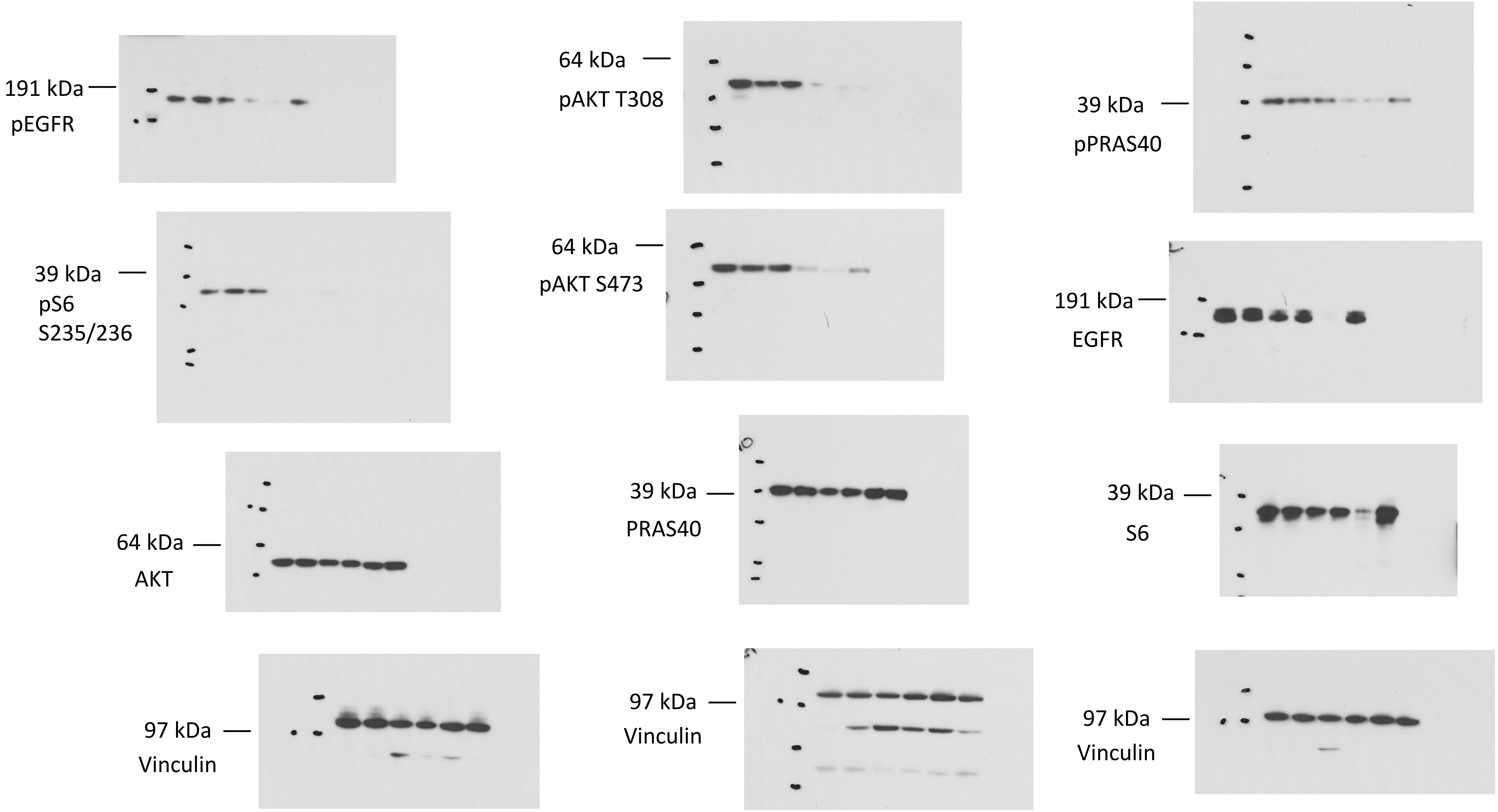

Supplement: Supplementary file 23 — Unprocessed western blots. [file 43018_2024_781_MOESM23_ESM.pdf]
