# Supplementary material for: A first-in-class selective inhibitor of EGFR and PI3K offers a single-molecule approach to targeting adaptive resistance
Source: Nat Cancer. 2024 Jul 11;5(8):1250–66. doi: 10.1038/s43018-024-00781-6 (PMC11357990; doi:10.1038/s43018-024-00781-6)
Supplement: Supplementary file 24 — Statistical source data. [file 43018_2024_781_MOESM24_ESM.pdf]

245127 Spaghetti Plots

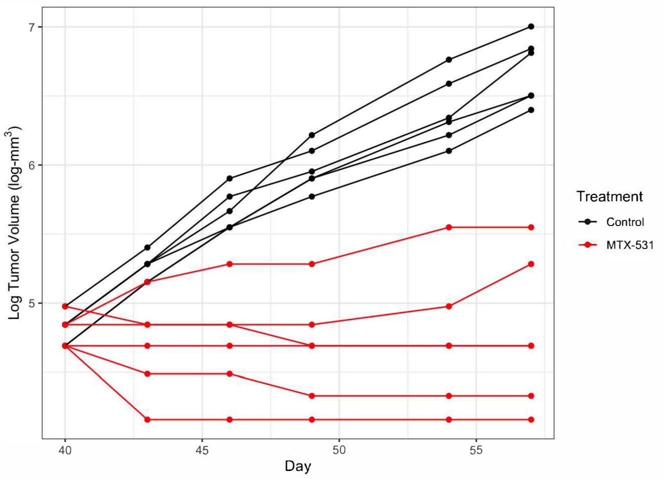

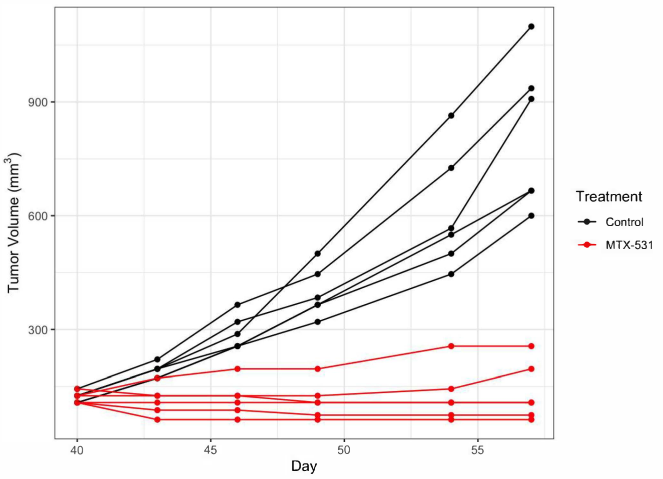

Model Fitting

Parameter	Estimate (95 % CI)
Beta_1 (Control)	0.11 (0.1, 0.12)
Beta_2 (MTX-531)	-0.00037 (-0.0088, 0.0081)

```
## Linear mixed model fit by REML ['lmerMod']
## Formula: logsize ~ day_Control + `day_MTX-531` + (1 | unique_id)
##    Data: d
##
## REML criterion at convergence: -9
##
## Scaled residuals:
##      Min       1Q   Median       3Q      Max
## -2.64212 -0.66073 -0.02554  0.37308  2.63310
##
## Random effects:
##  Groups    Name        Variance Std.Dev.
##  unique_id (Intercept) 0.07574  0.2752
##  Residual              0.02440  0.1562
## Number of obs: 72, groups:  unique_id, 12
##
## Fixed effects:
##                 Estimate Std. Error t value
## (Intercept)    4.8263557  0.0854038  56.512
## day_Control    0.1075486  0.0042943  25.045
## `day_MTX-531` -0.0003731  0.0042943  -0.087
##
## Correlation of Fixed Effects:
##             (Intr) dy_Cnt
## day_Control -0.215
## `d_MTX-531` -0.215  0.046
```

Expected Trajectories

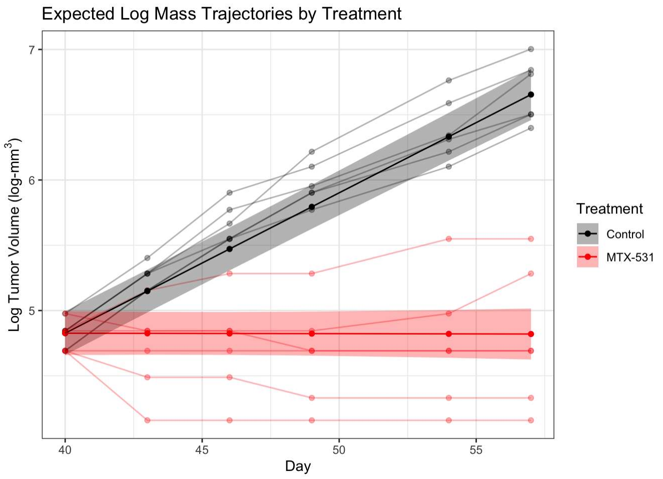

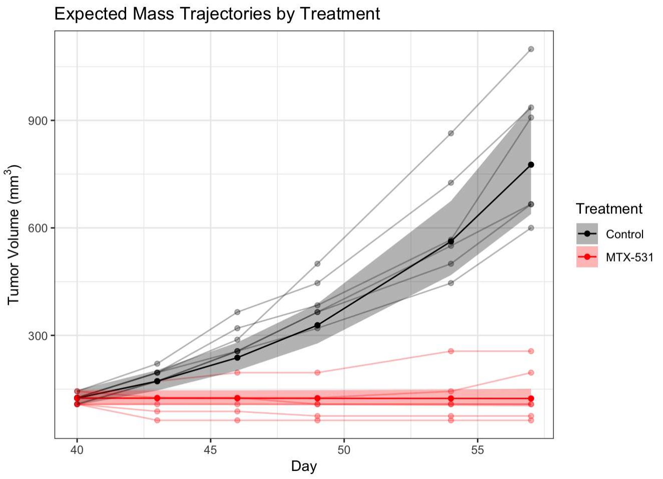

P-value

Testing $\beta_1 = \beta_2$:

Hypothesis	Wald Statistic	P-values
Beta Control = Beta MTX-531 vs. Beta Control > Beta MTX-531	18.2	p < 0.0001

354836

Spaghetti Plots

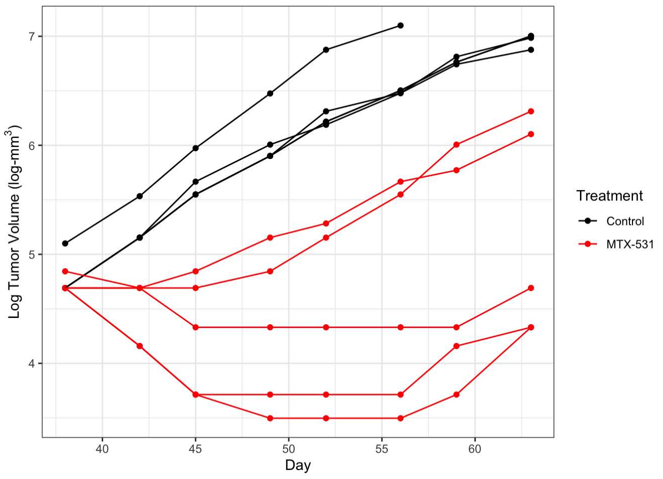

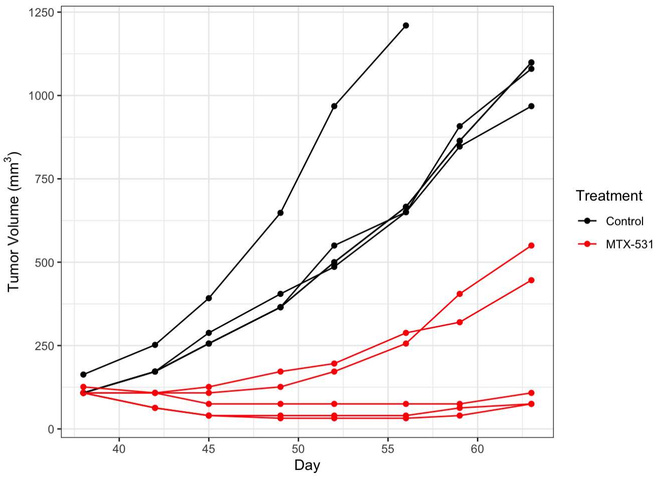

Model Fitting

Parameter	Estimate (95 % CI)
Beta_1 (Control)	0.096 (0.083, 0.11)
Beta_2 (MTX-531)	0.017 (0.0048, 0.029)

```
## Linear mixed model fit by REML ['lmerMod']
## Formula: logsize ~ day_Control + `day_MTX-531` + (1 | unique_id)
##    Data: d
##
## REML criterion at convergence: 91.9
##
## Scaled residuals:
##      Min       1Q   Median       3Q      Max
## -1.61656 -0.54223 -0.05248  0.33893  2.98776
##
## Random effects:
##  Groups    Name        Variance Std.Dev.
##  unique_id (Intercept) 0.2816   0.5307
##  Residual              0.1054   0.3247
## Number of obs: 78, groups:  unique_id, 10
##
## Fixed effects:
##               Estimate Std. Error t value
## (Intercept)   4.629064   0.180795  25.604
## day_Control   0.095845   0.006586  14.552
## `day_MTX-531` 0.016664   0.006200   2.688
##
## Correlation of Fixed Effects:
##             (Intr) dy_Cnt
## day_Control -0.226
## `d_MTX-531` -0.226  0.051
```

Expected Trajectories

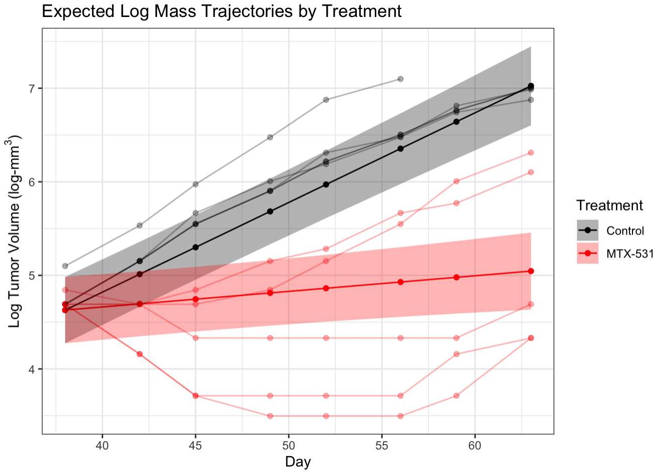

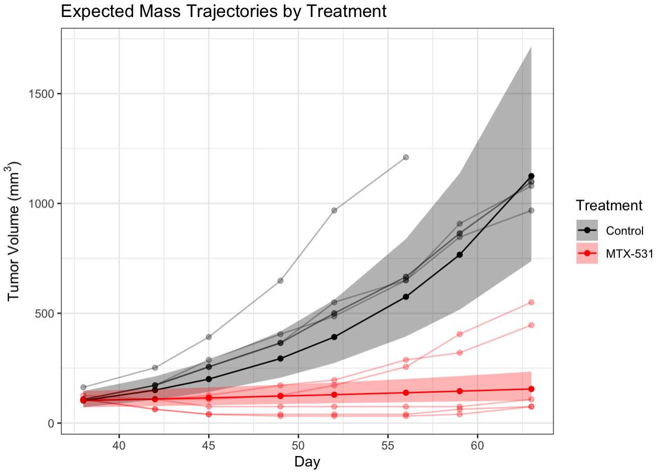

P-value

Testing $\beta_1 = \beta_2$:

Hypothesis	Wald Statistic	P-values
Beta Control = Beta MTX-531 vs. Beta Control > Beta MTX-531	8.99	p < 0.001

455876

Spaghetti Plots

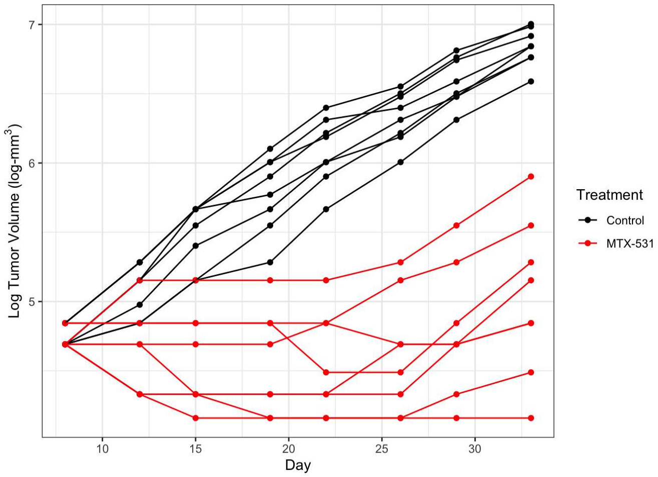

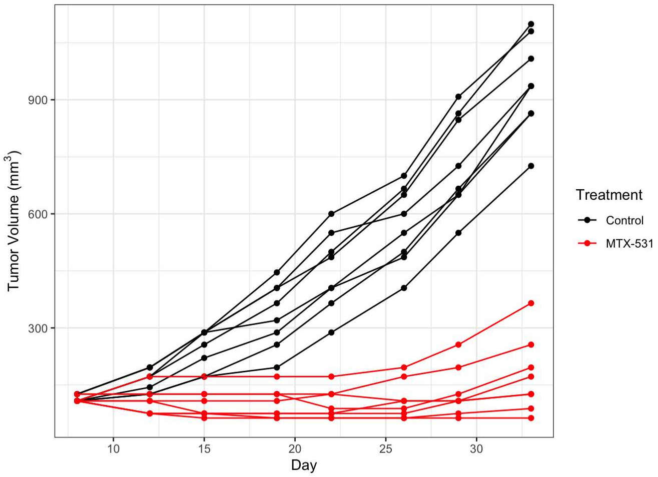

Model Fitting

Parameter	Estimate (95 % CI)
Beta_1 (Control)	0.085 (0.08, 0.09)
Beta_2 (MTX-531)	0.0083 (0.0028, 0.014)

```
## Linear mixed model fit by REML ['lmerMod']
## Formula: logsize ~ day_Control + `day_MTX-531` + (1 | unique_id)
##    Data: d
##
## REML criterion at convergence: 3.1
##
## Scaled residuals:
##      Min       1Q   Median       3Q      Max
## -2.29902 -0.70817 -0.01173  0.43004  3.01251
##
## Random effects:
##  Groups    Name        Variance Std.Dev.
##  unique_id (Intercept) 0.07577  0.2753
##  Residual              0.03568  0.1889
## Number of obs: 128, groups:  unique_id, 16
##
## Fixed effects:
##               Estimate Std. Error t value
## (Intercept)   4.695349   0.075379  62.290
## day_Control   0.085095   0.002836  30.008
## `day_MTX-531` 0.008321   0.002836   2.935
##
## Correlation of Fixed Effects:
##             (Intr) dy_Cnt
## day_Control -0.250
## `d_MTX-531` -0.250  0.062
```

Expected Trajectories

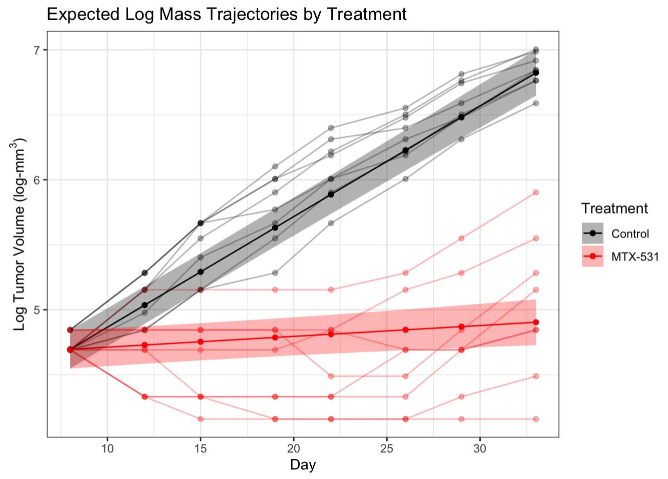

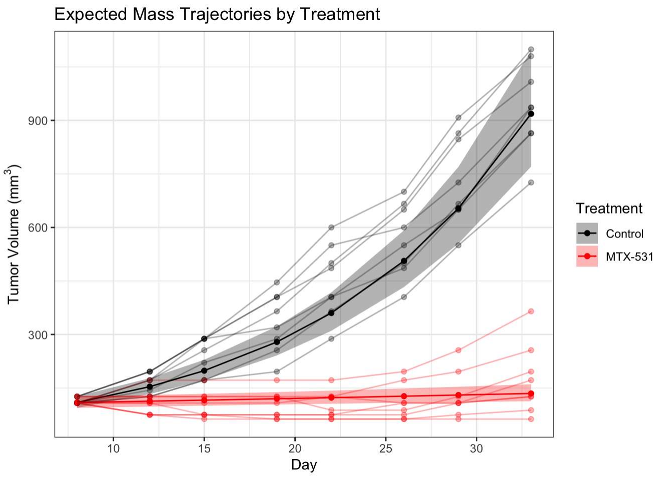

P-value

Testing $\beta_1 = \beta_2$:

Hypothesis	Wald Statistic	P-values
Beta Control = Beta MTX-531 vs. Beta Control > Beta MTX-531	19.8	p < 0.0001

848979

Spaghetti Plots

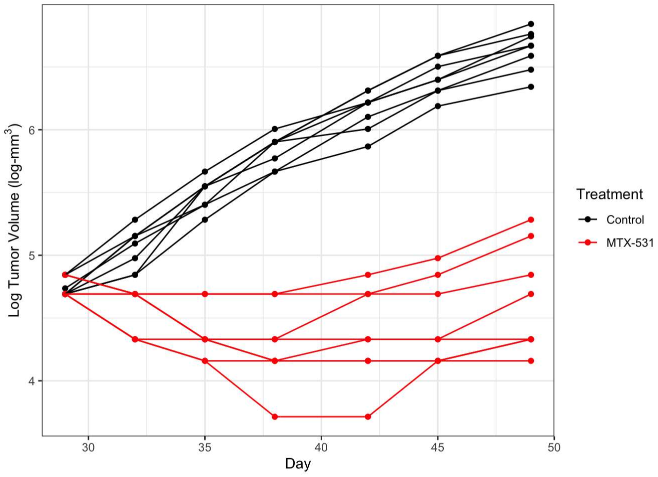

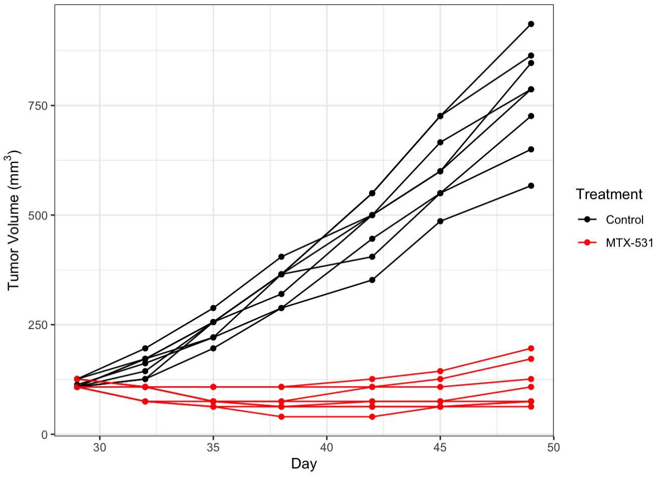

Model Fitting

Parameter	Estimate (95 % CI)
Beta_1 (Control)	0.1 (0.093, 0.11)
Beta_2 (MTX-531)	-0.0076 (-0.015, -0.00035)

```
## Linear mixed model fit by REML ['lmerMod']
## Formula: logsize ~ day_Control + `day_MTX-531` + (1 | unique_id)
##    Data: d
##
## REML criterion at convergence: 5.5
##
## Scaled residuals:
##      Min       1Q   Median       3Q      Max
## -2.45086 -0.62279 -0.00772  0.55096  2.83098
##
## Random effects:
##  Groups    Name        Variance Std.Dev.
##  unique_id (Intercept) 0.04434  0.2106
##  Residual              0.03753  0.1937
## Number of obs: 112, groups:  unique_id, 16
##
## Fixed effects:
##                Estimate Std. Error t value
## (Intercept)    4.685018   0.061648  75.996
## day_Control    0.099694   0.003710  26.871
## `day_MTX-531` -0.007608   0.003710  -2.051
##
## Correlation of Fixed Effects:
##             (Intr) dy_Cnt
## day_Control -0.317
## `d_MTX-531` -0.317  0.100
```

Expected Trajectories

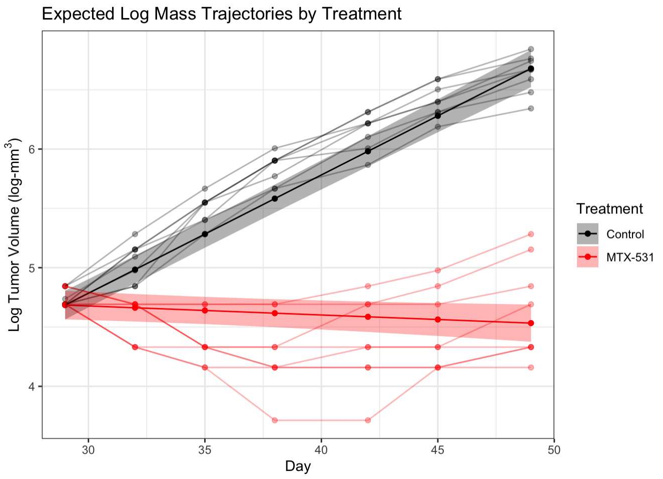

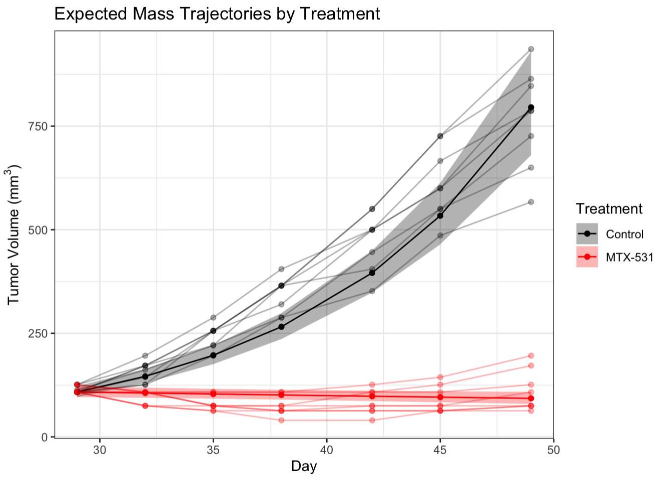

P-value

Testing $\beta_1 = \beta_2$:

Hypothesis	Wald Statistic	P-values
Beta Control = Beta MTX-531 vs. Beta Control > Beta MTX-531	21.6	p < 0.0001

944545

Spaghetti Plots

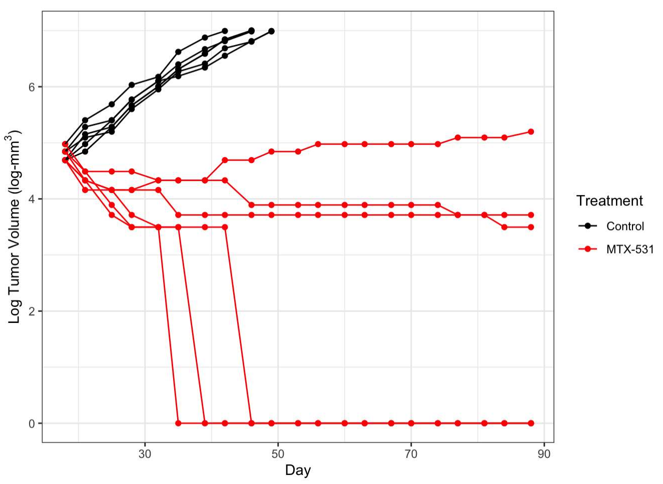

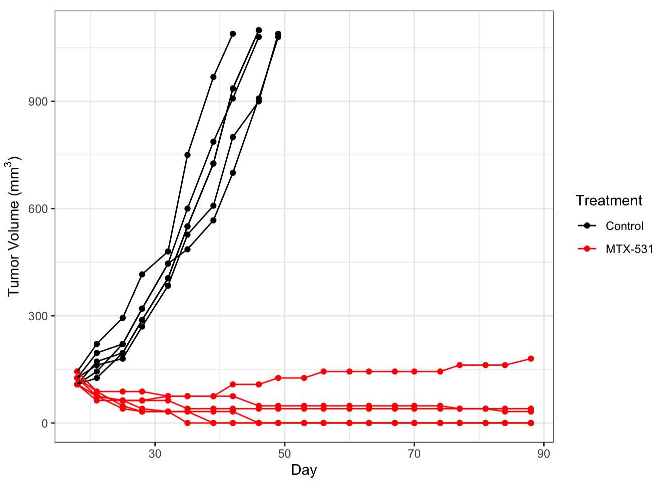

Model Fitting

Parameter	Estimate (95 % CI)
Beta_1 (Control)	0.082 (0.057, 0.11)
Beta_2 (MTX-531)	-0.037 (-0.044, -0.03)

```
## Linear mixed model fit by REML ['lmerMod']
## Formula: logsize ~ day_Control + `day_MTX-531` + (1 | unique_id)
##    Data: d
##
## REML criterion at convergence: 535.3
##
## Scaled residuals:
##      Min       1Q   Median       3Q      Max
## -1.88548 -0.52766 -0.04116  0.29835  2.90655
##
## Random effects:
##  Groups    Name        Variance Std.Dev.
##  unique_id (Intercept) 1.5030   1.2260
##  Residual              0.8439   0.9186
## Number of obs: 181, groups:  unique_id, 12
##
## Fixed effects:
##               Estimate Std. Error t value
## (Intercept)    4.40230    0.37923  11.608
## day_Control    0.08213    0.01295   6.342
## `day_MTX-531` -0.03687    0.00380  -9.701
##
## Correlation of Fixed Effects:
##             (Intr) dy_Cnt
## day_Control -0.244
## `d_MTX-531` -0.188  0.046
```

Expected Trajectories

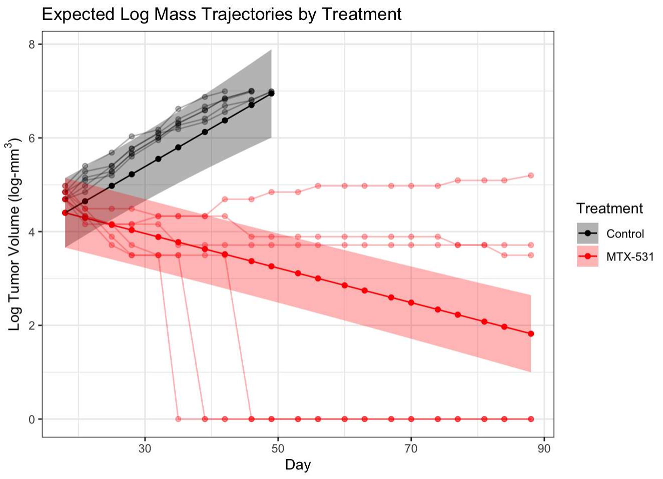

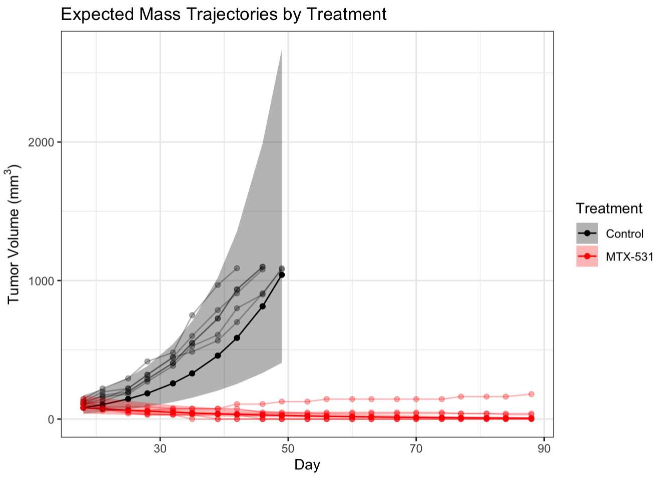

P-value

Testing $\beta_1 = \beta_2$:

Hypothesis	Wald Statistic	P-values
Beta Control = Beta MTX-531 vs. Beta Control > Beta MTX-531	8.93	p < 0.0001

```
## Linear mixed model fit by REML ['lmerMod']
## Formula: logsize ~ day_Control + `day_MTX-531` + (1 | unique_id)
##    Data: d
##
## REML criterion at convergence: -19.5
##
## Scaled residuals:
##     Min      1Q  Median      3Q     Max
## -2.2179 -0.6618 -0.1495  0.7071  1.8502
##
## Random effects:
##  Groups    Name        Variance Std.Dev.
##  unique_id (Intercept) 0.03503  0.1872
##  Residual              0.01771  0.1331
## Number of obs: 50, groups:  unique_id, 10
##
## Fixed effects:
##               Estimate Std. Error t value
## (Intercept)   4.681805   0.067093  69.781
## day_Control   0.184154   0.007861  23.426
## `day_MTX-531` 0.030386   0.007861   3.865
##
## Correlation of Fixed Effects:
##             (Intr) dy_Cnt
## day_Control -0.278
## `d_MTX-531` -0.278  0.077
```

Expected Trajectories

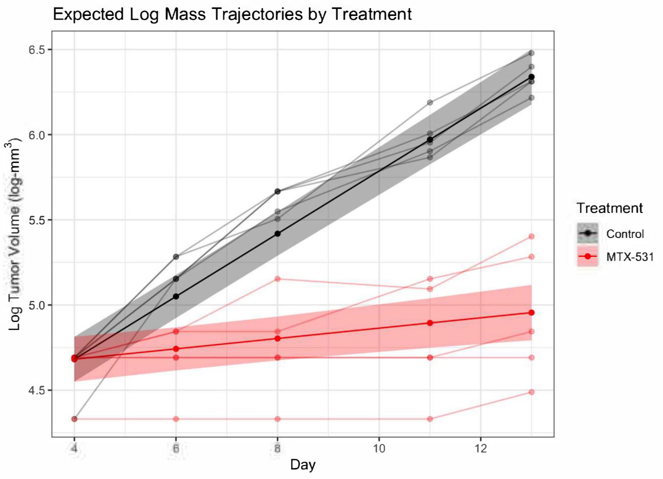

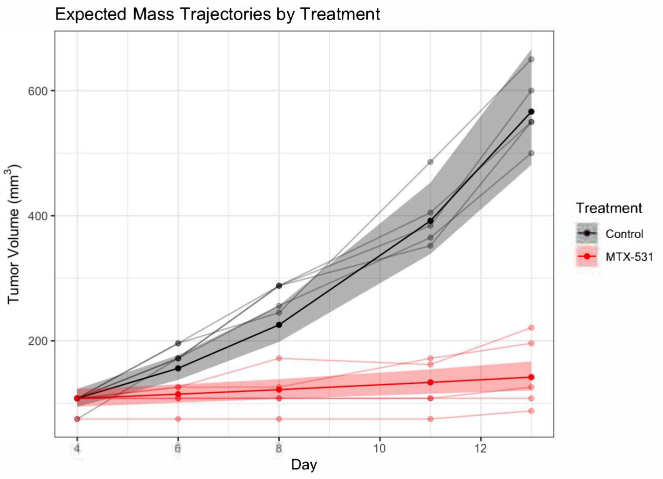

P-Value(s)

Hypothesis	Wald Statistic	P-values
Beta Control = Beta MTX-531 vs. Beta Control > Beta MTX-531	14.4	$p < 0.0001$
