# Supplementary material for: A first-in-class selective inhibitor of EGFR and PI3K offers a single-molecule approach to targeting adaptive resistance
Source: Nat Cancer. 2024 Jul 11;5(8):1250–66. doi: 10.1038/s43018-024-00781-6 (PMC11357990; doi:10.1038/s43018-024-00781-6)

## Extended Data Figure 6a: MTX-531, Alpelisib, Erlotinib titrations in KPC cells

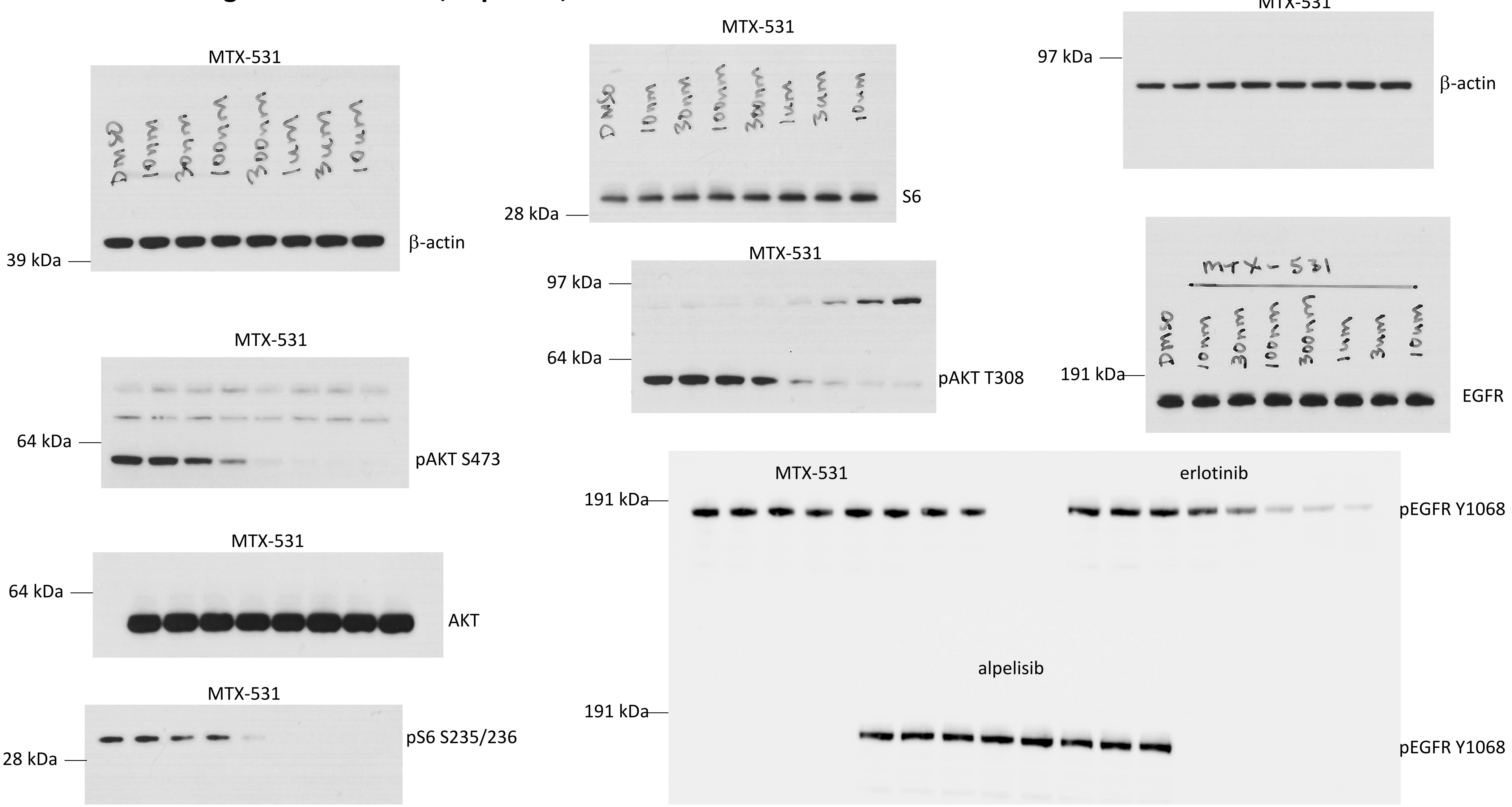

## Extended Data Figure 6a cont'd: MTX-531, Alpelisib, Erlotinib titrations in KPC cells

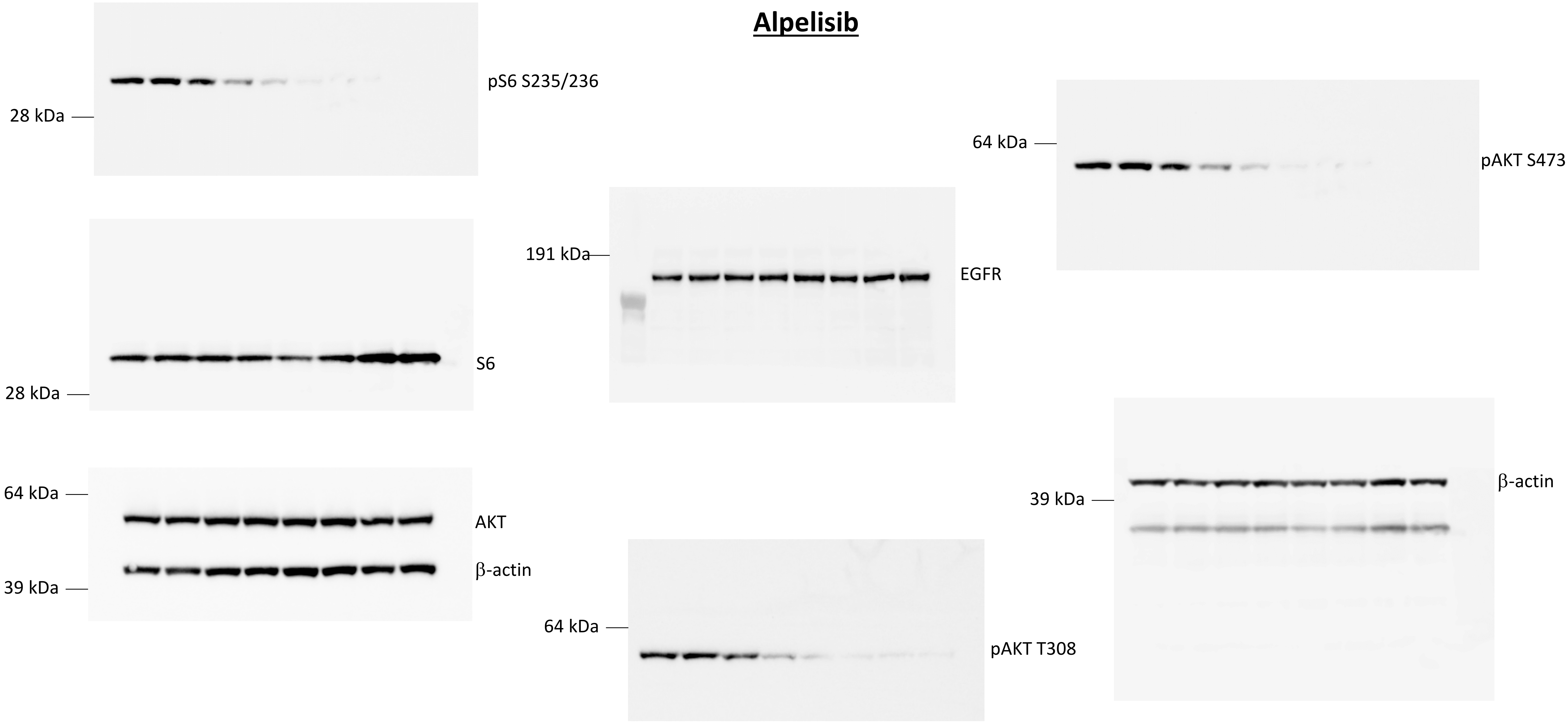

# Extended Data Figure 6a cont'd: MTX-531, Alpelisib, Erlotinib titrations in KPC cells

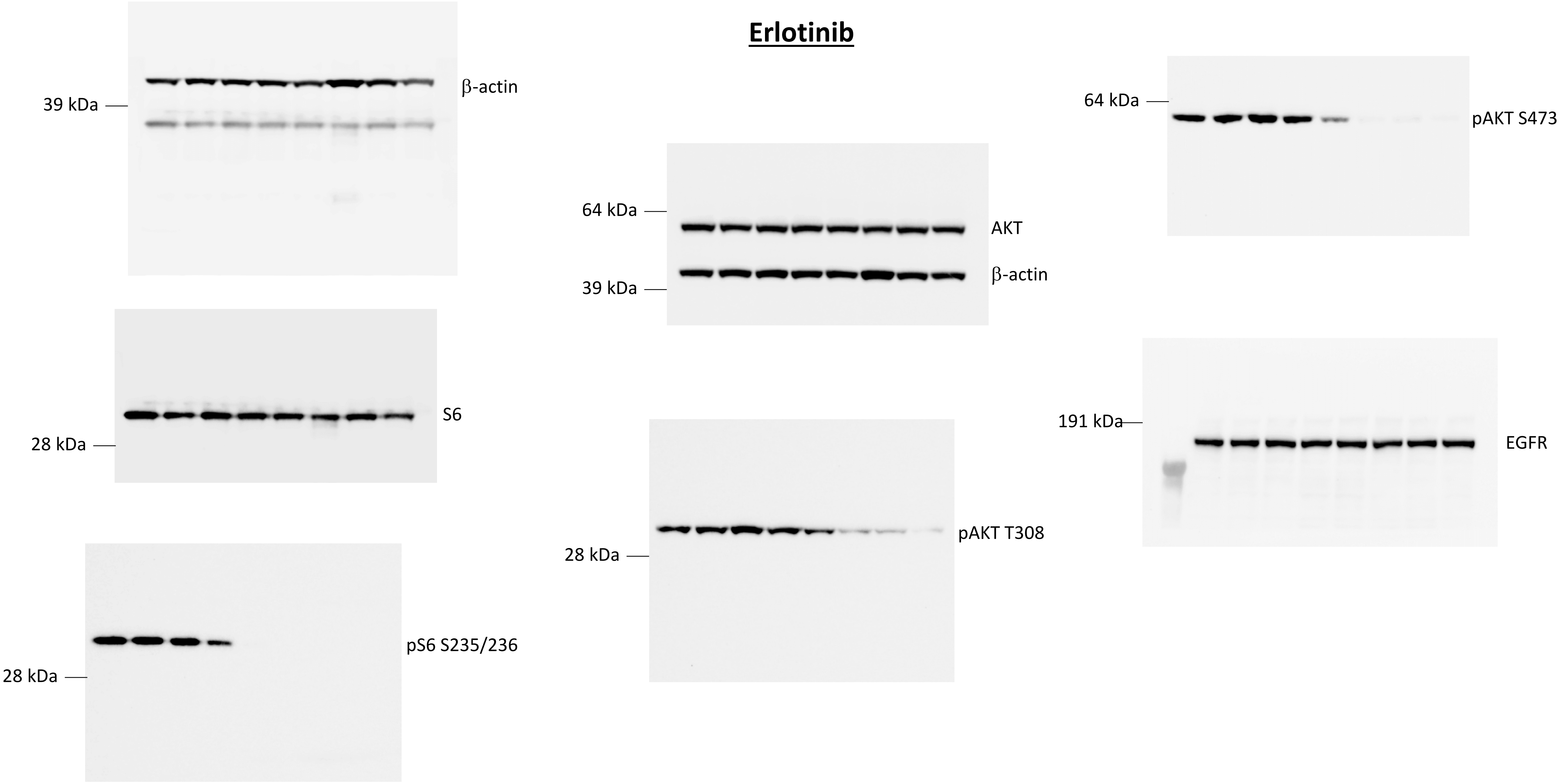

# Extended Data Figure 6b: MTX-531 2- and 24-hour PD in KPC

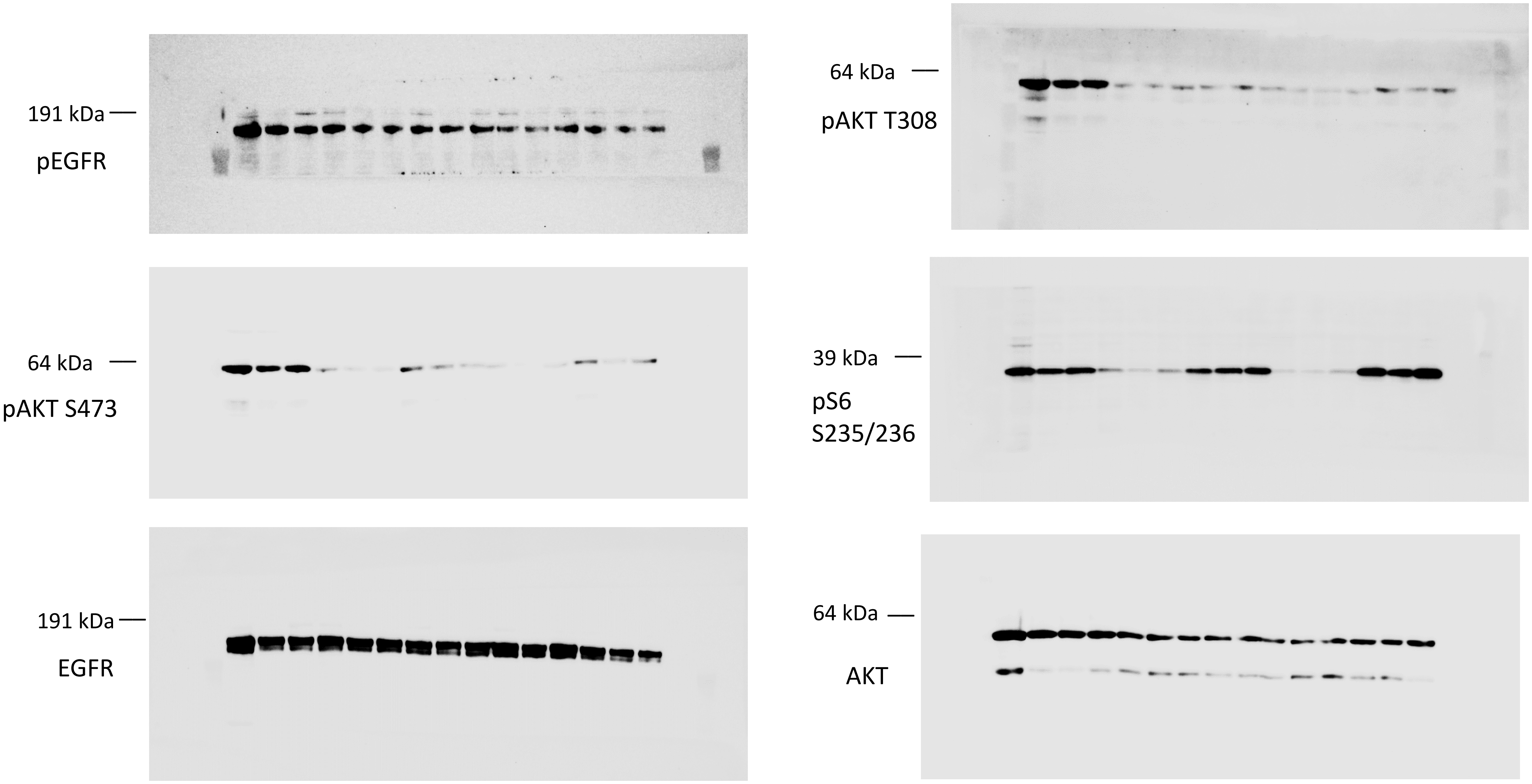

**Extended Data Figure 6b cont'd: MTX-531 2- and 24-hour PD in KPC**

39 kDa —

S6

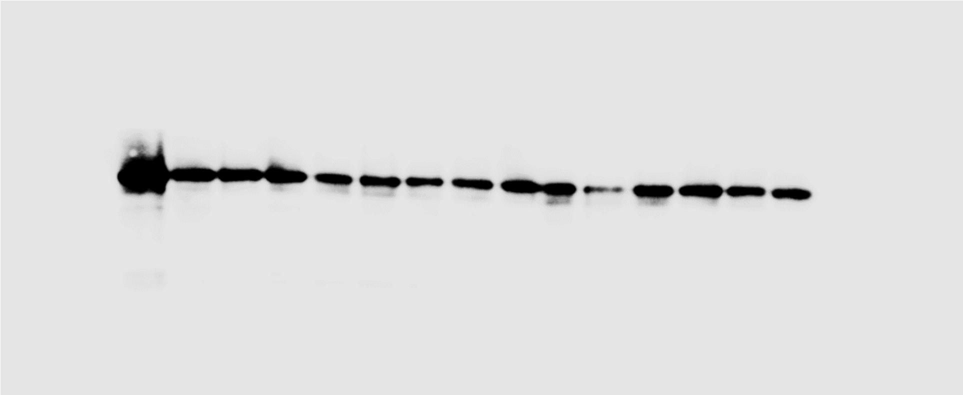

39 kDa —

Beta actin

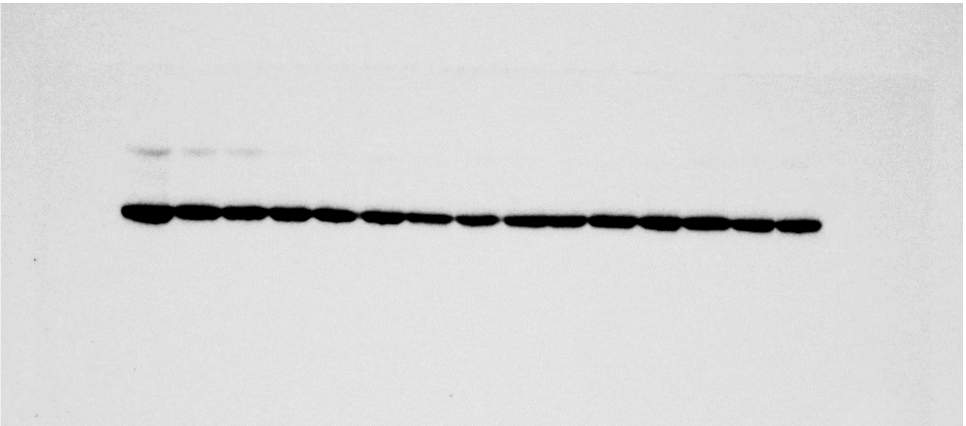

39 kDa —

Beta actin

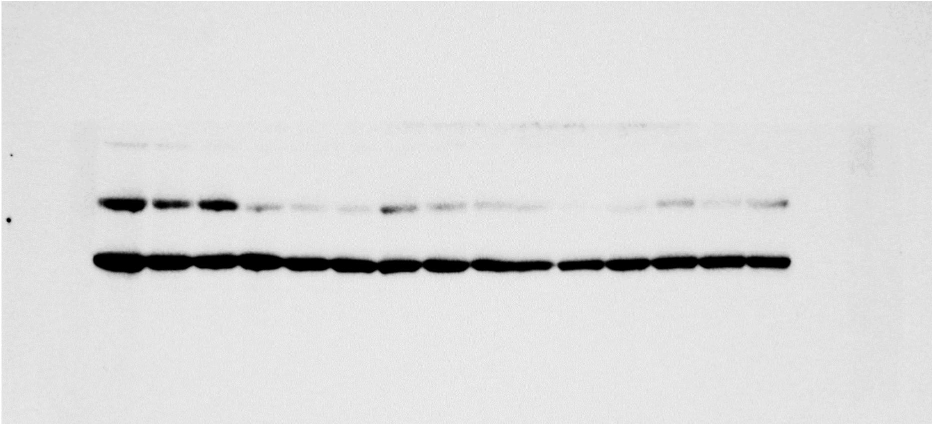

39 kDa —

Beta actin

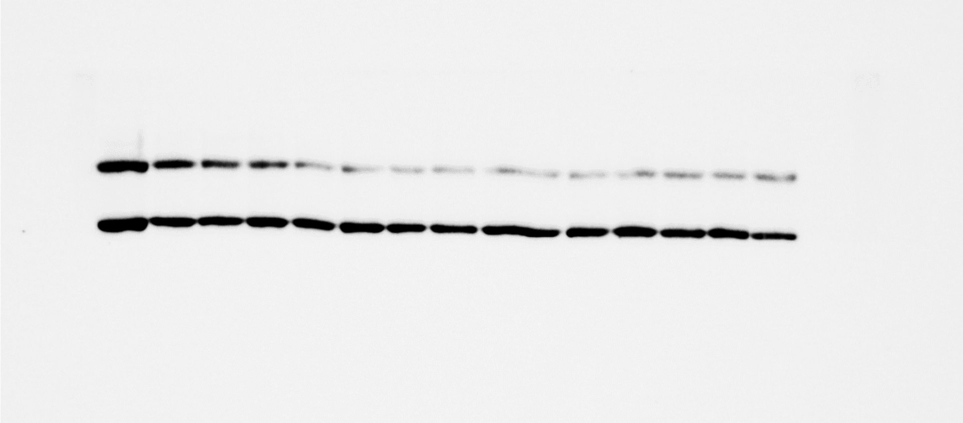

39 kDa —

Beta actin

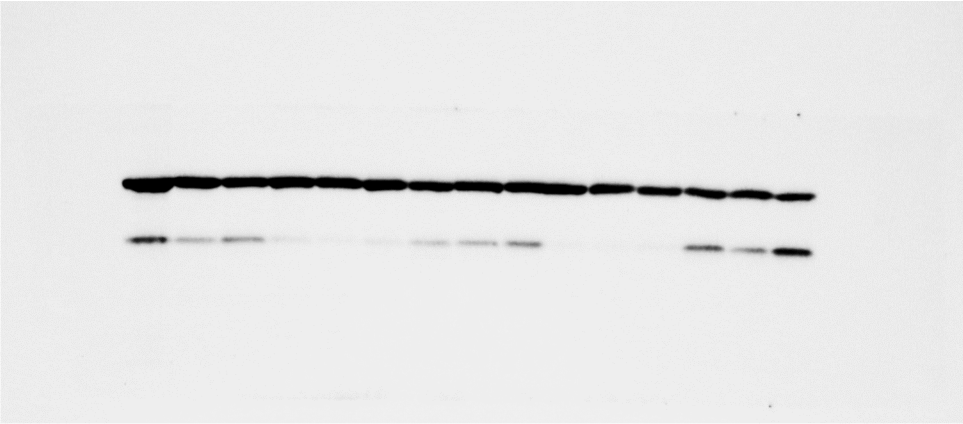

## Extended Data Figure 6c: MTX-531 2-hour PD in normal tissue

### Liver tissue

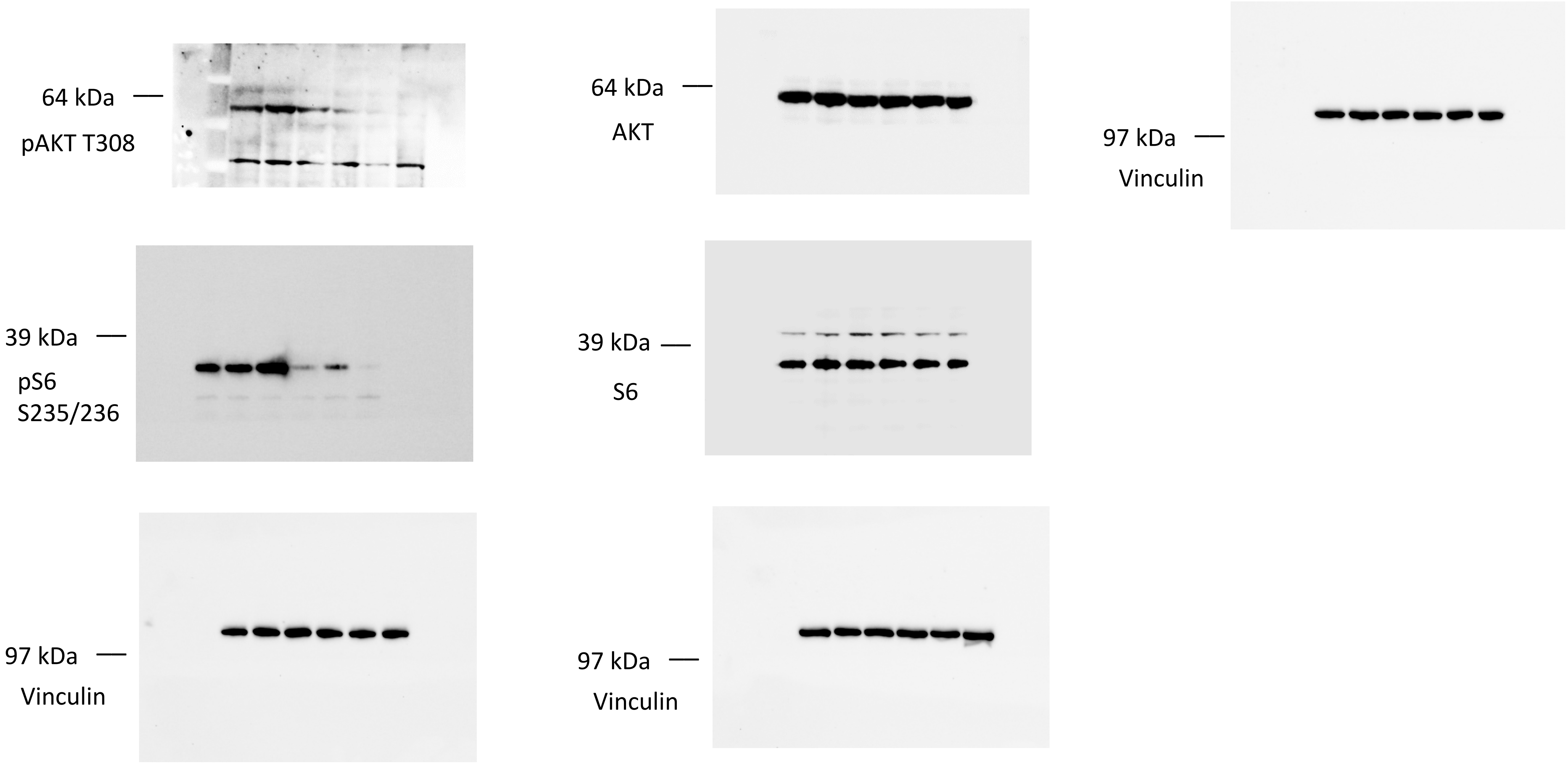

**Extended Data Figure 6c cont'd: MTX-531 2-hour PD in normal tissue**

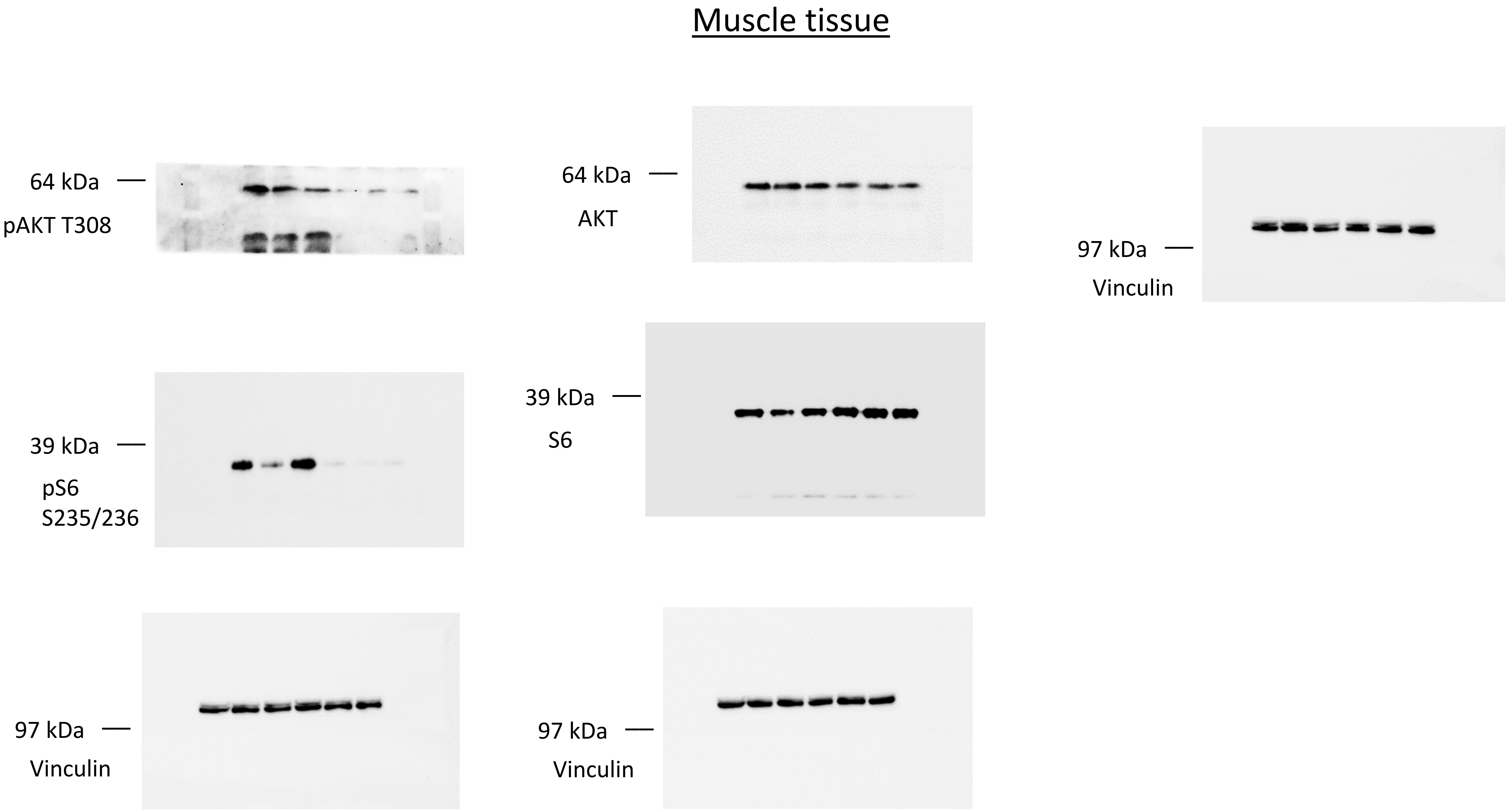

Supplement: Supplementary file 27 — Unprocessed western blots. [file 43018_2024_781_MOESM27_ESM.pdf]
